# Two Different Rickettsial Bacteria Invading *Volvox carteri*

**DOI:** 10.1371/journal.pone.0116192

**Published:** 2015-02-11

**Authors:** Kaoru Kawafune, Yuichi Hongoh, Takashi Hamaji, Tomoaki Sakamoto, Tetsuya Kurata, Shunsuke Hirooka, Shin-ya Miyagishima, Hisayoshi Nozaki

**Affiliations:** 1 Department of Biological Sciences, Graduate School of Science, University of Tokyo, Bunkyo-ku, Tokyo, Japan; 2 Department of Biological Sciences, Graduate School of Bioscience and Biotechnology, Tokyo Institute of Technology, Ookayama, Meguro-ku, Tokyo, Japan; 3 Donald Danforth Plant Science Center, Saint Louis, Missouri, United States of America; 4 Plant Global Education Project, Graduate School of Biological Sciences, Nara Institute of Science and Technology, Ikoma, Nara, Japan; 5 Center for Frontier Research, National Institute of Genetics, Mishima, Shizuoka, Japan; UC Irvine, UNITED STATES

## Abstract

**Background:**

Bacteria of the family *Rickettsiaceae* are principally associated with arthropods. Recently, endosymbionts of the *Rickettsiaceae* have been found in non-phagotrophic cells of the volvocalean green algae *Carteria cerasiformis*, *Pleodorina japonica*, and *Volvox carteri*. Such endosymbionts were present in only *C. cerasiformis* strain NIES-425 and *V. carteri* strain UTEX 2180, of various strains of *Carteria* and *V. carteri* examined, suggesting that rickettsial endosymbionts may have been transmitted to only a few algal strains very recently. However, in preliminary work, we detected a sequence similar to that of a rickettsial gene in the nuclear genome of *V. carteri* strain EVE.

**Methodology/Principal Findings:**

Here we explored the origin of the rickettsial gene-like sequences in the endosymbiont-lacking *V. carteri* strain EVE, by performing comparative analyses on 13 strains of *V. carteri*. By reference to our ongoing genomic sequence of rickettsial endosymbionts in *C. cerasiformis* strain NIES-425 cells, we confirmed that an approximately 9-kbp DNA sequence encompassing a region similar to that of four rickettsial genes was present in the nuclear genome of *V. carteri* strain EVE. Phylogenetic analyses, and comparisons of the synteny of rickettsial gene-like sequences from various strains of *V. carteri*, indicated that the rickettsial gene-like sequences in the nuclear genome of *V. carteri* strain EVE were closely related to rickettsial gene sequences of *P. japonica*, rather than those of *V. carteri* strain UTEX 2180.

**Conclusion/Significance:**

At least two different rickettsial organisms may have invaded the *V. carteri* lineage, one of which may be the direct ancestor of the endosymbiont of *V. carteri* strain UTEX 2180, whereas the other may be closely related to the endosymbiont of *P. japonica*. Endosymbiotic gene transfer from the latter rickettsial organism may have occurred in an ancestor of *V. carteri*. Thus, the rickettsiae may be widely associated with *V. carteri*, and likely have often been lost during host evolution.

## Background

The order *Rickettsiales* (*Alphaproteobacteria*), generally termed the rickettsiae, contains Gram-negative obligate intracellular bacteria [1], which are well-studied pathogens of mammals; are manipulators of host sexual reproduction [2]; and are candidate organisms for mitochondrial ancestors [3]. The family *Rickettsiaceae* of the *Rickettsiales* includes many agents causing tick-borne disease, and contains two genera, *Rickettsia* and *Orientia*, both of which are hosted principally by arthropod cells [1]. Bacteria of the *Rickettsiaceae* are also found as endosymbionts of non-arthropod organisms including leeches [4],[5], hydras [6], and ciliates [7–9]; the endosymbionts hosted by non-arthropods form a monophyletic group (termed the “hydra group”) within the family *Rickettsiaceae*, based on phylogenetic analyses of 16S ribosomal RNA (rRNA) genes [10]. Recently, endosymbiotic bacteria of the hydra group have been found in non-phagotrophic cells of the green algae *Carteria cerasiformis*, *Pleodorina japonica*, and *Volvox carteri* (Volvocales, Chlorophyceae)[11],[12] and marine green macroalgae (*Bryopsis* spp). (Ulvophyceae)[13]. Schrallhammer et al. [8] suggested a provisional species name “*Candidatus* Megaira polyxenophila” for endosymbionts of the hydra group harbored by cells of ciliates and volvocalean algae.

Infection or transmission of endosymbionts belonging to the *Rickettsiaceae* is transmitted from arthropods to vertebrates (reviewed in e.g.: [14]) and to land plants [15], although several studies have suggested that other routes of horizontal transmission may be in play [5],[9],[10]. Thus, the mode of transmission of rickettsial endosymbionts to non-phagotrophic cells of Volvocales is an interesting question. Of 10 strains of four closely related species of *Carteria*, the rickettsial endosymbiont was detected in only *C*. *cerasiformis* strain NIES-425 [11]. A similar situation was evident when *V*. *carteri* was studied; the endosymbiont was detected in only *V*. *carteri* f. *weismannia* strain UTEX 2180, among nine strains belonging to three forms (f. *weismannia*, f. *nagariensis*, and f. *kawasakiensis*) of this species [12]. These data suggest that the rickettsial endosymbiont may have been transmitted to the algal strains only very recently, after divergence of such strains.

*V*. *carteri* serves as a model organism for studies on multicellularity and the evolution of sexual reproduction [16],[17]. The nuclear genomic sequence of *V*. *carteri* f. *nagariensis* strain EVE has been determined recently [18]. Although no rickettsial endosymbiont was evident in this strain (based on performance of genomic PCR using the hydra group-specific primers, and 4′,6-diamidino-2-phenylindole [DAPI]-staining of algal cells [12]), we detected in preliminary work a sequence similar to that of rickettsial 16S rRNA genes in the nuclear genome of *V*. *carteri* f. *nagariensis* strain EVE. The presence of rickettsial gene-like sequences in the nuclear genome of this strain may indicate the occurrence of horizontal gene transfer (HGT) from a rickettsial bacterium to the ancestor of *V*. *carteri* f. *nagariensis* strain EVE.

The present study was undertaken to elucidate the origin of rickettsial gene-like sequences within the nuclear genome of *V*. *carteri* f. *nagariensis* strain EVR, compared with other 12 strains of *V*. *carteri*. Phylogenetic analyses of rickettsial gene and gene-like sequences from the endosymbionts, and the nuclear genomes of volvocalean algae, indicated that the rickettsial sequence of *V*. *carteri* f. *nagariensis* strain EVE was closely related to that of *P*. *japonica*, rather than that of *V*. *carteri* f. *weismannia* strain UTEX 2180.

## Results

### Sequences and synteny of rickettsial gene/gene-like sequences in nuclear genomes, and endosymbionts harbored by cells of the Volvocales

**Fig 1:**
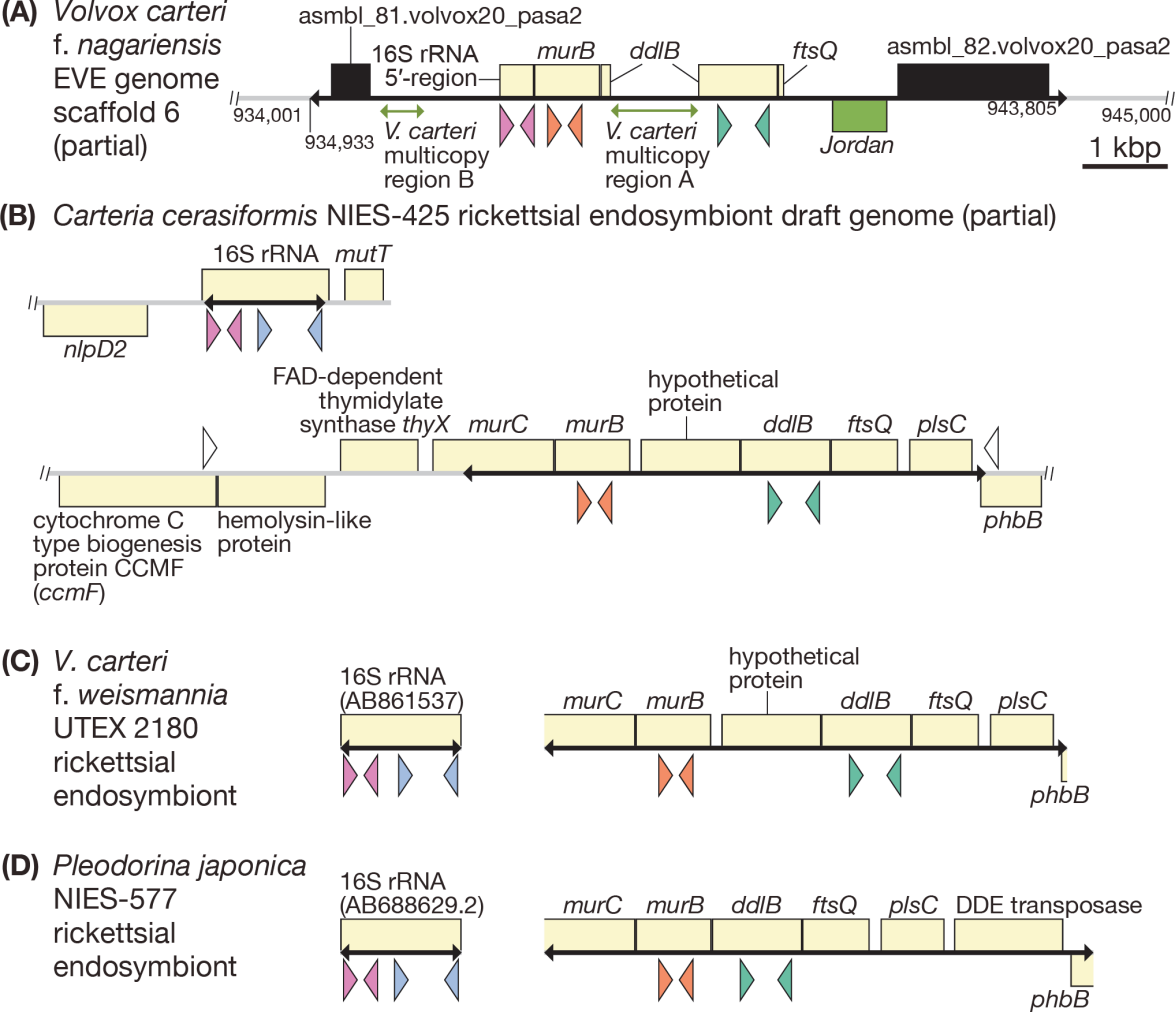
Rickettsial genes and gene-like sequences in the genomes of *Volvox carteri* and rickettsial possible endosymbionts. Schematic representations of arrangements/synteny of several rickettsial genes and gene-like sequences present in DNA of the nuclear genome of *V*. *carteri* (A) and in the genomes of rickettsial possible endosymbionts harbored by three volvocalean species (B-D). Coding DNA sequences (CDSs) and CDS-like regions are shown as boxes. Rickettsial CDSs/CDS-like regions are shown in pale yellow, the *V*. *carteri* transposon *Jordan*-like region in green and others in black. Placement of boxes above/below the line indicates gene direction (from left-to-right or right-to-left, respectively). Black double-headed arrows on the baseline indicate the regions sequenced in the present study. Colored triangles under boxes indicate the locations of primers used for semi-quantitative genomic PCR (16S rRNA gene 5′-region: magenta, 16S rRNA gene 3′-region: light blue, *murB*: orange, *ddlB*: green; Fig. 2E). For accession numbers of sequences used in this figure, see S3 Table. (A) Part of scaffold 6 of the *V*. *carteri* f. *nagariensis* strain EVE nuclear genome. (B) Part of the *Carteria cerasiformis* NIES-425 draft endosymbiont genome, including 16S rRNA (first line) and *murB*-*ftsQ* (second line). White triangles indicate primers used to amplify the sequencing templates (ccmF-R02 and phbB-F01; see Materials and Methods). (C) Part of the genome of a possible endosymbiont of *V*. *carteri* f. *weismannia* strain UTEX 2180, including *murB* and *ddlB* (right). The 16S rRNA gene of the endosymbiont [12] is also shown (left). (D) Part of the genome of a possible endosymbiont of *Pleodorina japonica* strain NIES-577, including *murB* and *ddlB* (right). The 16S rRNA gene [11] is also shown (left).

We performed a BLASTN search using long contiguous (contig) sequences (>5 kbp), from our ongoing genome data collection of the *Carteria cerasiformis* strain NIES-425 rickettsial endosymbiont, against the *Volvox carteri* f. *nagariensis* strain EVE nuclear genome. We found that the three rickettsial gene-like sequences with the highest E-values (S1 Table) were localized in a synteny region within scaffold 6 of the *V*. *carteri* f. *nagariensis* strain EVE nuclear genome (Fig. 1A). The three genes were a 16S rRNA gene, a gene encoding UDP-N-acetylenolpyruvylglucosamine reductase (*murB*), and a gene encoding D-alanine-D-alanine-ligase B (*ddlB*). In addition to these three rickettsial gene-like sequences, the synteny contained a short sequence similar to that of the cell division septal protein FtsQ gene (*ftsQ*; this was found upon additional BLASTN searching) and three *V*. *carteri*-specific sequences (see below) within a region approximately 9 kbp in length (Fig. 1A). In order to make sure that the synteny region is not contaminated and correctly assembled, this 9 kbp-region was confirmed via direct sequencing of genomic PCR products from total DNA of *V*. *carteri* f. *nagariensis* strain EVE (see Fig. 1A and Materials and Methods). No nucleotide difference was found between the ca. 9 kbp-region sequenced and the corresponding region of scaffold 6 of strain EVE genome.

**Fig 2:**
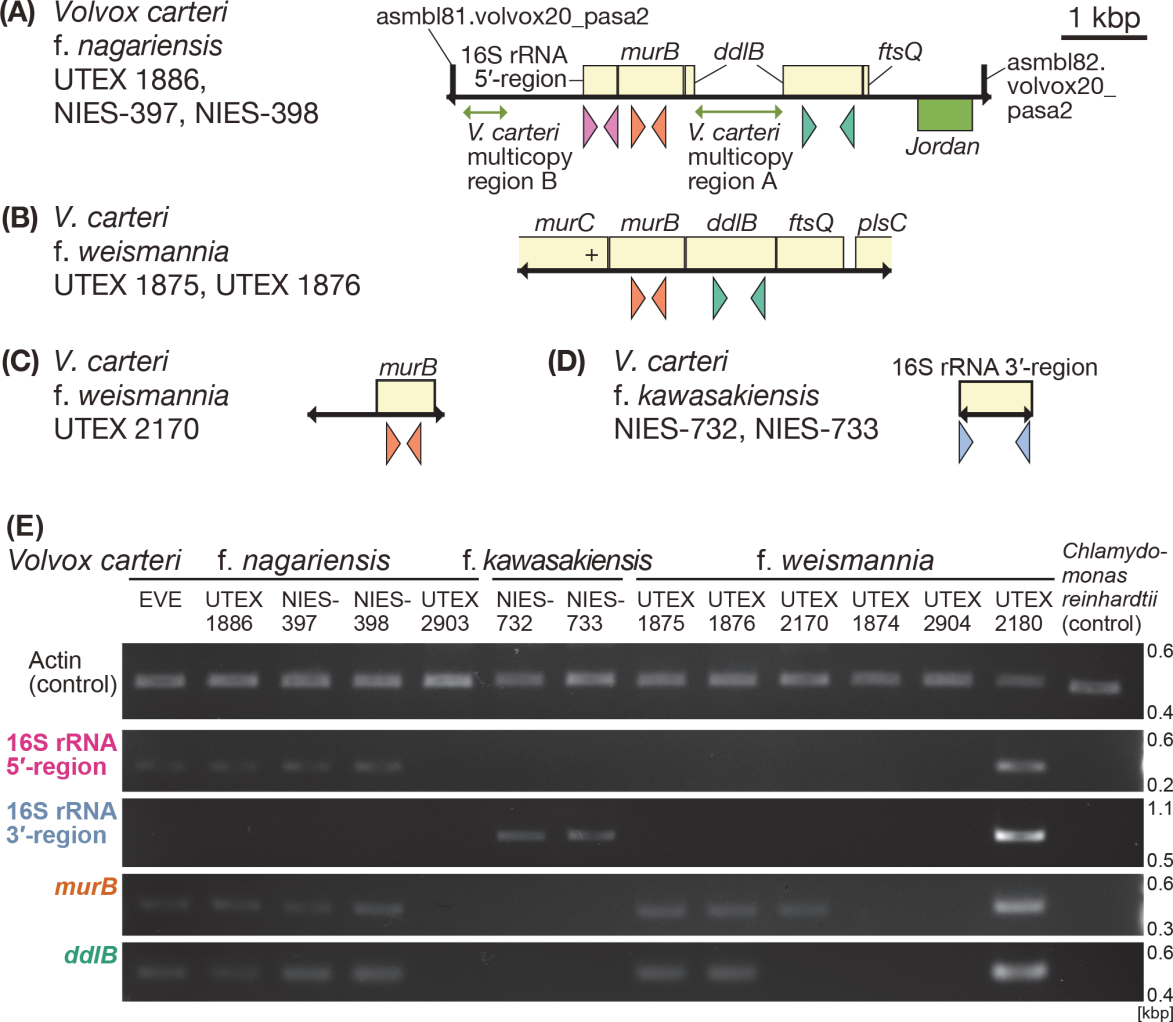
Rickettsial gene-like sequences possibly located in the nuclear genomes of *Volvox carteri* strains. Schematic representations of arrangements/synteny (A-D) in, and semi-quantitative genomic PCR data (E) from, several rickettsial gene-like sequences possibly located in the nuclear genomes of *V*. *carteri* strains. Coding DNA sequence (CDS)-like regions are shown as boxes. Rickettsial CDS-like regions are shown in pale yellow, the *V*. *carteri* transposon *Jordan*-like region in green and others in black. Placement of boxes above/below the line indicates the gene direction (from left-to-right or right-to-left, respectively). Black double-headed arrows on the baseline indicate the regions sequenced in the present study. Colored triangles under boxes indicate the primers used for semi-quantitative genomic PCR (16S rRNA gene 5′-region: magenta, 16S rRNA gene 3′-region: light blue, *murB*: orange, *ddlB*: green; Fig. 2E). For accession numbers of sequences used in this figure, see S3 Table. (A) Sequences including rickettsial CDS-like regions of *V*. *carteri* f. *nagariensis* strains UTEX 1886, NIES-397 and NIES-398. (B) Sequences including rickettsial gene homologs of *V*. *carteri* f. *weismannia* strains UTEX 1875 and UTEX 1876. Plus (+) indicates a frameshift deletion. (C) Sequence including rickettsial *murB*-like sequence of *V*. *carteri* f. *weismannia* strain UTEX 2170. (D) Sequences including rickettsial 16S rRNA gene-like sequences of *V*. *carteri* f. *kawasakiensis* NIES-732 and NIES-733. (E) Semi-quantitative genomic PCR of rickettsial genes and gene-like sequences. Each rickettsial gene-like sequence was amplified via genomic PCR using rickettsia-specific primer sets (see Materials and Methods). The positions of primer sets with reference to target positions are shown in both Fig. 1 and this Fig. 2. As a control, the actin gene was amplified. *Chlamydomonas reinhardtii* strain CC-503 was used as negative control.

The rickettsial 16S rRNA gene-like sequence within the synteny region of the *V*. *carteri* f. *nagariensis* strain EVE genome was 412 bp in length and corresponded to the 5′-region of the *C*. *cerasiformis* strain NIES-425 endosymbiont 16S rRNA gene (including the 5′ end). The 3′-region of the 16S rRNA gene-like sequence was truncated, and lay adjacent to a sequence similar to that of the rickettsial *murB* gene. This *murB*-like sequence was 768 bp in length, contained the 3′-end of the coding region, and exhibited no frameshift or premature stop codon, although the sequence seemed to lack a 5′-coding-region of 117 bp (including the start codon) when compared with *murB* of the *C*. *cerasiformis* strain NIES-425 endosymbiont. The sequence similar to that of rickettsial *ddlB* was interrupted by a 1,021-bp insertion (“*V*. *carteri* multicopy A” in Fig. 1A), but had both a start and a stop codon, and exhibited no frameshift or premature stop codon (the insertion was excluded from analysis). The insertion (*V*. *carteri* multicopy A) had no sequence similar to that of rickettsial genes, but had 103 DNA sequences similar to those distributed in the *V*. *carteri* f. *nagariensis* strain EVE nuclear genome (E-value = 0; nucleotide identity, 84–100%; a BLASTN search yielded these data). Thus, it appeared specific to *V*. *carteri*. The *ftsQ*-like sequence was short (75 bp), but exhibited high-level similarity (nucleotide identity: 88%) to the 5′-region (including the start codon) of *ftsQ* of the endosymbiont of *C*. *cerasiformis* strain NIES-425. A sequence resembling the 5′-region of the *V*. *carteri*-specific transposon *Jordan* [19] was also found (E value = 0; nucleotide identity 96%); the sequence was located near the *ftsQ*-like sequence, but lacked the 5′-inverted terminal repeat found in *Jordan* [19]. A DNA sequence of 516 bp located in the 5′-upstream region of the rickettsial 16S rRNA-like sequence (“*V*. *carteri* multicopy B” in Fig. 1A) may also be *V*. *carteri*-specific because it had 32 similar DNA sequences (E values = 0.0, nucleotide identity 95–98%) within the *V*. *carteri* f. *nagariensis* strain EVE nuclear genome (found using a BLASTN search). This region exhibited no similarity to the rickettsial genes or DNA inserted in the *ddlB*-like sequence. At both ends of the ca. 9 kbp-DNA region including rickettsial gene-like sequences, two regions (asmbl_81.volvox20_pasa2 and asmbl_82.volvox20_pasa2) that match with EST sequences are identified on Phytozome version 9.1 (http://www.phytozome.net) [20]. These regions were not similar to any sequences in our preliminary genome assembly database of the rickettsial endosymbiont of *C*. *cerasiformis* strain NIES-425, and their functions are unknown.

In the rickettsial endosymbiont genome of *C*. *cerasiformis* strain NIES-425, *murB*, *ddlB*, and *ftsQ* formed a synteny, but the 16S rRNA gene was separated from the three genes, being composed of two separate DNA sequences (Fig. 1B). One sequence was 1,422 bp long and encoded a 16S rRNA gene positioned between genes encoding nucleoside triphosphate pyrophosphohydrolase (*mutT*) and a M23 superfamily membrane-bound metallopeptidase (*nlpD2*). The other sequence was 6,230 bp long and encoded *murB*, *ddlB*, *ftsQ*, UDP-N-acetylmuramate-alanine ligase (*murC*), and three other proteins (Fig. 1B, black double-headed arrows on the baseline). In the endosymbiont genome of *C*. *cerasiformis* strain NIES-425, a coding DNA sequence (CDS) for a hypothetical protein was inserted between *murB* and *ddlB*.

Partial genomic DNA sequences including the *murC*, *murB*, *ddlB*, and *ftsQ* genes were obtained using total DNAs of rickettsial endosymbiont-containing cells of *V*. *carteri* f. *weismannia* strain UTEX 2180 (Fig. 1C) and *P*. *japonica* strain NIES-577 (Fig. 1D). These sequences were similar to that of the endosymbiont of *C*. *cerasiformis* strain NIES-425 in terms of gene arrangement. However, the *P*. *japonica* strain NIES-577 sequence differed from the other two sequences in that an additional CDS was lacking between *murB* and *ddlB*, and a CDS encoding a DDE transposase was located between DNA encoding 1-acyl-sn-glycerol-3-phosphate acyltransferase (*plsC*) and acetoacetyl-CoA reductase (*phbB*) (Fig. 1D).

### Rickettsial gene-like sequences in various *V*. *carteri* strains

Performance of genomic PCR using 16S rRNA primers specific to the hydra group, and DAPI-staining of algal cells from 13 strains of three forms of *V*. *carteri*, showed that *V*. *carteri* f. *weismannia* strain UTEX 2180 harbored a rickettsial endosymbiont, whereas the 12 other strains (including *V*. *carteri* f. *nagariensis* strain EVE) did not [12] (S1, S2 Figs.). Sequences similar to those of rickettsial genes were detected in nine endosymbiont-lacking strains of *V*. *carteri* via genomic PCR using specific primers (S2 Table) targeting endosymbiont genes and rickettsial gene-like sequences in the nuclear genome of *V*. *carter*i f. *nagariensis* strain EVE (Fig. 1). We sequenced the PCR products from all nine strains (Fig. 2E and S3 Fig.). The sequences were classified into four types: (A)-(D). Type A: *V*. *carteri* f. *nagariensis* strains UTEX 1886, NIES-397, and NIES-398 had sequences of 6,284–6,290 bp that were completely or almost identical (99–100% nucleotide identity) to part of scaffold 6 of the published *V*. *carteri* f. *nagariensis* strain EVE nuclear genome (Fig. 1A), encompassing sequences similar to those of rickettsial 16S rRNA, *murB*, *ddlB*, *ftsQ*, *V*. *carteri*-specific transposon *Jordan*, and two *V*. *carteri*-specific sequences (*V*. *carteri* multicopies A and B) (Fig. 2A); Type B: *V*. *carteri* f. *weismannia* strains UTEX 1875 and 1876 contained DNA sequences including five regions that were very similar to a sequence of *C*. *cerasiformis* strain NIES-425 (containing the *murC*, *murB*, *ddlB*, *ftsQ*, and *plsC* genes; 88–92% nucleotide identity; Fig. 2B). In these five regions, the sequences similar to *murB*, *ddlB*, and *ftsQ* exhibited intact open reading frames (ORFs), whereas the *murC*-like sequences had frameshift mutations in the 3′ regions. The gene arrangement of this DNA sequence was similar to that of *P*. *japonica* strain NIES-577, in that a CDS encoding a hypothetical protein, lying between the *murB* and *ddlB* genes/gene-like sequences, was lacking. Type C: A 678 bp sequence (only), similar to that of truncated rickettsial *murB*, was detected in *V*. *carteri* f. *weismannia* strain UTEX 2170 (Fig. 2C). This *murB*-like sequence lacked both the 5′ and 3′ ends, although no frameshift or premature stop codon was evident. Type D: In *V*. *carteri* f. *kawasakiensis* strains NIES-732 and NIES-733, no sequence exhibiting similarity to those encoding rickettsial *murB*, *ddlB*, or *ftsQ* was detected, but we found a rickettsial 16S rRNA gene-like sequence (Fig. 2D). This sequence was 882 bp long and corresponded to the 3′-region of the 16S rRNA gene from the endosymbiont of *C*. *cerasiformis* strain NIES-425 (Fig. 1B). However, sequence corresponding to the partial 16S rRNA gene-like sequence (the 5'-region) found in the nuclear genome of *V*. *carteri* f. *nagariensis* strains was lacking.

### Semi-quantitative PCR of rickettsial gene/gene-like sequences from *Volvox carteri* strains

To determine whether the rickettsial gene/gene-like sequences were present in the nuclear genome or in endosymbionts of the cytoplasm, we performed semi-quantitative PCR on total DNA of 13 strains of *V*. *carteri* (Fig. 2E). Of these *V*. *carteri* strains, the rickettsia-harboring *V*. *carteri* f. *weismannia* strain UTEX 2180 exhibited apparently higher amplification than did the other 12 strains lacking rickettsial endosymbionts, when primer sets targeting rickettsial gene and gene-like sequences were employed (Fig. 2E). This indicated that the numbers of rickettsial gene or gene-like sequence molecules targeted by PCR in the rickettsia-harborin*g* strain (*V*. *carteri* f. *weismannia* strain UTEX 2180) was greater than those of other cells lacking endosymbionts. Thus, high-level detection of *V*. *carteri* f. *weismannia* strain UTEX 2180 sequences may reflect the presence of many endosymbionts within host algal cells, and lower-level detection the presence of low copy-number sequences within the nuclear genome.

### Phylogenetic analysis of rickettsial gene-like sequences from *V*. *carteri*

**Fig 3:**
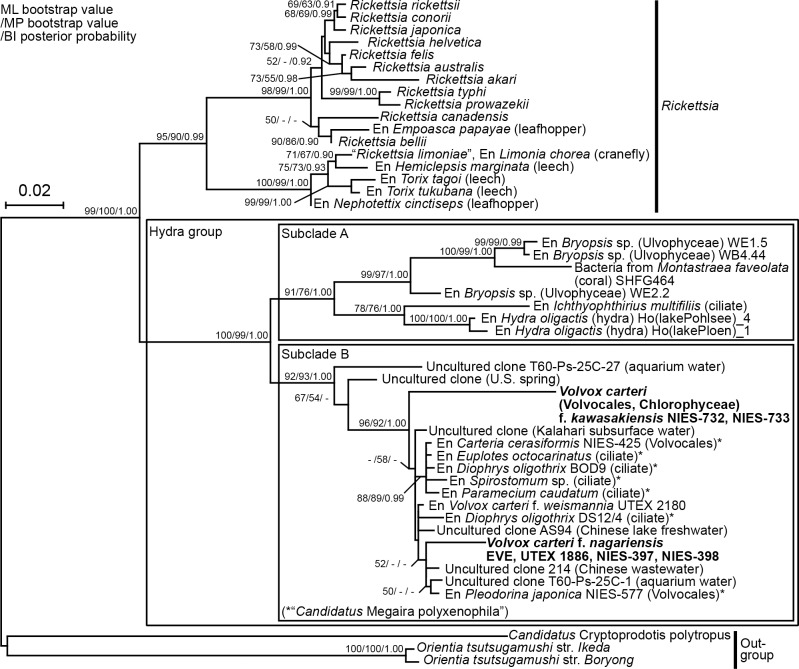
Phylogenetic positions of rickettsial 16S rRNA gene-like sequences in *Volvox carteri* nuclear genomes. The tree was inferred using the maximum-likelihood (ML) method based on 43 sequences and 1,403 nucleotides of the 16S rRNA genes from bacteria, endosymbionts (En) of eukaryotic hosts, and other environmental samples, of the family *Rickettsiaceae*, including rickettsial 16S rRNA gene-like sequences obtained from endosymbiont-lacking strains of *V*. *carteri* (bold). Bootstrap values (≥50%) for the ML and maximum parsimony analyses, and posterior probabilities (≥0.90) for Bayesian interference, are indicated at the respective nodes. The scale bar corresponds to 0.02 nucleotide substitutions per position. The hydra group and ‘*Candidatus* Megaira polyxenophila’ refer to the organisms studied by Weinert et al. [10] and Schrallhammer et al. [9], respectively.

In a phylogenetic tree constructed using rickettsial 16S rRNA gene and gene-like sequences, the family *Rickettsiaceae* was divided into two robust monophyletic groups, as shown previously [12]: the first group corresponded to “*Rickettsia*” [4],[5] that included certain endosymbionts of leeches, and the second the hydra group (Fig. 3). The second group was subdivided into two subclades A and B, as previously described [12] [supported by 76–93% bootstrap values upon maximum-likelihood (ML) and maximum parsimony (MP) analysis, and a 1.00 posterior probability (PP) by Bayesian inference (BI)]. Subclade A contained bacterial sequences derived from coral, endosymbionts hosted by *Hydra oligactis*, marine green macroalgae (*Bryopsis* spp.), and the freshwater ciliate *Ichthyophthirius multifiliis*. Subclade B included 16S rRNA gene-like sequences from six endosymbiont-lacking strains of *V*. *carteri* f. *kawasakiensis* and f. *nagariensis*, and those of 16S rRNA genes from endosymbionts of *C*. *cerasiformis* strain NIES-425, *P*. *japonica* strain NIES-577, and *V*. *carteri* f. *weismannia* strain UTEX2180 [11],[12]. These endosymbionts, and those hosted by ciliates (in subclade B), have been assigned to “*Candidatus* Megaira polyxenophila” [9]. Within subclade B, endosymbionts from *C*. *cerasiformis* strain NIES-425, and four ciliates, formed a robust monophyletic group (88–89% bootstrap values and a PP of 0.99). A weak bootstrap value (52%) upon ML analysis suggested that the endosymbiont of *P*. *japonica* strain NIES-577, and the 16S rRNA gene-like sequences from four strains of *V*. *carteri* f. *nagariensis*, were positioned into a small monophyletic group that did not include the endosymbiont of *V*. *carteri* f. *weismannia* strain UTEX 2180 or the gene-like sequence of *V*. *carteri* f. *kawasakiensis*.

**Fig 4:**
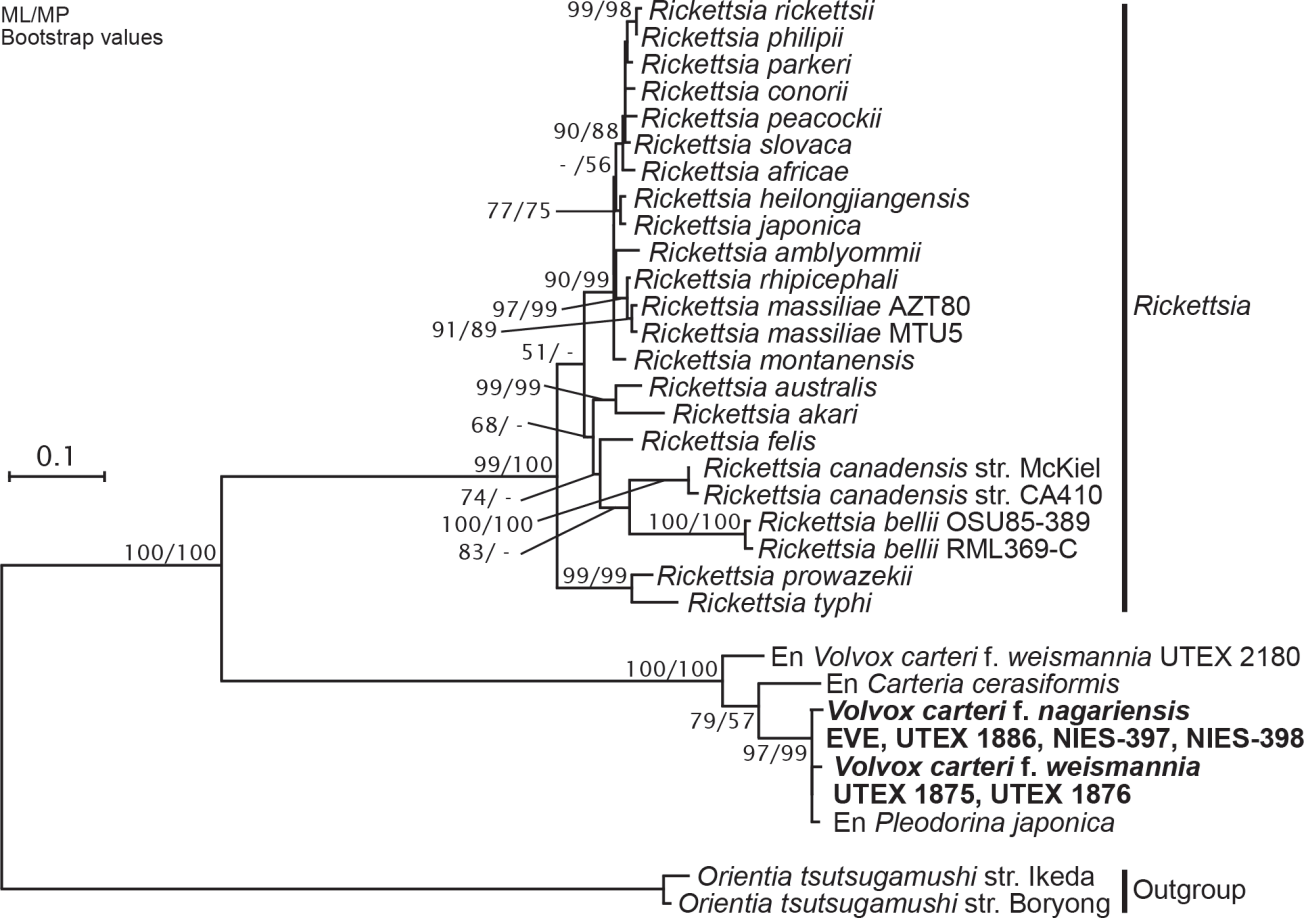
Phylogenetic positions of rickettsial *murB* and *ddlB* gene-like sequences from endosymbiont-lacking strains of *Volvox carteri*. The tree was inferred using the maximum-likelihood (ML) method based on 637 amino acid sites in translated and combined *murB* and *ddlB* gene/gene-like sequences from 30 operational taxonomic units of bacteria in the *Rickettsiaceae*, including possible endosymbionts (En) of algal hosts, and possible nuclear-encoded sequences from endosymbiont-lacking strains of *V*. *carteri* (bold). Bootstrap values (≥50%) for ML and maximum parsimony analyses are indicated at the respective nodes. The scale bar corresponds to 0.1 amino acid substitutions per position.

Phylogenetic analyses of *murB* and *ddlB* genes and gene-like sequences yielded essentially the same results (S4, S5 Figs.). Rickettsial gene-like sequences from endosymbiont-lacking strains of *V*. *carteri* f. *nagariensis* and f. *weismannia* formed a robust monophyletic group, combined with sequences from three endosymbiont-containing strains (*C*. *cerasiformis* strain NIES-425, *P*. *japonica* strain NIES-577, and *V*. *carteri* f. *weismannia* strain UTEX 2180); the bootstrap values were 99–100% by both ML and MP analysis. In this monophyletic group, *V*. *carteri* f. *weismannia* strain UTEX 2180 and *Carteria cerasiformis* strain NIES-425 were (respectively) primarily and secondarily basal to all others members. Combined amino acid data from these two gene/gene-like sequences (Fig. 4) showed that rickettsia-lacking strains of *V*. *carteri* f. *nagariensis* and f. *weismannia*, and the endosymbiont-containing *P*. *japonica* strain NIES-577, formed a robust small clade (with 97–99% bootstrap values upon ML and MP analyses), which did not include *V*. *carteri* f. *weismannia* strain UTEX 2180 or *C*. *cerasiformis* strain NIES-425.

### Phylogenetic relationships among various strains of three forms of *Volvox carteri* based on ITS-2 sequences of nuclear rDNA

**Fig 5:**
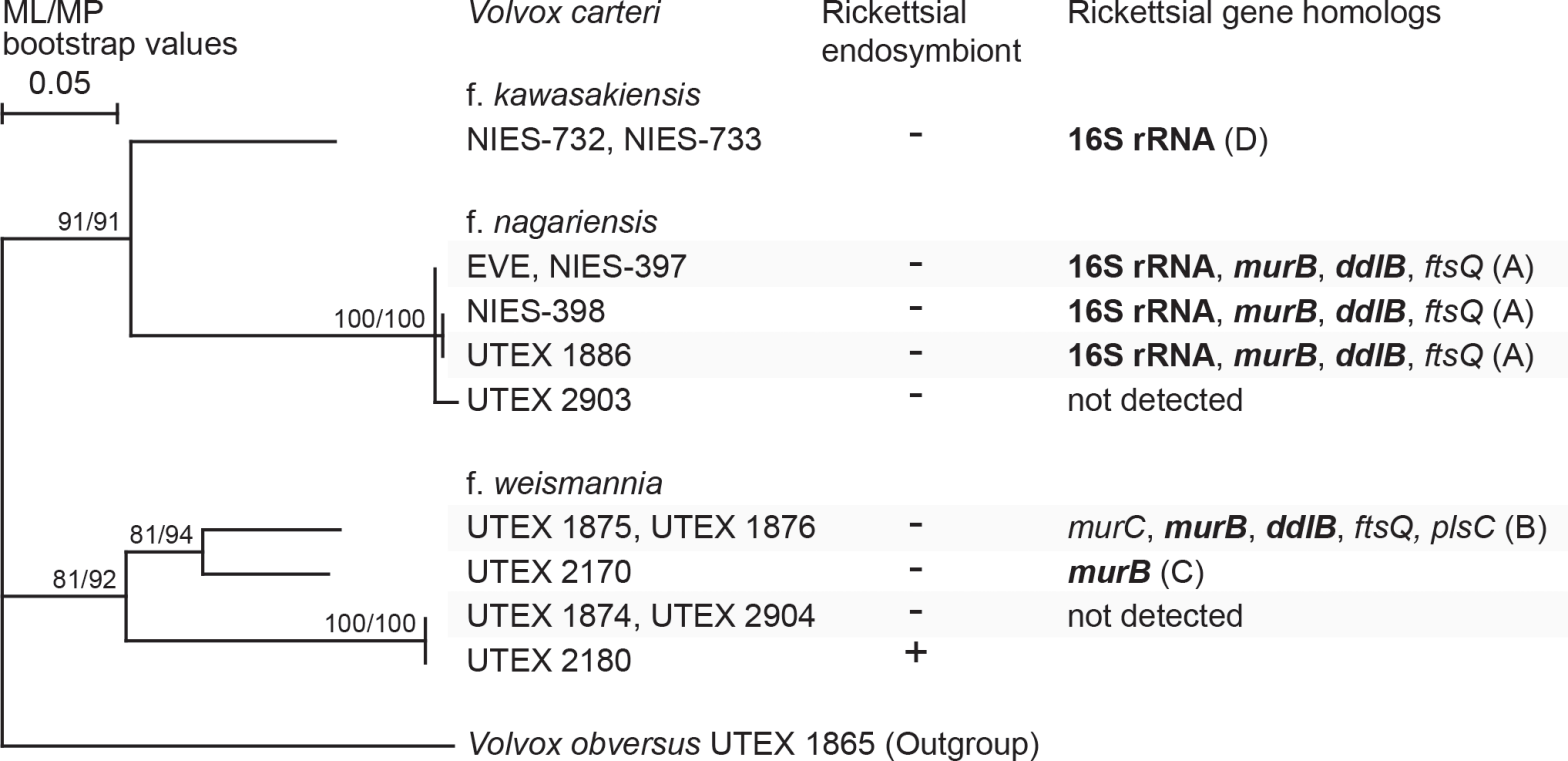
Phylogenetic relationships among 13 strains of three forms of *Volvox carteri*. The tree was inferred using the maximum-likelihood (ML) method based on alignment of 471 nucleotide sites in the internal transcribed spacer 2 sequences of 10 operational taxonomic units of *V*. *carteri* strains and *V*. *obversus* strain UTEX 1865 (the outgroup). Bootstrap values (50% or more) for the ML and maximum parsimony analyses are indicated at the respective nodes. The scale bar shows 0.05 nucleotide substitutions per position. The presence (+) or absence (-) of rickettsial endosymbionts based on the data of Kawafune et al. [12], and those of the present study (S1, S2 Figs.), are shown in the central column. Possible nuclear-encoded, rickettsial gene homologs detected in the present study (Fig. 2E and S3 Fig.) are shown in the column on the right. The gene names shown in bold were used in phylogenetic analyses (Figs. 3, 4 and S4, S5 Figs.). The letters A-D following gene names correspond to the forms of genetic composition shown in Fig. 2. No rickettsial gene-like sequences were detected (in the present study) in strains UTEX 2903, UTEX 1874 or UTEX 2904.

Coleman [21] derived phylogenetic relationships among three forms of *V*. *carteri* based on internal transcribed spacer (ITS) sequences of nuclear ribosomal DNA (rDNA) (ITS-1, the 5.8S rRNA gene, and ITS-2), using only five strains. Thus, we constructed a phylogenetic tree based on nuclear rDNA ITS-2 sequences from 13 strains of three forms of *V*. *carteri* (S3 Table). As reported by Coleman [21], strains of f. *nagariensis* and f. *kawasakiensis* formed a robust monophyletic group (91% bootstrap values) to which strains of f. *weismannia* were sister (Fig. 5). Within f. *weismannia*, two sister clades were well-resolved (with 81–92% bootstrap values); one contained the rickettsia-lacking strains UTEX 1875, UTEX 1876, and UTEX 2170, in which (at least) rickettsial *murB* gene homologs were detected in the present study; and the other the rickettsia-lacking strains UTEX 1874 and UTEX 2904, and the rickettsia-containing strain UTEX 2180.

## Discussion

The present phylogenetic analyses (Figs. 3, 4 and S4, S5 Figs.) indicated that the rickettsial sequences derived from nine strains of *V*. *carteri* apparently belong to the hydra group. However, our PCR experiment of these nine strains using the hydra group-specific primers [12] (S2 Fig.) demonstrated the absence of the whole 16S rRNA gene that is essential for the living endosymbiotic bacteria. In addition, the DAPI-staining of the algal cells in these nine strains [12] (S2 Fig.) clearly shows the absence of bacterial cells within the host algal cells. Thus, the rickettsial gene-like sequences detected in the nine strains of *V*. *carteri* cannot be actual genes of endosymbionts. It should also be noted that, even if the 16S rRNA gene-specific primers and light microscopic examination by the DAPI-staining could have missed rickettsial endosymbiotic variants that do not belong to the hydra group, the sequences positioned within the possible hydra group (Figs. 3, 4 and S4, S5 Figs.) should not originate from such missing endosymbionts. In addition, our semi-quantitative PCR results of the 12 endosymbiont-lacking strains of *V*. *carteri* (Fig. 2E) indicated that the degree of amplification of their sequences is consistent with that of the low copy DNA such as that in the nuclear genome. Thus, the rickettsial gene-like sequences from the endosymbiont-lacking *V*. *carteri* strains are considered to be coded in the nuclear genome of the host cells. It could also be speculated that the rickettsial gene-like sequences in rickettsial endosymbiont-lacking strains of *V*. *carteri* might have been transferred to other endosymbionts still remaining in the host. However, this scenario seems very unlikely because we experimentally confirmed the 6–9 kbp-DNA sequences encompassing both rickettsial and *V*. *carteri*-specific sequences (Figs. 1A, 2A).

The rickettsial 16S rRNA and/or *murB* gene homologs were found in various *V*. *carteri* strains lacking rickettsial endosymbionts in the cytoplasm (Figs. 1, 2A-2D). Upon phylogenetic analysis, the nuclear-encoded 16S rRNA gene homologs of *V*. *carteri* f. *nagariensis* (strains EVE, UTEX 1886, NIES-397, and NIES-398) and f. *kawasakiensis* (strains NIES-732 and NIES-733) belonged to subclade B within the hydra group, as did the sequences of rickettsial endosymbionts of *V*. *carteri* f. *weismannia* strain UTEX 2180, *P*. *japonica* strain NIES-577, and *C*. *cerasiformis* strain NIES-425 (Fig. 3). Although rickettsial 16S rRNA gene-like sequences were not evident in three endosymbiont-lacking strains of *V*. *carteri* f. *weismannia* (UTEX 1875, UTEX 1876, and UTEX 2170), their *murB* and *ddlB* (except for UTEX 2170) homologs formed a monophyletic group in which endosymbiotic genes of *V*. *carteri* f. *weismannia* strain UTEX 2180 and *C*. *cerasiformis* strain NIES-425 were basally positioned (Fig. 4 and S4, S5 Figs.). Therefore, rickettsial gene homologs in the nuclear genome of *V*. *carteri* may have originated from rickettsial bacteria of subclade B via horizontal gene transfer. As the *Rickettsiales* are obligate intracellular bacteria, and as their hosts (green algal cells) are not phagotrophic, the donor rickettsial organisms may have been harbored by ancestral cells of rickettsial endosymbiont-lacking strains of *V*. *carteri*. Thus, endosymbiotic gene transfer (EGT) may have been used to transfer rickettsial gene-like sequences to the host nuclear genome. However, the donor rickettsial endosymbionts of *V*. *carteri* strains have apparently been subsequently lost.

All of *murC*, *murB*, *ddlB*, and *ftsQ* are component of the dcw (division and cell wall) cluster [22]. Some genes of dcw cluster have previously been shown to transfer from endosymbiotic bacteria to host eukaryote genomes; e.g., that of an adzuki bean beetle (a gene encoding cell division protein FtsZ [*ftsZ*]) [23]; that of a rotifer (*ddl*) [24]; and that of *Trichoplax adhaerens* (UDP-N-acetylglucosamine enoylpyruvyl transferase = *murA*) [25]. In the *T*. *adhaerens* model, it was suggested that the transferred *murA* gene was expressed, and limited the growth of endosymbionts of the family *Midichloriaceae* (*Rickettsiales*) [25]. In this study, however, although expression of rickettsial gene-like sequences in the nuclear genome of *V*. *carteri* f. *nagariensis* strains was not examined, most nuclear-encoded rickettsial gene-like sequences were incomplete when compared with the CDS of rickettsial genes of *C*. *cerasiformis* strain NIES-425 and *V*. *carteri* f. *weismannia* strain UTEX 2180 endosymbionts. Thus, such nuclear-encoded sequences may be non-functional in cells of *V*. *carteri* f. *nagariensis* strains. In addition, rickettsial 16S rRNA gene-like sequences in the nuclear genomes of *V*. *carteri* f. *nagariensis* strains lack sequences corresponding to the 3′ ends, and should be non-functional. Similarly, the genome of *Trichonympha agilis*, an eukaryotic symbiont in the termite gut, contains 16S rRNA pseudogenes that were likely transferred from bacterial endosymbionts, but have large deletions. It remains unknown whether such pseudogenes have a certain function as non-cording DNA or not [26].

Although the statistical support was weak (52% upon ML analysis), our phylogenetic analysis of 16S rRNA gene/gene-like sequences suggested that gene-like sequences in rickettsial endosymbiont-lacking strains of *V*. *carteri* f. *nagariensis* (EVE, UTEX 1886, NIES-397, and NIES-398) were closely related to that of the endosymbiont *P*. *japonica* strain NIES-577, rather than those of the endosymbionts of *V*. *carteri* f. *weismannia* strain UTEX 2180 or *C*. *cerasiformis* strain NIES-425 (Fig. 3). The close relationship between rickettsial genes/gene-like sequences of *V*. *carteri* f. *nagariensis* and the endosymbiont of *P*. *japonica* strain NIES-577 was robustly supported by phylogenetic analyses (Fig. 4 and S4, S5 Figs.) of *murB* and *ddlB* genes/gene-like sequences. In addition, the synteny of *murB* and *ddlB* in *P*. *japonica* strain NIES-577 was similar to that of *V*. *carteri* f. *nagariensis* gene-like sequences, lacking a CDS for a hypothetical protein encoded between *murB* and *ddlB* in endosymbionts of *V*. *carteri* f. *weismannia* strain UTEX 2180 and *C*. *cerasiformis* strain NIES-425 (Figs. 1, 2A). Thus, rickettsial gene-like sequences in the nuclear genome of *V*. *carteri* f. *nagariensis* may have been transmitted from an endosymbiont closely related to that of *P*. *japonica* strain NIES-577. It appears that such sequences were not derived directly from an endosymbiont closely related to that of *V*. *carteri* f. *weismannia* strain UTEX 2180. A similar EGT event featuring an endosymbiont closely related to that of the *P*. *japonica* strain NIES-577 may explain the origin of rickettsial gene-like sequences in three endosymbiont-lacking strains of *V*. *carteri* f. *weismannia* (Fig. 2B, C). *MurB* and *ddlB* gene/gene-like sequences from *V*. *carteri* f. *weismannia* strains, *V*. *carteri* f. *nagariensi*s strains, and *P*. *japonica* strain NIES-577 formed a small clade distinct from those of *V*. *carteri* f. *weismannia* strain UTEX 2180 (Fig. 4 and S4, S5 Figs.). However, the phylogenetic positions of rickettsial 16S rRNA gene-like sequences from *V*. *carteri* f. *kawasakiensis* strains NIES-732 and NIES-733 remain ambiguous because the sequences are short and no other rickettsial gene-like sequences were detected in these strains (Fig. 3).

*MurB* and *ddlB* gene/gene-like sequences from endosymbiont-lacking strains of *V*. *carteri* f. *nagariensis* and f. *weismannia* were closely related to those of the endosymbiont (or rickettsial gene-like sequences) of *P*. *japonica* NIES-577 (Fig. 4 and S4, S5 Figs.). However, these two forms of *V*. *carteri* were robustly separated upon phylogenetic analysis (Fig. 5). Thus, rickettsial gene-like sequences may have been independently transmitted to ancestors of the two forms of *V*. *carteri*, from endosymbiont(s) containing a sequence closely related to that of *P*. *japonica* strain NIES-577. Alternatively, such transmission may have occurred only once, in a common ancestor of the three forms of *V*. *carteri*. A similar EGT event occurring during historical endosymbiosis has been reported in an aphid genome; genes and pseudogenes were transferred from not only the primary endosymbiont *Buchnera aphidicola* (*Gammaproteobacteria*), but also from organisms related to *Wolbachia* spp. (*Anaplasmataceae*, *Rickettsiales*) and *Orientia tsutsugamushi*, neither of which exist in aphid cells [27],[28]. However, the EGT event that occurred in *V*. *carteri* remains unique, in that two different but closely related endosymbionts may have invaded closely related hosts. Thus, host specificity may be not high among endosymbionts within subclade B (Fig. 3) of the *Rickettsiaceae*.

Of the lineages of *V*. *carteri* f. *weismannia*, only UTEX 2180 contains the rickettsial endosymbiont. Nuclear rDNA ITS-2 sequences from UTEX 2180, UTEX 1874, and UTEX 2904 of *V*. *carteri* f. *weismannia* were almost identical (only two nucleotide deletions were noted). Thus, transmission of the rickettsial endosymbiont to UTEX 2180 may have been very recent, occurring after divergence of the three strains. Alternatively, transmission might have taken place prior to divergence of the three strains, with subsequent loss of endosymbionts in UTEX 1874 and UTEX 2904. Support for the latter scenario is afforded by the frequent loss of rickettsial endosymbionts in *V*. *carteri*. In our present study, we found that rickettsial gene-like EGT sequences were widely distributed in endosymbiont-lacking strains of *V*. *carter*i. Thus, loss of rickettsial endosymbionts may have been very common during evolution of *V*. *carteri*.

*V*. *carteri* f. *weismannia* strain UTEX 2180 and other hosts of rickettsial endosymbionts in the Volvocales may have acquired rickettsial EGT from present and past endosymbionts and may contain rickettsial gene-like EGT sequences in their nuclear genomes. Both rickettsial genes and gene-like EGT sequences are potentially amplified by PCR performed on total DNA of such strains. In the present study, rickettsial 16S rRNA sequences amplified from *V*. *carteri* f. *weismannia* strain UTEX 2180 and *P*. *japonica* strain NIES-577 (Fig. 1C-1D) came from the rickettsial endosymbionts, as previously confirmed by fluorescence *in situ* hybridization [11],[12]. The other rickettsial gene homologs (e.g., *murB*, *ddlB*) of *V*. *carteri* f. *weismannia* strain UTEX 2180 (Fig. 1C) are also thought to come from endosymbionts, as revealed by semi-quantitative PCR (Fig. 2E). However, we could not detect possible nuclear-encoded rickettsial gene-like sequences by direct sequencing of this strain, possibly because we studied only a limited number of genes employing selective primer sets. We speculate that the presence of high numbers of rickettsial endosymbiont cells may inhibit amplification of low copy-number EGT sequences in the nuclear genomes of host cells. It is impossible to distinguish between sequences derived from existing rickettsial endosymbionts and nuclear genomic sequences when the sizes of amplified fragments are near-identical.

Of *V*. *carteri* strains lacking rickettsial endosymbionts, no amplification of rickettsial gene-like sequences was observed in *V*. *carteri* f. *nagariensis* strain UTEX 2903 and f. *weismannia* strains UTEX 1874 and UTEX 2904 (S3 Fig.). Nevertheless, it remains possible that the genomes of these strains harbor other EGT sequences derived from past rickettsial endosymbionts. Whole-genome analyses of these *V*. *carteri* strains may reveal other EGT sequences.

## Conclusions

We showed that possible EGT-derived *Rickettsiaceae* gene-like sequences were present in various *V*. *carteri* strains that currently lack rickettsial endosymbionts. Comparison of synteny regions (Figs. 1, 2) and phylogenetic analyses of such gene-like sequences (Figs. 3, 4) revealed that at least two different rickettsial organisms invaded the *V*. *carteri* lineage; of which one was a direct ancestor of the endosymbiont of *V*. *carteri* f. *weismannia* strain UTEX 2180 and the other was closely related to the endosymbiont or rickettsial gene-like sequences of *P*. *japonica*. The latter rickettsial bacterium may have invaded ancestral cells of *V*. *carteri* f. *nagariensis* and f. *weismannia* strains, contributing to establishment of EGT nuclear genes, but has subsequently disappeared. As rickettsial endosymbionts harbored by green algal cells of the Volvocales are closely related to those of ciliates (Fig. 3), transmission of the rickettsial endosymbionts of subclade B may be based on ingestion of algal cells by ciliates, as suggested previously [8], implying that invasion and rickettsial gene transfer occurred several times in other species of Volvocales. In addition, endosymbionts may have been frequently lost, because rickettsial gene-like sequences are present in various endosymbiont-lacking strains of *V*. *carteri*, and because endosymbiont distribution is sporadic in the phylogenetic trees of *V*. *carteri* (Fig. 5) and *Carteria* [11]. A more detailed survey using further genomic information will reveal the details of the relationships between rickettsiae and cells of the Volvocales.

## Materials and Methods

### Culture, DNA extraction, and sequencing of the internal transcribed spacer (ITS) region

The strains of *V*. *carteri*, *V*. *obversus*, *C*. *cerasiformis* and *P*. *japonica* used in the present study (S3 Table) were supplied by the Culture Collection of Algae at the University of Texas at Austin (UTEX, USA; http://www.utex.org/) and the Microbial Culture Collection at the National Institute for Environmental Studies (NIES, Japan) [29]. *Volvox* cultures were sterilized and grown as described previously [12]. *C*. *cerasiformis* and *P*. *japonica* cultures were grown as described in Kawafune et al. [11]. *Chlamydomonas reinhardtii* strain CC-503 (*cw92* mt+) was supplied from Chlamydomonas Resource Center (http://chlamycollection.org) and was grown as described previously [12] except that TAP medium [30] was used. Total DNA was extracted as described by Kawafune et al. [11]. The presence or absence of rickettsial endosymbionts in nine *V*. *carteri* strains was established previously [12]. The *V*. *carteri* strains UTEX 1874, UTEX 1886, UTEX 2903, and UTEX 2904 were subjected to DAPI staining and genomic PCR to determine the presence or absence of rickettsial endosymbionts, as described previously [12]. The ITS regions of nuclear rDNA sequences of *V*. *carteri* strains and *V*. *obversus* UTEX 1865 (S3 Table) were identified as described in Setohigashi et al. [31], except for those of *V*. *carteri* f. *weismannia* strain UTEX 1875 and UTEX 1876, which are publicably available [32].

### BLAST-based screening of the *V*. *carteri* genome for transferred rickettsial genes

A BLASTN search [33] was performed on the *V*. *carteri* f. *nagariensis* EVE genome data (version 2, 8x, not masked) [18] on Phytozome version 9.1 (http://www.phytozome.net) [20] using 81 contigs (>5 kb) derived from our preliminary genome assembly database of the rickettsial endosymbiont hosted by *C*. *cerasiformis* strain NIES-425 (acquired as part of the Plant Global Education Project of the Nara Institute of Science and Technology) as queries. High-scoring segment pairs (HSPs) with E-values ≤1.0e-50 were annotated (S1 Table) using BLASTN and BLASTX searches [33] against nucleotide data and non-redundant protein sequences lodged in the National Center for Biotechnology Information database (NCBI, http://www.ncbi.nlm.nih.gov). Sequences associated with the three HSPs (similar to the rickettsial 16S rRNA gene, *murB*, and *ddlB*) that afforded the highest scores and E-values, were concentrated around base no. 940,000 of scaffold 6 on the *V*. *carteri* EVE genome. Additional BLASTN searching (using bases 935,001–945,000 of scaffold 6 as the query) against the 81 contigs of the *C*. *cerasiformis* NIES-425 rickettsial endosymbiont genome found one further short HSP (similar to rickettsial *ftsQ*, S1 Table); this HSP was also annotated as described above.

### Sequencing of rickettsial gene/gene-like sequences

To determine genomic sequences (including those of *murB*, *ddlB*, and *ftsQ* of rickettsial endosymbionts), PCR primers ccmF-R02 and phbB-F01 (S2 Table) were designed based on preliminary genomic information on the *C*. *cerasiformis* NIES-425 endosymbiont. Using these primers, ca. 10-kbp segments lying between the gene encoding cytochrome C type biogenesis protein CCMF (*ccmF*) and *phbB* were amplified from total DNAs of *C*. *cerasiformis* strain NIES-425, *P*. *japonica* strain NIES-577, and *V*. *carteri* f. *weismannia* strain UTEX 2180, via PCR (35 cycles of 98°C 10s, 60°C 15s and 68°C 5 min) with ca. 400 pg/μL DNA in 20 μL PCR solution, using Tks Gflex DNA Polymerase (Takara Bio Inc., Otsu, Japan).

In order to make sure the sequence of scaffold 6 of *V*. *carteri* f. *nagariensis* EVE genome, the sequence between bases 934,933–943,805 of scaffold 6 was amplified from total DNA of that strain via PCR using a *TaKaRa LA Taq* with GC I buffer (Takara Bio Inc.) and specific primers (eveFZ, eveRJ, eveFX, eveRD, eveFV, eveRT, asmb81_F4, asmb82_R2; S2 Table) with ca. 150 pg/μL DNA in 20 μL PCR solution, for 35 cycles of 94°C 20s, 53–55°C 30s and 72°C 2–5 min, followed by 72°C for 7 minutes. Each PCR product was purified using an illustra GFX PCR DNA and Gel Band Purification Kit (GE Healthcare UK Ltd., Buckinghamshire, UK) and directly sequenced on an ABI PRISM 3130xl Genetic Analyzer (ABI Life Technologies, Carlsbad, CA) using a BigDye Terminator Cycle Sequencing Ready Reaction Kit version 3.1 (Life Technologies) and internal sequencing primers (S2 Table).

For sequencing the rickettsial gene-like sequence of other *V*. *carteri* strains, genomic PCR was performed using various combinations of the rickettsial gene and gene-like sequence-specific primers (S2 Table). These primers were appropriately designed by reference to such sequences, as were primers amplifying previously determined 16S rRNA gene sequences of rickettsial endosymbionts (accession numbers: AB688628, AB688629, and AB861537) [11],[12]. PCR was performed with ca. 6–400 pg/μL DNA in 20 μL PCR solution, with 35 cycles of 94°C 20s, 53–57°C 30s and 72°C 2–5 min, followed by 72°C for 7 minutes. PCR products were directly sequenced as described above. In order to determine sequences around the *murB*-like sequence, vectorette PCR was also performed as described in Ko et al. [34] on the total DNA of *V*. *carteri* f. *weismannia* strain UTEX 2170, using *TaKaRa LA Taq* with GC I Buffer and the restriction enzymes *EcoR*I (TOYOBO Co., Ltd., Osaka, Japan) and *Apo*I (New England Biolabs, Ipswich, MA). PCR products were directly sequenced as described above.

### Detection of rickettsial gene and gene-like sequence by genomic PCR and semi-quantitative PCR

For confirming the detection of the rickettsial gene and gene-like sequence, genomic PCR was re-performed on total DNAs of *V*. *carteri* strains using the rickettsia-specific primer sets (16S rRNA 5′-region: eveFC and eveRD; 16S rRNA 3′-region: enFN and enRG; *murB*: murB-FP and murB-RK2; *ddlB*: ddlB-FQ and ddlB-RH2; 18S rRNA gene as positive control: 18S-FA and 18S-RD; see S2 Table), with 35 cycles of 94°C 20s, 55°C 30s and 72°C 40 sec, followed by 72°C for 7 minutes. An initial DNA concentration of PCR solution was ca. 2–3 pg/μL in 20 μL PCR solution. Total DNA of *Chlamydomonas reinhardtii* cc-503 was used as negative control; in the *Chlamydomonas reinhardtii* genome data (version 5.5, not masked) on Phytozome, no sequences similar to the rickettsial 16S rRNA gene, *murB*, *ddlB* and *ftsQ* were detected by a BLASTN search (E-values≤1.0e-50) using sequences of the rickettsial endosymbiont of *C*. *cerasiformis* strain NIES-425 as queries.

Semi-quantitative genomic PCR was also performed on total DNAs of *V*. *carteri* strains and *Chlamydomonas reinhardtii* CC-503 (as negative control), using *TaKaRa LA Taq* with GC Buffer I, and the same specific primer sets as the genomic PCR described above, with 27 cycles of 94°C 20s, 55°C 30s and 72°C 40 sec, followed by 72°C for 7 minutes and with a modification that the actin gene was amplified as a control (primers ONact1 and ONact2 for *V*. *carteri* and ONact1_CR and CR_IDA5_R3 for *Chlamydomonas reinhardtii* were used; see S2 Table) instead of 18S rRNA gene. An initial DNA concentration of PCR solution was ca. 2–3 pg/μL in 20 μL PCR solution.

### Phylogenetic analysis

The 16S rRNA gene-like sequences of *V*. *carteri* were aligned using ARB software [35] with a data matrix that has been described previously [12], modified by addition of the following operational taxonomic units (OTUs): an endosymbiont of *Euplotes octocarinatus* (accession number: FR823004); an endosymbiont of *Spirostomum* sp. (FR822998); an endosymbiont of *Paramecium caudatum* (FR822997); and an endosymbiont of *Diophrys oligothrix* DS12/4 (FR823001). Alignment was corrected manually (S1 File; also available from TreeBASE [http://treebase.org/treebase-web/home.html; study ID: 16773]) by reference to secondary structure.

The *murB* and *ddlB* gene-like sequences of *V*. *carteri* were manually aligned with the *murB* and *ddlB* nucleotide sequences of bacteria belonging to the family *Rickettsiaceae*, including those of the preliminary genomic sequence of the *C*. *cerasiformis* strain NIES-425 endosymbiont and sequences obtained from total DNAs of *P*. *japonica* strain NIES-577 and *V*. *carteri* f. *weismannia* strain UTEX 2180 (S4 Table). These nucleotide sequences were next converted to translated amino acid sequences (S1 File; also available from TreeBASE [study ID: 16773]).

Phylogenetic analyses of 16S rRNA gene/gene-like sequences, translated *murB*, translated *ddl*, and combined [translated *murB* and *ddlB*] genes/gene-like sequences, were performed using both ML and MP methods, employing PhyML 3.0 [36] and PAUP 4.0b10 [37], respectively, with 1,000 bootstrap replications. In addition, 16S rRNA genes/gene-like sequences were subjected to BI testing using MrBayes 3.2 [38]. For both ML analyses and BI, the GTR+gamma+I model was selected by jModelTest 2 [39],[40] to analyze the data matrix of 16S rRNA genes/gene-like sequences; and the CpREV+gamma, JTT+gamma+I+F, and JTT+gamma+I+F models were selected by ProtTest3 [39],[41] to analyze the the data matrixes of translated *murB*, *ddlB*, and combined genes/gene-like sequences, respectively.

The nuclear rDNA ITS-2 sequences of *V*. *carteri* strains and *V*. *obversus* strain UTEX 1865 were aligned based on secondary structures, predicted using the RNA Folding Form on the mFold Web Server (http://mfold.rna.albany.edu/?q=mfold) [42]; revised based on the data of previous studies [43],[44]; and drawn using VARNA 3.9 [45] (S2 File). Phylogenetic analyses of aligned sequences (S1 File; also available from TreeBASE [study ID: 16773]) were performed as described by Nozaki et al. [46].

## Supporting Information

S1 Fig Somatic cells of five *Volvox carteri* strains stained with DAPI. Vertical panels show the same cells shown at the same magnification, composed of epifluorescence images (A-E) and Nomarski differential interference images (F-J). The arrow, ‘n’ and ‘p’ indicate the chloroplast nucleoid, host cell nuclei and pyrenoid respectively. Scale bar = 10 μm. Any bacteria-like rod-shaped bodies were not observed in the cells of f. *weismannia* strain UTEX 1874 (A, F), f. *nagariensis* strain UTEX 1886 (B, G), f. *nagariensis* strain UTEX 2903 (C, H) and f. *weismannia* strain UTEX 2904 (D, I). On the other hand, the bacterial endosymbionts (arrowheads) were observed in the cell of f. *weismannia* strain UTEX 2180 (E, J) as rod-shaped fluorescent bodies as observed previously [12].
(TIF)

S2 Fig Detection of 16S rRNA gene of the *Rickettsiaceae* in *Volvox carteri* strains by genomic PCR. PCR amplification using the forward primer eveFC and reverse primer enRB (specific 16S rRNA primers specific to the bacteria belonging to the hydra group, see S2 Table) corresponds the presence or absence of rickettsial endosymbionts. EVE (lacking rickettsial endosymbiont) and UTEX 2180 (having rickettsial endosymbiont) are shown as negative and positive controls respectively. As a PCR control, the eukaryotic 18S rRNA gene was amplified as described previously [12].
(TIF)

S3 Fig Detection of rickettsial gene-like sequences in 13 strains of *Volvox carteri*. Rickettsial gene-like sequences were amplified via genomic PCR using rickettsia-specific primer sets (see Materials and Methods). For PCR amplification, 12 endosymbiont-lacking strains of *V*. *carteri*, endosymbiont-containing *V*. *carteri* f. *weismannia* strain UTEX 2180 (positive control) and *Chlamydomonas reihnardtii* strain CC-503 (negative control) were used. As a control, the eukaryotic 18S rRNA gene was amplified.
(TIF)

S4 Fig Phylogenetic positions of rickettsial *murB* gene-like sequences from endosymbiont-lacking strains of *Volvox carteri*. The tree was inferred based on translated rickettsial *murB* genes and gene-like sequences (227 amino acid sites) from endosymbiont-lacking strains of *V*. *carteri* (boldface), with 26 translated *murB* sequences from bacteria and possible endosymbionts (En) of algal hosts in the family *Rickettsiaceae*, using the maximum-likelihood (ML) method. Bootstrap values (≥50%) for the ML and maximum parsimony analyses are indicated at the respective nodes. The scale bar corresponds to 0.1 amino acid substitutions per position.
(TIF)

S5 Fig Phylogenetic positions of rickettsial *ddlB* gene-like sequences from endosymbiont-lacking strains of *Volvox carteri*. The tree was inferred based on translated rickettsial *ddlB* genes and gene-like sequences (361 amino acid sites) from endosymbiont-lacking strains of *V*. *carter* (boldface) with 28 translated *ddlB* sequences from bacteria and possible endosymbionts (En) of algal hosts in the family *Rickettsiaceae*, using the maximum-likelihood (ML) method. Bootstrap values (≥50%) for the ML and maximum parsimony analyses are indicated at the respective nodes. The scale bar corresponds to 0.1 amino acid substitutions per position.
(TIF)

S1 File Alignments of three rickettsial genes/gene-like sequences (16S rRNA, *murB* and *ddlB*) and nuclear ribosomal DNA internal transcribed spacer 2 region used in phylogenetic analyses. The sequences of *murB* and *ddlB* are translated. Combined alignment of translated *murB* and *ddlB* is also shown. These alignments are also available from TreeBASE (http://treebase.org/treebase-web/home.html; study ID: 16773).
(PDF)

S2 File Whole secondary structure of nuclear ribosomal DNA internal transcribed spacer 2 region of the strains of *Volvox obversus* and *V*. *carteri*. The structure was predicted and drawn as described in Materials and Methods. The U-U mismatch in helix 2 (arrowheads) and the UGGU motif on the 5′ side near the apex of helix 3 (boldface) are the universally conserved features [43]. Among the ITS-2 sequences of *V*. *carteri* f. *nagariensis* strains, one nucleotide of UTEX 1886 and NIES-398, and three of UTEX 2903 are different from those of strains EVE and NIES-397 (shown around the structures). ITS-2 sequences of *V*. *carteri* f. *weismannia* strains UTEX 1874 and UTEX 2904 differ from that of *V*. *carteri* f. *weismannia* strain UTEX 2180 in missing of one pair of “AU” from AU repeats in helix 4 (asterisk).
(PDF)

S1 Table The high scoring hits of *Volvox carteri* f. *nagariensis* strain EVE genome sequence by BLASTN searches using *Carteria cerasiformis* rickettsial endosymbiont draft genome. BLASTN searches were performed on Phytozome v9.1, using 81 contigs (>5 kb) from preliminary genome assembly database of *C*. *cerasiformis* NIES-425 rickettsial endosymbiont. Hits with the E-value ≤1.0e-50 of BLASTN searches and the information of *ftsQ*-like sequence found in following BLASTN search in scaffold 6 of EVE genome are shown.
(DOC)

S2 Table The list of primers used in genomic PCR, semi-quantitative genomic PCR and sequencing of this study. (DOC)

S3 Table List of *Volvox carteri* and other algal strains used in this study. DDBJ/EMBL/GenBank accession numbers of nuclear ribosomal DNA internal transcribed spacer regions and rickettsial gene-like sequences determined in this study are also shown.
(DOC)

S4 Table DDBJ/EMBL/GenBank accession numbers of the rickettsial *murB* and *ddlB* genes/gene-like sequences used in this study. (DOC)
