# Supplementary figures and images for: Two Different Rickettsial Bacteria Invading Volvox carteri
Source: PLoS One. 2015 Feb 11;10(2):e0116192. doi: 10.1371/journal.pone.0116192 (PMC4324946; doi:10.1371/journal.pone.0116192)

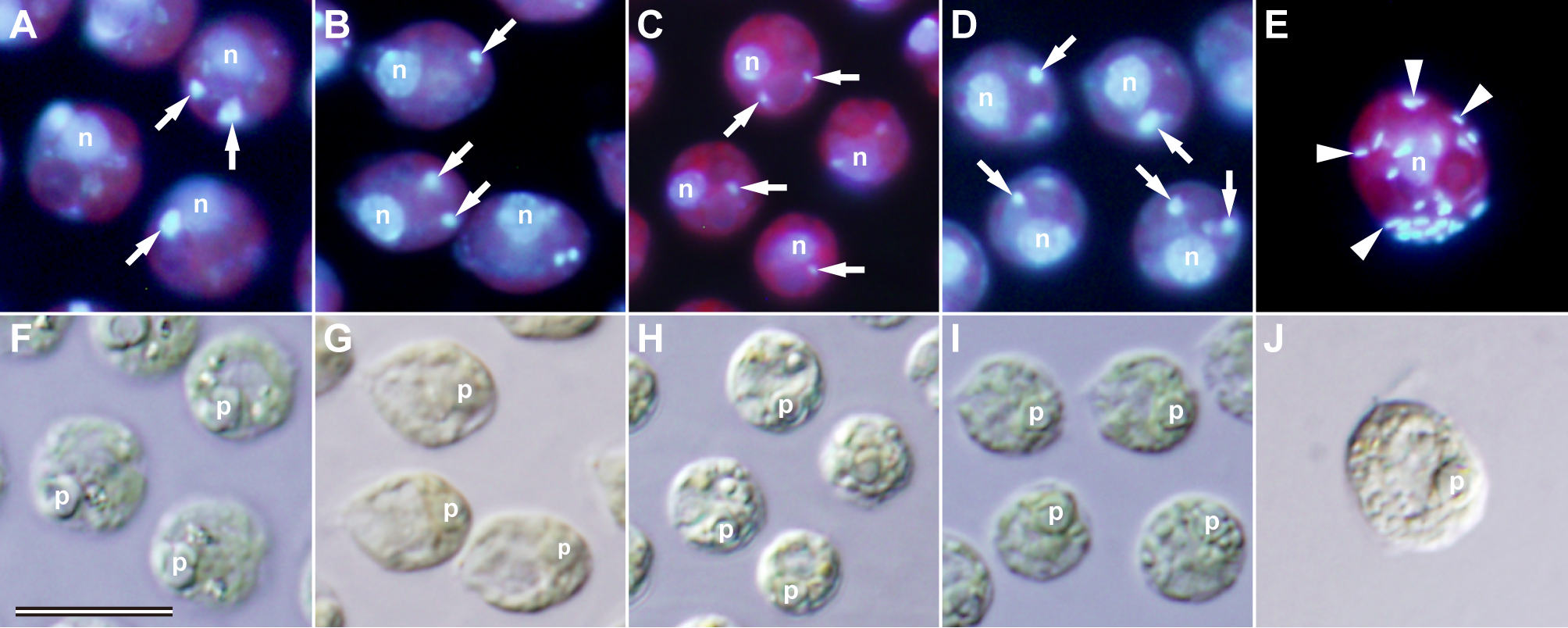

Supplement: S1 Fig — Vertical panels show the same cells shown at the same magnification, composed of epifluorescence images (A-E) and Nomarski differential interference images (F-J). The arrow, ‘n’ and ‘p’ indicate the chloroplast nucleoid, host cell nuclei and pyrenoid respectively. Scale bar = 10 μm. Any bacteria-like rod-shaped bodies were not observed in the cells of f. weismannia strain UTEX 1874 (A, F), f. nagariensis strain UTEX 1886 (B, G), f. nagariensis strain UTEX 2903 (C, H) and f. weismannia strain UTEX 2904 (D, I). On the other hand, the bacterial endosymbionts (arrowheads) were observed in the cell of f. weismannia strain UTEX 2180 (E, J) as rod-shaped fluorescent bodies as observed previously [12]. (TIF) [file pone.0116192.s001.tif]

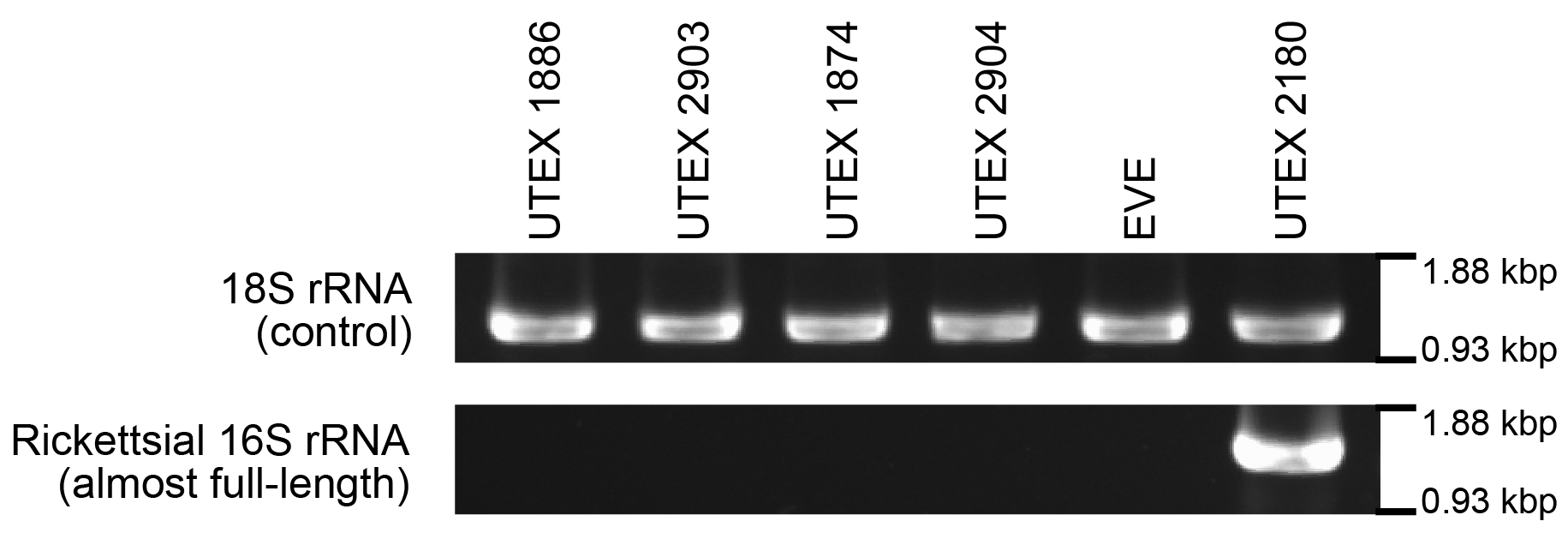

Supplement: S2 Fig — PCR amplification using the forward primer eveFC and reverse primer enRB (specific 16S rRNA primers specific to the bacteria belonging to the hydra group, see S2 Table) corresponds the presence or absence of rickettsial endosymbionts. EVE (lacking rickettsial endosymbiont) and UTEX 2180 (having rickettsial endosymbiont) are shown as negative and positive controls respectively. As a PCR control, the eukaryotic 18S rRNA gene was amplified as described previously [12]. (TIF) [file pone.0116192.s002.tif]

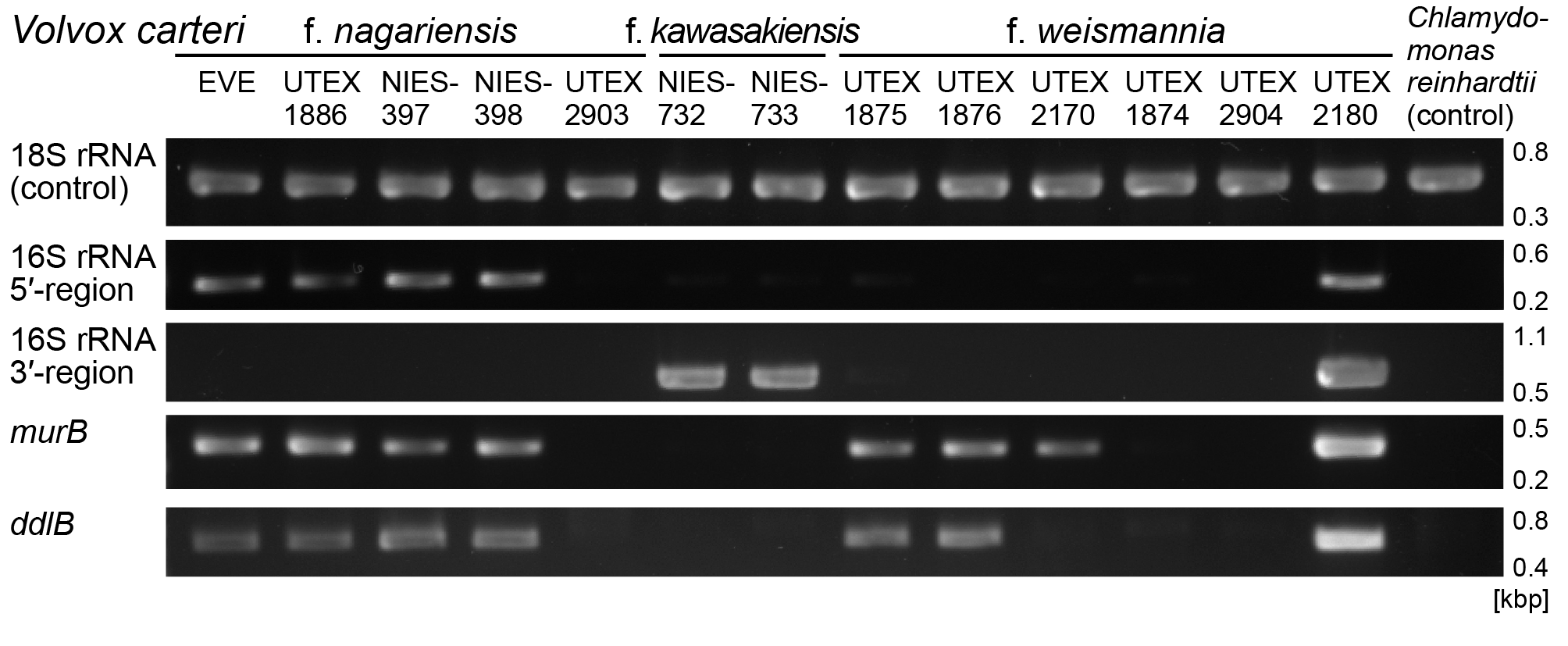

Supplement: S3 Fig — Rickettsial gene-like sequences were amplified via genomic PCR using rickettsia-specific primer sets (see Materials and Methods). For PCR amplification, 12 endosymbiont-lacking strains of V. carteri, endosymbiont-containing V. carteri f. weismannia strain UTEX 2180 (positive control) and Chlamydomonas reihnardtii strain CC-503 (negative control) were used. As a control, the eukaryotic 18S rRNA gene was amplified. (TIF) [file pone.0116192.s003.tif]

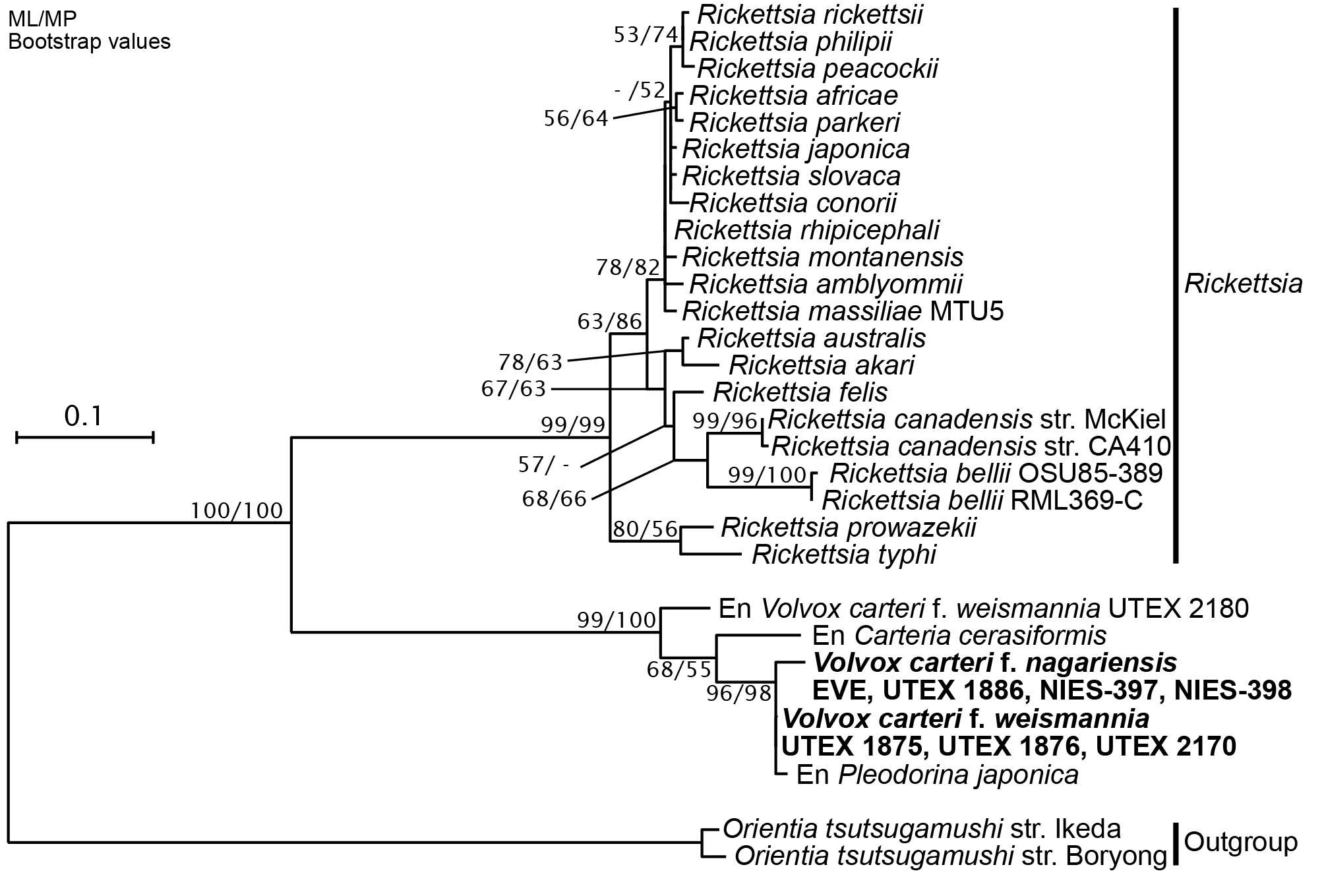

Supplement: S4 Fig — The tree was inferred based on translated rickettsial murB genes and gene-like sequences (227 amino acid sites) from endosymbiont-lacking strains of V. carteri (boldface), with 26 translated murB sequences from bacteria and possible endosymbionts (En) of algal hosts in the family Rickettsiaceae, using the maximum-likelihood (ML) method. Bootstrap values (≥50%) for the ML and maximum parsimony analyses are indicated at the respective nodes. The scale bar corresponds to 0.1 amino acid substitutions per position. (TIF) [file pone.0116192.s004.tif]

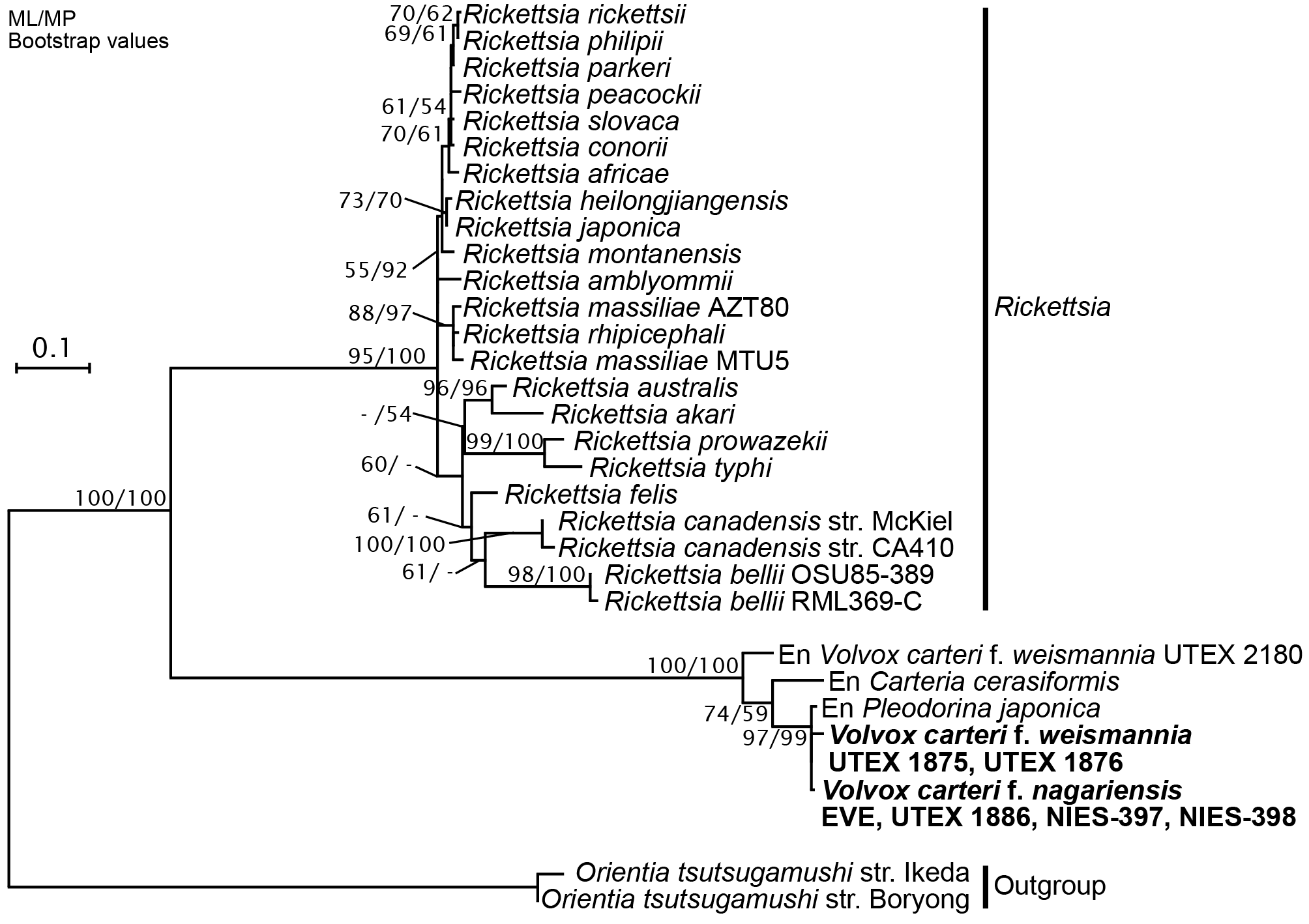

Supplement: S5 Fig — The tree was inferred based on translated rickettsial ddlB genes and gene-like sequences (361 amino acid sites) from endosymbiont-lacking strains of V. carter (boldface) with 28 translated ddlB sequences from bacteria and possible endosymbionts (En) of algal hosts in the family Rickettsiaceae, using the maximum-likelihood (ML) method. Bootstrap values (≥50%) for the ML and maximum parsimony analyses are indicated at the respective nodes. The scale bar corresponds to 0.1 amino acid substitutions per position. (TIF) [file pone.0116192.s005.tif]
