# Supplementary material for: Two Different Rickettsial Bacteria Invading Volvox carteri
Source: PLoS One. 2015 Feb 11;10(2):e0116192. doi: 10.1371/journal.pone.0116192 (PMC4324946; doi:10.1371/journal.pone.0116192)
Supplement: S1 File — The sequences of murB and ddlB are translated. Combined alignment of translated murB and ddlB is also shown. These alignments are also available from TreeBASE (http://treebase.org/treebase-web/home.html; study ID: 16773). (PDF) [file pone.0116192.s006.pdf]

Supporting Information File S1
16S rRNA

```
           1
RicRicIo   ATCGGTATGC TT-AACACAT GCAAGTCGAA CGGACTA-AT TGGGG-CTTG -CTCCAAT-T A-GTTAGTGG CAGACGGGTG AGTAACACGT GGGAATCTAC CCATCAGTAC
RicLimo2   TTCGGTATGC TT-AACACAT GCAAGTTGAA CGGATTA-AT TTAGGGCTTG CTCTAAAT-T A-GTTAGTAG CAGACGGGTG AGTAACACGT GGGAATCTGC CCATCAGTAC
RicFeli2   ATCGGTATGC TT-AACACAT GCAAGTCGGA CGGACTA-AT TGGGG-CTTG -CTCCAAT-T A-GTTAGTGG CAGACGGGTG AGTAACACGT GGGAATCTGC CCATCAGTAC
RicProwa   ATCGGTATGC TT-AACACAT GCAAGTCGAA CGGATTA-AC TAGAG-CTCG -CTTTAGT-T A-ATTAGTGG CAGACGGGTG AGTAACACGT GGGAATCTAC CCATCAGTAC
RicTyphi   ATCGGTATGC TT-AACACAT GCAAGTCGAA CGGATTA-AT TAGAG-CTTG -CTCTAGT-T A-ATTAGTGG CAGACGGGTG AGTAACACGT GGGAATCTAC CCATCAGTAC
RicCono4   ATCGGTATGC TT-AACACAT GCAAGTCGAA CGGACTA-AT TGGGG-CTTG -CTCCAGT-T A-GTTAGTGG CAGACGGGTG AGTAACACGT GGGAATCTAC CCATTAGTAC
RicEmpPa   GGCGGTATGC TT-AACACAT CGAAGTCGAA CGAACTA-AT TGGGG-CTTG -CTCCAAT-T A-GTTAGTGG CAGACGGGTG AGTAACACGT GGGAATCTGC CCATCAGTAC
RicJapon   ATCGGTATGC TT-AACACAT GCAAGTCGAA CGGACTA-AT TGGGG-CTTG -CTCCAAT-T A-GTTAGTGG CAGACGGGTG AGTAACACGT GGGAATCTAC CCATCAGTAC
RicCana2   ATCGGTATGC TT-AACACAT GCAAGTCGAA CGGACTA-AT TAGGG-CTTG -CTCTAAT-T A-GTTAGTGG CAGACGGGTG AGTAACACGT GGGAATCTGC CCATCAGTAC
RicHelve   ATCGGTATGC TT-AACACAT GCAAGTCGAA CGGACTA-AT TGGGG-CTTG -TTCCAGT-T A-GTTAGTGG CAGACGGGTG AGTAACACGT GGGAATCTAC CCATCAGTAT
RicAust3   ATCGGTATGC TT-AACACAT GCAAGTCGGA CGGACTA-AT TGGGGGTTTA -CTCTAAT-T A-GTTAGTGG CAGACGGGTG AGTAACACGT GGGAATCTGC CCATCAGTAC
RicBell3   ATCGGTATGC TT-AACACAT GCAAGTCGAA CGGACTA-AT TGGGG-CTTG -CTCCAAT-T A-GTTAGTGG CAGACGGGTG AGTAACACGT GGGAATCTGC CCATCAGTAC
RicAkar3   ATCGGTATGC TT-AACACAT GCAAGTCGGA CGGACTG-AT TGGGG-TTTT -CTCCAGT-T A-GTGAGTGG CGAACGGGTG AGTAACACGT GGGAATCTGC CCATCAGTAC
TorTsuku   TTCGGTATGC TTAAACACAT GCAAGTTGAA CGGATTA-AT TTAGGGCTTG CTCTGAAT-T A-GTTAGTAG CAAACGGGTG AGTAACACGT GGGAATCTGC CCATCAGTAC
TorTagoi   TTCGGTATGC TT-AACACAT GCAAGTTGAA CGGATTA-AT TTAGGGCTTG CTCTGAAT-T A-GTTAGTAG CAAACGGGTG AGTAACACGT GGGAATCTGC CCATCAGTAC
HemMarg2   TTCGGTATGC TT-AACACAT GCAAGTTGAA CGGATTA-AT TTAGGGCTTG CTCTGAAT-T A-GTTAGTAG CAAACGGGTG AGTAACACGT GGGAATCTGC CCATCAGTAC
HydRick1   GGCGGTATGC TT-AACACAT GCAAGTCGAA CGAATGCCTT TGGAG-CTTG -CTTCAAAGT C-GTTAGTGG CAAACGGGTG AGTAACACGT GGGAATCTAC CTATCAGTAC
IchMulti   GGCGGTATGC TT-AACACAT GCAAGTCGAA CGGATGCCTT TGGAG-CTTG -CTCCAAAAG C-GTTAGTGG CAAACGGGTG AGTAACGCGT GGGAATCTAC CTATCAGTAC
BryRick1   GGCGGTATGC TT-AACACAT GCAAGTCGAA CGAACAAGGC TGGAG-CTTG -CTTCAGCTG C-GTTAGTGG CAAACGGGTG AGTAACACGT GGGAATCTAC CCATCAGTAC
BryRick2   GGCGGTATGC TT-AACACAT GCAAGTCGAA CGAACAAAGC TGGAG-CTTG -CTTCAGTTT T-GTTAGTGG CAAACGGGTG AGTAACACGT GGGAATCTAC CCATCAGTAC
BryRick3   GGCGGTATGC TT-AACACAT GCAAGTCGAA CGAACAAGGC TGGAG-CTTG -CTTCAGCTG C-GTTAGTGG CAAACGGGTG AGTAACACGT GGGAATCTAC CCATCAGTAC
MonFaveo   GGCGGTATGC TT-AACACAT GCAAGTCGAA CGAACAAAGC TGGAG-CTTG -CTTCAGTGG C-GTGAGTGG CAAACGGGTG AGTAACACGT GGGAATCTAC CCATCAGTAC
RicNepCi   TTCGGTATGC TT-AACACAT GCAAGTTGAA CGGATTA-AT TTAGGGCTTG CTCTGAAT-T A-GTTAGTAG CAAACGGGTG AGTAACACGT GGGAATCTAC CCATCAGTAC
Kalahari   GGCGGTATGC TT-AACACAT GCAAGTCGAA CGAACAAAAT TGGGG-CTTG -CTCCAATTT TAGTGAGTGG CAAACGGGTG AGTAACACGT GGGAATCTAC CTATCAGTAC
T6025C27   GGCGGTATGC TT-AACACAT GCAAGTCGAA CGAACAAAAT CAGGG-CTTG -CTCTGATTT TAGTGAGTGG CAAACGGGTG AGTAACACGT GGGAATCTAC CCATCAGTAC
T6025C01   GGCGGTATGC TT-AACACAT GCAAGTCGAA CGAACAAAAT TGGGG-CTTG -CTCCAATTT TAGTGAGTGG CAAACGGGTG AGTAACACGT GGGAATCTAC CTATCAGTAC
WakWat77   GGCGGTATGC TT-AACACAT GCAAGTCGAA CGAACAAAAT TGGGG-CTTG -CTCCAATTT TAGTGAGTGG CAAACGGGTG AGTAACACGT GGGAATCTAC CCATCAGTAC
Waste214   GGCGGTATGC TT-AACACAT GCAAGTCGAA CGAACAAAAT TGGGG-CTTG -CTCCAATTT TAGTGAGTGG CAAACGGGTG AGTAACACGT GGGAATCTAC CTATCAGTAC
TaihAS94   GGCGGTATGC TT-AACACAT GCAAGTCGAA CGAACAAAAT TGGGG-CTTG -CTCCAATTT TAGTGAGTGG CAAACGGGTG AGTAACACGT GGGAATCTAC CTATCAGTAC
HydRick2   GGCGGTATGC TT-AACACAT GCAAGTCGAA CGAATGCCTT TGGAG-CTTG -CTTCAAAGT C-GTTAGTGG CAAACGGGTG AGTAACACGT GGGAATCTAC CTATCAGTAC
DioOBOD9   GGCGGTATGC TT-AACACAT GCAAGTCGAA CGAACAAAAT TGGGG-CTTG -CTCCAATTT TAGTGAGTGG CAAACGGGTG AGTAACACGT GGGAATCTAC CTATCAGTAC
PrmCauda   GGCGGTATGC TT-AACACAT GCAAGTCGAA CGAACAAAAT TGGGG-CTTG -CTCCAATTT TAGTGAGTGG CAAACGGGTG AGTAACACGT GGGAATCTAC CTATCAGTAC
Sprsmm01   GGCGGTGTGC TT-AACACAT GCAAGTCAAA CGAACAAAAT TGGGG-CTTG -CTCCTATTT TAGTGAGTGG CAAACGGGTG TGTAACACGT GGGAATCTAC CTATCAGTAC
DioDS124   GGCGGTATGC TT-AACACAT GCAAGTCGAA CGAACAAAAT TGGGG-CTTG -CTCCAATTT TAGTGAGTGG CAAACGGGTG AGTAACACGT GGGAATCTAC CTATCAGTAC
EupOct01   GGCGGTATGC TT-AACACAT GCAAGTCGAA CGAACAAAAT TGGGG-CTTG -CTCCAATTT TAGTGAGTGG CAAACGGGTG AGTAACACGT GGGAATCTAC CTATCAGTAC
NIES_425   GGCGGTATGC TT-AACACAT GCAAGTCGAA CGAACAAAAT TGGGG-CTTG -CTCCAATTT TAGTGAGTGG CAAACGGGTG AGTAACACGT GGGAATCTAC CTATCAGTAC
NIES_577   GGCGGTATGC TT-AACACAT GCAAGTCGAA CGAACAAAAT TGGGG-CTTG -CTCCAATTT TAGTGAGTGG CAAACGGGTG AGTAACACGT GGGAATCTAC CTATCAGTAC
UTEX2180   GGCGGTATGC TT-AACACAT GCAAGTCGAA CGAACAAAAT TGGGG-CTTG -CTCCAATTT TAGTGAGTGG CAAACGGGTG AGTAACACGT GGGAATCTAC CTATCAGTAC
EVE______  GGCGGTATGC TT-AACACAT GCAAGTCGAA CGAACAAAAT TGGGG-CTTG -CTCCAATTT TAGTGAGTGG CAAACGGGTG AGTAACACGT GGGAATCTAC CTATCAGTAC
NIES_732   ---------- ---------- ---------- ---------- ---------- ---------- ---------- ---------- ---------- ---------- ----------
OrnTsIke   GGCGGCATGC CT-AACACAT GCAAGTCGAA CGAATTAATG CTGAG-CTTG -CTTAGCATT A-ATTAGTGG CAAACGGGTG AGTAACACGT GGGAATCTGC CCATCAGTAC
OrnTsBor   GGCGGCATGC CT-AACACAT GCAAGTCGAA CGAATTAATG CTGAG-CTTG -CTTAGCATT A-ATTAGTGG CAAACGGGTG AGTAACACGT GGGAATCTGC CCATCAGTAC
CprPolyt   AGCGGCATGC TT-AACACAT GCAAGTCGAA CGAATGTATT TCTAG-CTTG -CTAGAGATA T-GTTAGTGG CATACGGGTG AGTAACACGT GGGAATCTGC CCATCAGTAT
```

```
           111
RicRicIo   GGAATAACTT TTAGAAATAA AAGCTAATAC CGTATATTCT CTGCG--GAG GAAAGATTTA TCGCTGATGG ATGAGCCCGC GTCAGATTAG GTAGTTGGTG AGGTAATGGC
RicLimo2   GGAATAACAT TTAGAAATAA ATGCTAATAC CGTATATTCT CTTCG--GAG GAAAGATTTA TCGCTGATGG ATGAGCCCGC GTCAGATTAG GTAGTTGGTA GGGTAATGGC
RicFeli2   GGAATAACTT TTAGAAATAA AAGCTAATAC CGTATATTCT CTACA--GAG GAAAGATTTA TCGCTGATGG ATGAGCCCGC GTCAGATTAG GTAGTTGGTG AGGTAACGGC
RicProwa   GGAATAACTT TTAGAAATAA AAGCTAATAC CGTATATTCT CTACG--GAG GAAAGATTTA TCGCTGATGG ATGGGCCCGC GTCAGATTAG GTAGTTGGTG AGGTAATGGC
RicTyphi   GGAATAACTT TTAGAAATAA AAGCTAATAC CGTATATTCT CTACG--GAG GAAAGATTTA TCGCTGATGG ATGGGCCCGC GTCAGATTAG GTAGTTGGTG TGGTAATGGC
RicCono4   GGAATAACTT TTAGAAATAA AAGCTAATAC CGTATATTCT CTGCG--GAG GAAAGATTTA TCGCTGATGG ATGAGCCCGC GTCAGATTAG GTAGTTGGTG AGGTAATGGC
RicEmpPa   GGAATAACTT TTAGAAATAA AAGCTAATAC CGTATATTCT CTACG--GAG GAAAGATTTA TCGCTGATGG ATGAGCCCGC GTCAGATTAG GTAGTTGGTG AGGTAATGGC
RicJapon   GGAATAACTT TTAGAAATAA AAGCTAATAC CGTATATTCT CTGCG--GAG GAAAGATTTA TTGCTGATGG ATGAGCCCGC GTCAGATTAG GTAGTTGGTG AGGTAATGGC
RicCana2   GGAATAACTT TTAGAAATAA AAGCTAATAC CGTATATTCT CTACA--GAG GAAAGATGTA TCGCTGATGG ATGAGCCCGC GTCAGATTAG GTAGTTGGTG AGGTAATGGC
RicHelve   GGAATAACTT TTAGAAATAA AAGCTAATAC CATATATTCT CTATG--GAG GAAAGATTTA TCGCTGATGG ATGAGCCCGC GTCAGATTAG GTAGTTGGTG AGGTAACGGC
RicAust3   GGAATAACTT TTAGAAATAA AAGCTAATAC CGTATATTCT CTACG--GAG GAAAGATTTA TCGCTGATGG ATGAGCCCGC GTCAGATTAG GTAGTTGGTG AGGTAACGGC
RicBell3   GGAATAACTT TTAGAAATAA AAGCTAATAC CGTATATTCT CTACG--GAG GAAAGATTTA TCGCTGATGG ATGAGCCCGC GTCAGATTAG GTAGTTGGTG AGGTAATGGC
RicAkar3   GGAATAACTT TTAGAAATAA AAGCTAATAC CGTATATTCT CTACG--GAG GAAAGATTTA TCGCTGATGG ATGAGCCCGC GTCGGATTAG GTAGTTGGTG AGGTAACGGC
TorTsuku   GGAATAACAC TTAGAAATAA ATGCTAATAC CGTATATTCT CTTCG--GAG GAAAGATTTA TCGCTGATGG ATGAGCCCGC GTCAGATTAG GTAGTTGGTA GGGTAATGGC
TorTagoi   GGAATAACAC TTAGAAATAA ATGCTAATAC CGTATATTCT CTTCG--GAG GAAAGATTTA TCGCTGATGG ATGAGCCCGC GTCAGATTAG GTAGTTGGTA GGGTAATGGC
HemMarg2   GGAATAACAC TTAGAAATAA ATGCTAATAC CGTATATTCT CTTCG--GAG GAAAGATTTA TCGCTGATGG ATGAGCCCGC GTCAGATTAG GTAGTTGGTA GGGTAATGGC
HydRick1   GGAATAACAT TTGGAAACAG ATGCTAATAC CGTATATTCT CCTCG--GAG GAAAGATTTA TCGCTGATAG ATGAGCCCGC GTAAGATTAG GTAGTTGGTA GGGTAATGGC
IchMulti   GGAATAACAT TTGGAAACAG ATGCTAATAC CGTATATTCT CTTCG--GAG GAAAGATTTA TCGCTGATAG ATGAGCCCGC GTAAGATTAG GTAGTTGGTG GGGTAATAGC
BryRick1   GGAATAACAT TTGGAAACAA ATGCTAATAC CGTATATTCT CTACG--GAG GAAAGATTTA TCGCTGATGG ATGAGCCCGC GCAAGATTAG GTAGTTGGTA GGGTAATGGC
BryRick2   GGAATAACAT TTGGAAACAG ATGCTAATAC CGTATATTCT CCAAG--GAG GAAAGATTTA TCGCTGATGG ATGAGCCCGC GCAAGATTAG GTAGTTGGTA GGGTAATGGC
BryRick3   GGAATAACAT TTGGAAACAA ATGCTAATAC CGTATATTCT CTACG--GAG GAAAGATTTA TCGCTGATGG ATGAGCCCGC GCAAGATTAG GTAGTTGGTA GGGTAATGGC
MonFaveo   GGAATAATAT TTGGAAACAA ATGCTAATAC CGTATATTCT CTACG--GAG GAAAGATTTA TCGCTGATGG ATGAGCCCGC GTAAGATTAG GTAGTTGGTG GGGTAATGGC
RicNepCi   GGAATAACAC TTAGAAATAA ATGCTAATAC CGTATATTCT CTTCG--GAG GAAAGATTTA TCGCTGATGG ATGAGCCCGC GTCAGATTAG GTAGTTGGTA GGGTAATGGC
Kalahari   GGAATAACAT TTGGAAACAG ATGCTAATAC CGTATATTCT CCAAG--GAG GAAAGATTTA TCGCTGATAG ATGAGCCCGC GTAAGATTAG GTAGTTGGTA GGGTAATGGC
T6025C27   GGAATAACAT TTGGAAACAA ATGCTAATAC CGTATATTCT CTAAG--GAG GAAAGATTTA TCGCTGATGG ATGAGCCCGC GCAAGATTAG GTAGTTGGTA GGGTAATGGC
T6025C01   GGAATAACAT TTGGAAACAA ATGCTAATAC CGTATATTCT CCAAG--GAG GAAAGATTTA TCGCTGATAG ATGAGCCCGC GCAAGATTAG GTAGTTGGTA GGGTAATGGC
WakWat77   GGAATAACAT TTGGAAACAA ATGCTAATAC CGTATATTCT CTAAG--GAG GAAAGATTTA TCGCTGATGG ATGAGCCCGC GTAAGATTAG GTAGTTGGTA GGGTAATGGC
Waste214   GGAATAACAT TTGGAAACAG ATGCTAATAC CGTATATTCT CCAAG--GAG GAAAGATTTA TCGCTGATAG ATGAGCCCGC GCAAGATTAG GTAGTTGGTA GGGTAATGGC
TaihAS94   GGAATAACAT TTGGAAACAG ATGCTAATAC CGTATATTCT CCAAG--GAG GAAAGATTTA TCGCTGATAG ATGAGCCCGC GCAAGATTAG GTAGTTGGTA GGGTAATGGC
HydRick2   GGAATAACAT TTGGAAACAG ATGCTAATAC CGTATATTCT CCTCG--GAG GAAAGATTTA TCGCTGATAG ATGAGCCCGC GTAAGATTAG GTAGTTGGTA GGGTAATGGC
DioOBOD9   GGAATAACAT TTGGAAACAG ATGCTAATAC CGTATATTCT CCAAG--GAG GAAAGATTTA TCGCTGATAG ATGAGCCCGC GTAAGATTAG GTAGTTGGTA GGGTAATGGC
PrmCauda   GGAATAACAT TTGGAAACAG ATGCTAATAC CGTATATTCT CCAAG--GAG GAAAGATTTA TCGCTGATAG ATGAGCCCGC GTAAGATTAG GTAGTTGGTA GGGTAATGGC
Sprsmm01   GGAATAACAT TTGGAAACAG ATGCTAATAC CGTATATTCT CCAAG--GAG GAAAGATTTA TCGCTGATAG ATGAGCCCGC GTAAGATTAG GTAGTTGGTA GGGTAATGGC
DioDS124   GGAATAACAT TTGGAAACAG ATGCTAATAC CGTATATTCT CCAAG--GAG GAAAGATTTA TCGCTGATAG ATGAGCCCGC GCAAGATTAG GTAGTTGGTA GGGTAATGGC
EupOct01   GGAATAACAT TTGGAAACAG ATGCTAATAC CGTATATTCT CCAAG--GAG GAAAGATTTA TCGCTGATAG ATGAGCCCGC GTAAGATTAG GTAGTTGGTA GGGTAATGGC
NIES_425   GGAATAACAT TTGGAAACAG ATGCTAATAC CGTATATTCT CCAAG--GAG GAAAGATTTA TCGCTGATAG ATGAGCCCGC GTAAGATTAG GTAGTTGGTA GGGTAATGGC
NIES_577   GGAATAACAT TTGGAAACAA ATGCTAATAC CGTATATTCT CCAAG--GAG GAAAGATTTA TCGCTGATAG ATGAGCCCGC GCAAGATTAG GTAGTTGGTA GGGTAATGGC
UTEX2180   GGAATAACAT TTGGAAACAG ATGCTAATAC CGTATATTCT CCAAG--GAG GAAAGATTTA TCGCTGATAG ATGAGCCCGC GCAAGATTAG GTAGTTGGTA GGGTAATGGC
EVE_____   GGAATAACAT TTGGAAACAG ATGCTAATAC CGTATATTCT CTAAG--GAG GAAAGATTTA TCGCTGATAG ATGAGCCCGC GCAAGATTAG GTAGTTGGTA GGGTAATGGC
NIES_732   ---------- ---------- ---------- ---------- ---------- ---------- ---------- ---------- ---------- ---------- ----------
OrnTsIke   GGAATAACAT TTAGAAATAA GTGCTAATAC CGTATGCCCT CTACAAGGAG GAAAGATTTA TCGCTGATGG ATGAGCCCGC GGCAGATTAG GTAGTTGGTA AGGTAATGGC
OrnTsBor   GGAATAACAT TTAGAAATAA GTGCTAATAC CGTATGCCCT CTATAAGGAG GAAAGATTTA TCGCTGATGG ATGAGCCCGC GGCAGATTAG GTAGTTGGTA AGGTAATGGC
CprPolyt   GGAATAACTT TGGGAAACTA AAGCTAATAC CATATATTTT CTACG--GAA GAAAGATTTA TCGCTGATGG ATGAGCCCGC GGCAGATTAG CTAGTTGGTG GGGTAAAGGC
```

```
          221
RicRicIo  TCACCAAGCC GACGATCTGT AGCTGGTCTG AGAGGATGAT CAGCCACACT GGGACTGAGA CACGGCCCAG ACTCCTACGG GAGGCAGCAG TGGGGAATAT TGGACAATGG
RicLimo2  CTACCAAGCC AACGATCTGT AGCTGGTCTG AGAGGATGAT CAGCCACACT GGGACTGAGA CACGGCCCAG ACTCCTACGG GAGGCAGCAG TGGGGAATAT TGGACAATGG
RicFeli2  TCACCAAGCC GACGATCTGT AGCTGGTCTG AGAGGATGAT CAGCCACACT GGGACTGAGA CACGGCCCAG ACTCCTACGG GAGGCAGCAG TGGGGAATAT TGGACAATGG
RicProwa  TCACCAAGCC GACGATCTGT AGCTGGTCTG AGAGGATGAT CAGCCACACT GGGACTGAGA CACGGCCCAG ACTCCTACGG GAGGCAGCAG TGGGGAATAT TGGACAATGG
RicTyphi  TCACCAAGCC GACGATCTGT AGCTGGTCTG AGAGGATGAT CAGCCACACT GGGACTGAGA CACGGCCCAG ACTCCTACGG GAGGCAGCAG TGGGGAATAT TGGACAATGG
RicCono4  TCACCAAGCC GACGATCTGT AGCTGGTCTG AGAGGATGAT CAGCCACACT GGGACTGAGA CACGGCCCAG ACTCCTACGG GAGGCAGCAG TGGGGAATAT TGGACAATGG
RicEmpPa  TCACCAAGCC TACGATCTGT AGCTGGTCTG AGAGGATGAT CAGCCACACT GGGACTGAGA CACGGCCCAG ACTCCTACGG GAGGCAGCAG TGGGGAATAT TGGACAATGG
RicJapon  TCACCAAGCC GACGATCTGT AGCTGGTCTG AGAGGATGAT CAGCCACACT GGGACTGAGA CACGGCCCAG ACTCCTACGG GAGGCAGCAG TGGGGAATAT TGGACAATGG
RicCana2  TCACCAAGCC AACGATCTGT AGCTGGTCTG AGAGGATGAT CAGCCACACT GGGACTGAGA CACGGCCCAG ACTCCTACGG GAGGCAGCAG TGGGGAATAT TGGACAATGG
RicHelve  TCACCAAGCC GACGATCTGT AGCTGGTCTG AGAGGATGAT CAGCCACACT GGGACTGAGA CACGGCCCAG ACTCCTACGG GAGGCAGCAG TGGGGAATAT TGGACAATGG
RicAust3  TCACCAAGCC GACGATCTGT AGCTGGTCTG AGAGGATGAT CAGCCACACT GGGACTGAGA CACGGCCCAG ACTCCTACGG GAGGCAGCAG TGGGGAATAT TGGACAATGG
RicBell3  TCACCAAGCC TACGATCTGT AGCTGGTCTG AGAGGATGAT CAGCCACACT GGGACTGAGA CACGGCCCAG ACTCCTACGG GAGGCAGCAG TGGGGAATAT TGGACAATGG
RicAkar3  TCACCAAGCC GACGATCTGT AGTTGGTCTG AGAGGATGAT CAGCCACACT GGGACTGAGA CACGGCCCAG ACTCCTACGG GAGGCAGCAG TGGGGAATAT TGGACAATGG
TorTsuku  CTACCAAGCC AACGATCTGT AGCTGGTCTG AGAGGATGAT CAGCCACACT GGGACTGAGA CACGGTCCAG ACTCCTACGG GAGGCAGCAG TGGGGAATAT TGGACAATGG
TorTagoi  CTACCAAGCC AACGATCTGT AGCTGGTCTG AGAGGATGAT CAGCCACACT GGGACTGAGA CACGGTCCAA ACTCCTACGG GAGGCAACAG TGGGGAATAT TGGACAATGG
HemMarg2  CTACCAAGCC AACGATCTGT AGCTGGTCTG AGAGGATGAT CAGCCACACT GGGACTGAGA CACGGCCCAG ACTCCTACGG GAGGCAGCAG TGGGGAATAT TGGACAATGG
HydRick1  CTACCAAGCC GACGATCTTT AGCTGATCTG AGAGGATGAT CAGCCACACT GGGACTGAGA CACGGCCCAG ACTCCTACGG GAGGCAGCAG TGGGGAATAT TGGACAATGG
IchMulti  CTACCAAGCC GACGATCTTT AGCTGATCTG AGAGGATGAT CAGCCACACT GGGACTGAGA CACGGCCCAG ACTCCTACGG GAGGCAGCAG TGGGGAATAT TGGACAATGG
BryRick1  CTACCAAGCC GACGATCTTT AGCTGGTCTG AGAGGATGAT CAGCCACACT GGGACTGAGA CACGGCCCAG ACTCCTACGG GAGGCAGCAG TGGGGAATAT TGGACAATGG
BryRick2  CTACCAAGCC GACGATCTTT AGCTGGTCTG AGAGGATGAT CAGCCACACT GGGACTGAGA CACGGCCCAG ACTCCTACGG GAGGCAGCAG TGGGGAATAT TGGACAATGG
BryRick3  CTACCAAGCC GACGATCTTT AGCTGGTCTG AGAGGATGAT CAGCCACACT GGGACTGAGA CACGGCCCAG ACTCCTACGG GAGGCAGCAG TGGGGAATAT TGGACAATGG
MonFaveo  CTACCAAGCC GACGATCTTT AGCTGGTCTG AGAGGATGAT CAGCCACACT GGGACTGAGA CACGGCCCAG ACTCCTACGG GAGGCAGCAG TGGGGAATAT TGGACAATGA
RicNepCi  CTACCAAGCC AACGATCTGT AGCTGGTCTG AGAGGATGAT CAGCCACACT GGGACTGAGA CACGGCCCAG ACTCCTACGG GAGGCAGCAG TGGGGAATAT TGGACAATGG
Kalahari  CTACCAAGCC GACGATCTTT AGCTGGTCTG AGAGGATGAT CAGCCACACT GGGACTGAGA CACGGCCCAG ACTCCTACGG GAGGCAGCAG TGGGGAATAT TGGACAATGG
T6025C27  CTACCAAGCC GACGATCTTT AGCTGGTCTG AGAGGATGAT CAGCCACACT GGGACTGAGA CACGGCCCAG ACTCCTACGG GAGGCAGCAG TGGGGAATAT TGGACAATGG
T6025C01  CTACCAAGCC TACGATCTTT AGCTGGTCTG AGAGGATGAT CAGCCACACT GGGACTGAGA CACGGCCCAG ACTCCTACGG GAGGCAGCAG TGGGGAATAT TGGACAATGG
WakWat77  CTACCAAGCC GACGATCTTT AGCTGGTCTG AGAGGATGAT CAGCCACACT GGGACTGAGA CACGGCCCAG ACTCCTACGG GAGGCAGCAG TGGGGAATAT TGGACAATGG
Waste214  CTACCAAGCC TACGATCTTT AGCTGGTCTG AGAGGATGAT CAGCCACACT GGGACTGAGA CACGGCCCAG ACTCCTACGG GAGGCAGCAG TGGGGAATAT TGGACAATGG
TaihAS94  CTACCAAGCC GACGATCTTT AGCTGGTCTG AGAGGATGAT CAGCCACACT GGGACTGAGA CACGGCCCAG ACTCCTACGG GAGGCAGCAG TGGGGAATAT TGGACAATGG
HydRick2  CTACCAAGCC GACGATCTTT AGCTGATCTG AGAGGATGAT CAGCCACACT GGGACTGAGA CACGGCCCAG ACTCCTACGG GAGGCAGCAG TGGGGAATAT TGGACAATGG
DioOBOD9  CTACCAAGCC GACGATCTTT AGCTGGTCTG AGAGGATGAT CAGCCACACT GGGACTGAGA CACGGCCCAG ACTCCTACGG GAGGCAGCAG TGGGGAATAT TGGACAATGG
PrmCauda  CTACCAAGCC GACGATCTTT AGCTGGTCTG AGAGGATGAT CAGCCACACT GGGACTGAGA CACGGCCCAG ACTCTTACGG GAGGCAGCAG TGGGGAATAT TGGACAATGG
Sprsmm01  CTACCAAGCC GACGATCTTT AGCTGGTCTG AGAGGATGAT CAGCCACACT GGGACTGAGA CACGGCCCAG ACTCCTACGG GAGGCAGCAG TGGGGAATAT TGGACAATGG
DioDS124  CTACCAAGCC GACGATCTTT AGCTGGTCTG AGAGGACGAT CAGCCACACT GGGACTGAGA CACGGCCCAG ACTCCTACGG GAGGCAGCAG TGGGGAATAT TGGACAATGG
EupOct01  CTACCAAGCC GACGATCTTT AGCTGGTCTG AGAGGATGAT CAGCCACACT GGGACTGAGA CACGGCCCAG ACTCCTACGG GAGGCAGCAG TGGGGAATAT TGGACAATGG
NIES_425  CTACCAAGCC GACGATCTTT AGCTGGTCTG AGAGGATGAT CAGCCACACT GGGACTGAGA CACGGCCCAG ACTCCTACGG GAGGCAGCAG TGGGGAATAT TGGACAATGG
NIES_577  CTACCAAGCC TACGATCTTT AGCTGGTCTG AGAGGATGAT CAGCCACACT GGGACTGAGA CACGGCCCAG ACTCCTACGG GAGGCAGCAG TGGGGAATAT TGGACAATGG
UTEX2180  CTACCAAGCC GACAATCTTT AGCTGGTCTG AGAGGATGAT CAGCCACACT GGGACTGAGA CACGGCCCAG ACTCCTACGG GAGGCAGCAG TGGGGAATAT TGGACAATGG
EVE_____  CTACCAAGCC TACGATCTTT AGCTGGTCTG AGAGGATGAT CAGCCACACT GGGACTGAAT CACGGCCCAG ACTCCTACGA GAGGCAGCAG TGGGGAATAT TGGACAATGG
NIES_732  ---------- ---------- ---------- ---------- ---------- ---------- ---------- ---------- ---------- ---------- ----------
OrnTsIke  TTACCAAGCC TGTGATCTGT AGCTGGTCTG AGAGGACGAA CAGCCACACT GGGACTGAGA CACGGCCCAG ACTCCTACGG GGGGCAGCAG TGGGGAATAT TGGACAATGG
OrnTsBor  TTACCAAGCC TGTGATCTGT AGCTGGTCTG AGAGGACGAA CAGCCACACT GGGACTGAGA CACGGCCCAG ACTCCTACGG GGGGCAGCAG TGGGGAATAT TGGACAATGG
CprPolyt  CTACCAAGGC AACGATCTGT AGCTGGTCTG AGAGGATGAT CAGCCACACT GGGACTGAGA CACGGCCCAG ACTCCTACGG GAGGCAGCAG TGGGGAATAT TGGACAATGG
```

```
          331
RicRicIo  GCGAAAGCCT GATCCAGCAA TACCGAGTGA GTGATGAAGG CCTTAGGGTT GTAAAGCTCT TTT-AGCAAG GAAGATAATG ACGTTACTTG -CAGAAAAAG CCCCGGCTAA
RicLimo2  GCGAAAGCCT GATCCAGCAA TACCGAGTGG GTGACGAAGG CCTTAGGGTT GTAAAGCCCT TTTCAGCAGG GAAGATAATG ACGGTACCTG ACCAAGAAAG CCCCGGCTAA
RicFeli2  GCGAAAGCCT GATCCAGCAA TACCGAGTGA GTGATGAAGG CCCTAGGGTT GTAAAGCTCT TTT-AGCAAG GAAGATAATG ACGTTACTTG -CAGAAAAAG CCCCGGCTAA
RicProwa  GCGAAAGCCT GATCCAGCAA TACCGAGTGA GTGATGAAGG CCTTAGGGTT GTAAAGCTCT TTT-AGCAAG GAAGATAATG ACGTTACTTG -CAGAAAAAG CCCCGGCTAA
RicTyphi  GCGAAAGCCT GATCCAGCAA TACCGAGTGA GTGATGAAGG CCTTAGGGTT GTAAAGCTCT TTT-AGCAAG GAAGATAATG ACGTTACTTG -CAGAAAAAG CCCCGGCTAA
RicCono4  GCGAAAGCCT GATCCAGCAA TACCGAGTGA GTGATGAAGG CCTTAGGGTT GTAAAGCTCT TTT-AGCAAG GAAGATAATG ACGTTACTTG -CAGAAAAAG CCCCGGCTAA
RicEmpPa  GCGAAAGCCT GATCCAGCAA TACCGAGTGA GTGATGAAGG CCCTAGGGTT GTAAAGCTCT TTT-AGCAAG GAAGATAATG ACGTTACTTG -CAGAAAAAG CCCCGGCTAA
RicJapon  GCGAAAGCCT GATCCAGCAA TACCGAGTGA GTGATGAAGG CCTTAGGGTT GTAAAGCTCT TTT-AGCAAG GAAGATAATG ACGTTACTTG -CAGAAAAAG CCCCGGCTAA
RicCana2  GCGAAAGCCT GATCCAGCAA TACCGAGTGA GTGATGAAGG CCTTAGGGTT GTAAAGCTCT TTT-AGCAAG GAAGATAATG ACGTTACTTG -CAGAAAAAG CCCCGGCTAA
RicHelve  GCGAAAGCCT GATCCAGCAA TACCGAGTGA GTGATGAAGG CCTTAGGGTT GTAAAGCTCT TTT-AGCAAG GAAGATAATG ACGTTACTTG -CAGAAAAAG CCCCGGCTAA
RicAust3  GCGAAAGCCT GATCCAGCAA TACCGAGTGA GTGATGAAGG CCCTAGGGTT GTAAAGCTCT TTT-AGCAAG GAAGATAATG ACGTTACTTG -CAGAAAAAG CCCCGGCTAA
RicBell3  GCGAAAGCCT GATCCAGCAA TACCGAGTGA GTGATGAAGG CCCTAGGGTT GTAAAGCTCT TTT-AGCAAG GAAGATAATG ACGTTACTTG -CAGAAAAAG CCCCGGCTAA
RicAkar3  GCGAAAGCCT GATCCAGCAA TACCGAGTGA GTGATGAAGG CCCTAGGGTT GTAAAGCTCT TTT-AGCAAG GAAGATAATG ACGTTACTTG -CAGAAAAAG CCCCGGCTAA
TorTsuku  GCGAAAGCCT GATCCAGCAA TACCGAGTGG GTGACGAAGG CCTTAGGGTT GTAAAGCCCT TTTCAGCAGG GAAGATAATG ACGGTACCTG ACCAAGAAAG CCCCGGCTAA
TorTagoi  GCGAAAGCCT GATCCAGCAA TACCGAGTGG GTGACGAAGG CCTTAGGGTT GTAAAGCCCT TTTCAGCAGG GAAGATAATG ACGGTACCTG ACCAAGAAAG CCCCGGCTAA
HemMarg2  GCGAAAGCCT GATCCAGCAA TACCGAGTGG GTGACGAAGG CCTTAGAGTT GTAAAGCCCT TTTCAGCAGG GAAGATAATG ACGGTACCTG ACCAAGAAAG CCCCGGCTAA
HydRick1  GCGAAAGCCT GATCCAGCAA TACCGCGTGA ATGATGAAGG CCTTAGGGTT GTAAAGTTCT TTT-AGTTGG GAAGATAATG ACGGTACCAA -CAGAAAAAG CCCCGGCTAA
IchMulti  GCGAAAGCCT GATCCAGCAA TACCGCGTGA ATGATGAAGG CCTTAGGGTT GTAAAGTTCT TTT-AGTAGG GAAGATAATG ACGGTACCTA -CAGAAAAAG CCCCGGCTAA
BryRick1  GCGAAAGCCT GATCCAGCAA TACCGCGTGA ATGATGAAGG CCCTAGGGTT GTAAAGTTCT TTT-AATAGG GAAGATAATG ACGGTACTTA -TAGAAAAAG CCCCGGCTAA
BryRick2  GCGAAAGCCT GATCCAGCAA TACCGCGTGA ATGATGAAGG CCTTAGGGTT GTAAAGTTCT TTT-AATAGG GAAGATAATG ACGGTACCTA -TAGAAAAAG CCCCGGCTAA
BryRick3  GCGAAAGCCT GATCCAGCAA TACCGCGTGA ATGATGAAGG CCCTAGGGTT GTAAAGTTCT TTT-AATAGG GGAGATAATG ACGGTACCTA -TAGAAAAAG CCCCGGCTAA
MonFaveo  GCGAAAGCCT GACCCAGCAA TATCGCGTGA ATGATGAAGG CCCTAGGGTT GTAAAGTTCT TTT-AATAGG GAAGATAATG ACGGTACCTA -TAGAAAAAG CCCCGGCTAA
RicNepCi  GCGAAAGCCT GATCCAGCAA TACCGAGTGG GTGACGAAGG CCTTAGGGTT GTAAAGCCCT TTTCAGCAGG GAAGATAATG ACGGTACCTG ACCAAGAAAG CCCCGGCTAA
Kalahari  GCGAAAGCCT GATCCAGCAA TACCGCGTGA ATGATGAAGG CCTTAGGGTT GTAAAGTTCT TTT-AGTTGG AAAGATAATG ACGGTACCAA -CAGAAAAAG CCCCGGCTAA
T6025C27  GCGAAAGCCT GATCCAGCAA TACCGCGTGA ATGATGAAGG CCTTAGGGTT GTAAAGTTCT TTT-AGCAGG GAAGATAATG ACGGTACCTG -CAGAAAAAG CCCCGGCTAA
T6025C01  GCGAAAGCCT GATCCAGCAA TACCGCGTGA ATGATGAAGG CCTTAGGGTT GTAAAGTTCT TTT-AGTTGG AAAGATAATG ACGGTACCAA -CAGAAAAAG CCCCGGCTAA
WakWat77  GCGAAAGCCT GATCCAGCAA TACCGCGTGA ATGATGAAGG CCTTAGGGTT GTAAAGTTCT TTT-AGTTGG GAAGATAATG ACGGTACCAA -CAGAAAAAG CCCCGGCTAA
Waste214  GCGAAAGCCT GATCCAGCAA TACCGCGTGA ATGATGAAGG CCTTAGGGTT GTAAAGTTCT TTT-AGTTGG AAAGATAATG ACGGTACCAA -CAGAAAAAG CCCCGGCTAA
TaihAS94  GCGAAAGCCT GATCCAGCAA TACCGCGTGA ATGATGAAGG CCTTAGGGTT GTAAAGTTCT TTT-AGTTGG AAAGATAATG ACGGTACCAA -CAGAAAAAG CCCCGGCTAA
HydRick2  GCGAAAGC-T GATCCAGCAA TACCGCGTGA ATGATGAAGG CCTTAGGGTT GTAAAGTTCT TTT-AGTTGG GAAGATAATG ACGGTACCAA -CAGAAAAAG CCCCGGCTAA
DioOBOD9  GCGAAAGCCT GATCCAGCAA TACCGCGTGA ATGATGAAGG CCTTAGGGTT GTAAAGTTCT TTT-AGTTGG AAAGATAATG ACGGTACCAA -CAGAAAAAG CCCCGGCTAA
PrmCauda  GCGAAAGCCT GATCCAGCAA TGCCGCGTGA ATGATGAAGG CCTTAGGGTT GTAAAGTTCT TTT-AGTTGG AAAGATAATG ACGGTACCAA -CAGAAAAAG CCCCGGCTAA
Sprsmm01  GCGAAAGCCT GATCCAGCAA TACCGCGTGA ATGATGAAGG CCTTAGGGTT GTAAAGTTCT TTT-AGTTGG AAAGATAATG ACGGTACCAA -CAGAAAAAG CCCCGGCTAA
DioDS124  GCGAAAGCCT GATCCAGCAA TACCGCGTGA ATGATGAAGG CCTTAGGGTT GTAAAGTTCT TTT-AGTTGG AAAGATAATG ACGGTACCAA -CAGAAAAAG CCCCGGCTAA
EupOct01  GCGAAAGCCT GATCCAGCAA TACCGCGTGA ATGATGAAGG CCTTAGGGTT GTAAAGTTCT TTT-AGTTGG AAAGATAATG ACGGTACCAA -CAGAAAAAG CCCCGGCTAA
NIES_425  GCGAAAGCCT GATCCAGCAA TACCGCGTGA ATGATGAAGG CCTTAGGGTT GTAAAGTTCT TTT-AGTTGG AAAGATAATG ACGGTACCAA -CAGAAAAAG CCCCGGCTAA
NIES_577  GCGAAAGCCT GATCCAGCAA TACCGCGTGA ATGATGAAGG CCTTAGGGTT GTAAAGTTCT TTT-AGTTGG AAAGATAATG ACGGTACCAA -CAGAAAAAG CCCCGGCTAA
UTEX2180  GCGAAAGCCT GATCCAGCAA TACCGCGTGA ATGATGAAGG CCTTAGGGTT GTAAAGTTCT TTT-AGTTGG AAAGATAATG ACGGTACCAA -CAGAAAAAG CCCCGGCTAA
EVE_____  GCGAAAGCCT GATCCAGCAA TACCG----- ---------- ---------- ---------- ---------- ---------- ---------- ---------- ----------
NIES_732  ---------- ---------- ---------- ---------- ---------- ---------- ---------- ---------- ---------- ---------- ----------
OrnTsIke  GCGAAAGCCT GATCCAGCAA TGCCGCGTGA GTGATGAAGG CCTTAGGGTT GTAAAGCTCT TTT-AGTAGG GATGATAATG ACAGTACCTA -CAGAAAAAG CCCCGGCTAA
OrnTsBor  GCGAAAGCCT GATCCAGCAA TGCCGCGTGA GTGATGAAGG CCTTAGGGTT GTAAAGCTCT TTT-AGTAGG GATGATAATG ACAGTACCTA -CAGAAAAAG CCCCGGCTAA
CprPolyt  GCGAAAGCCT GATCCAGCAA TGCCGCGTGA GTGATGAAGG CCGTAAGGTT GTAAAGCTCT TTC-AGCAGG AAAGATAATG ACGGTACCTG -CAAAAGAAG CCCCGGCTAA
```

```
          441
RicRicIo  CTCCGTGCCA GCAGCCGCGG TAAGACGGAG GGGGCTAGCG TTGTTCG-GA ATTACTGGGC GTAAAGAGTG CGTAGGCGGT TTAGTAAGTT GGAAGTGAAA GCCCGGGGCT
RicLimo2  CTCCGTGCCA GCAGCCGCGG TAAGACGGAG GGGGCTAGCG TTGTTCG-GA ATTACTGGGC GTAAAGAGTG CGTAGGCGGT TTAGTAAGTT GGAAGTGAAA GCCCGAGGCT
RicFeli2  CTCCGTGCCA GCAGCCGCGG TAAGACGGAG GGGGCTAGCG TTGTTCG-GA ATTACTGGGC GTAAAGAGTG CGTAGGCGGT TTAGTAAGTT GGAAGTGAAA GCCCGGGGCT
RicProwa  CTCCGTGCCA GCAGCCGCGG TAAGACGGAG GGGGCTAGCG TTGTTCG-GA ATTACTGGGC GTAAAGAGTG CGTAGGCGGT TTAGTAAGTT GGAAGTGAAA GCCCGGGGCT
RicTyphi  CTCCGTGCCA GCAGCCGCGG TAAGACGGAG GGGGCTAGCG TTGTTCG-GA ATTACTGGGC GTAAAGAGTG CGTAGGTGGT TTAGTAAGTT GGAAGTGAAA GCCCGGGGCT
RicCono4  CTCCGTGCCA GCAGCCGCGG TAAGACGGAG GGGGCTAGCG TTGTTCG-GA ATTACTGGGC GTAAAGAGTG CGTAGGCGGT TTAGTAAGTT GGAAGTGAAA GCCCGGGGCT
RicEmpPa  CTCCGTGCCA GCAGCCGCGG TAAGACGGAG GGGGCTAGCG TTGTTCG-GA ATTACTGGGC GTAAAGAGTG CGTAGGCGGT TTAGTAAGTT GGAAGTGAAA GCCCGGGGCT
RicJapon  CTCCGTGCCA GCAGCCGCGG TAAGACGGAG GGGGCTAGCG TTGTTCG-GA ATTACTGGGC GTAAAGAGTG CGTAGGCGGT TTAGTAAGTT GGAAGTGAAA GCCCGGGGCT
RicCana2  CTCCGTGCCA GCAGCCGCGG TAAGACGGAG GGGGCTAGCG TTGTTCG-GA ATTACTGGGC GTAAAGAGTG CGTAGGCGGT TTAGTAAGTT GGAAGTGAAA GCCCGGGGCT
RicHelve  CTCCGTGCCA GCAGCCGCGG TAAGACGGAG GGGGCTAGCG TTGTTCG-GA ATTACTGGGC GTAAAGAGTG CGTAGGCGGT TTAGTAAGTT GGAAGTGAAA GCCCGGGGCT
RicAust3  CTCCGTGCCA GCAGCCGCGG TAAGACGGAG GGGGCTAGCG TTGTTCG-GA ATTACTGGGC GTAAAGAGTG CGTAGGCGGT TTAGTAAGTT GGAAGTGAAA GCCCGGGGCT
RicBell3  CTCCGTGCCA GCAGCCGCGG TAAGACGGAG GGGGCTAGCG TTGTTCG-GA ATTACTGGGC GTAAAGAGTG CGTAGGCGGT TTAGTAAGTT GGAAGTGAAA GCCCGGGGCT
RicAkar3  CTCCGTGCCA GCAGCCGCGG TAAGACGGAG GGGGCTAGCG TTGTTCG-GA ATTACTGGGC GTAAAGAGTG CGTAGGCGGT TTAGTAAGTT GGAAGTGAAA GCCCGGGGCT
TorTsuku  CTCCGTGCCA GCAGCCGCGG TAAGACGGAG GGGGCTAGCG TTGTTCG-GA ATTACTGGGC GTAAAGAGTG CGTAGGCGGT TTAGTAAGTT GGAAGTGAAA GCCCGAGGCT
TorTagoi  CTCCGTGCCA GCAGCCGCGG TAAGACGGAG GGGGCTAGCG TTGTTCG-GA ATTACTGGGC GTAAAGAGTG CGTAGGCGGT TTAATAAGTT GGAAGTGAAA GCCCGAGGCT
HemMarg2  CTCCGTGCCA GCAGCCGCGG TAAGACGGAG GGGGCTAGCG TTGTTCG-GA ATTACTGGGC GTAAAGAGTG CGTAGGCGGT TTAGTAAGTT GGAAGTGAAA GCCCGAGGCT
HydRick1  CTCCGTGCCA GCAGCCGCGG TAAGACGGAG GGGGCTAGCG TTGTTCG-GA ATTACTGGGC GTAAAGGGCG CGTAGGCGGA TTAGTAAGTT GGGAGTGAAA GCCCGGGGCT
IchMulti  CTCCGTGCCA GCAGCCGCGG TAAGACGGAG GGGGCTAGCG TTGTTCG-GA ATTACTGGGC GTAAAGGGCG CGTAGGCGGA TTAGTAAGTT GGAAGTGAAA GCCCGGGGCT
BryRick1  CTCCGTGCCA GCAGCCGCGG TAAGACGGAG GGGGCTAGCG TTATTCG-GA ATTACTGGGC GTAAAGGGCG CGTAGGCGGA TTAGTAAGTT GGGAGTGAAA GCCCGGGGCT
BryRick2  CTCCGTGCCA GCAGCCGCGG TAAGACGGAG GGGGCTAGCG TTGTTCG-GA ATTACTGGGC GTAAAGGGCG CGTAGGCGGA TTAGTAAGTT GGGAGTGAAA GCCCGGGGCT
BryRick3  CTCCGTGCCA GCAGCCGCGG TAAGACGGAG GGGGCTAGCG TTATTCG-GA ATTACTGGGC GTAAAGGGCG CGTAGGCGGA TTAGTAAGTT GGGAGTGAAA GCCCGGGGCT
MonFaveo  CTCCGTGCCA GCAGCCGCGG TAAGGCGGAG GGGGCTAGCG TTATTCG-GA ATTACTGGGC GTAAAGGGCG CGTAGGCGGA TTAGTAAGTT GGGAGTGAAA GCCCGGGGCT
RicNepCi  CTCCGTGCCA GCAGCCGCGG TAAGACGGAG GGGGCTAGCG TTGTTCG-GA ATTACTGGGC GTAAAGAGTG CGTAGGCGGT TTAGTAAGTT GGAAGTGAAA GCCCGAGGCT
Kalahari  CTCCGTGCCA GCAGCCGCGG TAAGACGGAG GGGGCTAGCG TTGTTCG-GA ATTACTGGGC GTAAAGGGCG CGTAGGCGGA TTAGTAAGTT GGGAGTGAAA GCCCGGGGCT
T6025C27  CTCCGTGCCA GCAGCCGCGG TAAGACGGAG GGGGCTAGCG TTGTTCG-GA ATTACTGGGC GTAAAGGGCG CGTAGGCGGA TTAGTAAGTT GGAAGTGAAA GCCCGGGGCT
T6025C01  CTCCGTGCCA GCAGTCGCGG TAAGACGGAG GGGGCTAGCG TTGTTCG-GA ATTACTGGGC GTAAAGGGCG CGTAGGCGGA TTAGTAAGTT GGGAGTGAAA GCCCGGGGCT
WakWat77  CTCCGTGCCA GCAGCCGCGG TAAGACGGAG GGGGCTAGCG TTGTTCG-GA ATTACTGGGC GTAAAGGGCG CGTAGGCGGA TTAGTAAGTT GGGAGTGAAA GCCCGGGGCT
Waste214  CTCCGTGCCA GCAGCCGCGG TAAGACGGAG GGGGCTAGCG TTGTTCG-GA ATTACTGGGC GTAAAGGGCG CGTAGGCGGA TTAGTAAGTT GGGGGTGAAA GCCCGGGGCT
TaihAS94  CTCCGTGCCA GCAGCCGCGG TAAGACGGAG GGGGTTAGCG TTGTTCG-GA ATTACTGGGC GTAAAGGGCG CGTAGGCGGA TTAGTAAGTT GGGAGTGAAA GCCCGGGGCT
HydRick2  CTCCGTGCCA GCAGCCGCGG TAAGACGGAG GGGGCTAGCG TTGTTCG-GA ATTACTGGGC GTAAAGGGCG CGTAGGCGGA TTAGTAAGTT GGGAGTGAAA GCCCGGGGCT
DioOBOD9  CTCCGTGCCA GCAGCCGCGG TAAGACGGAG GGGGCTAGCG TTGTTCGGAT ATTACTGGGC GTAAAGGGCG CGTAGGCGGA TTAGTAAGTT GGGAGTGAAA GCCCGGGGCT
PrmCauda  CTCCGTGCCA GCAGCCGCGG TAAGACGGAG GGGGCTAGCG TTGTTCG-GA ATTACTGGGC GTAAAGGGCG CGTAGGCGGA TTAGTAAGTT GGGAGTGAAA GCCCGGGGCT
Sprsmm01  CTCCGTGCCA GCAGCCGCGG TAAGACGGAG GGGGCTAGCG TTGTTCG-GA ATTACTGGGC GTAAAGGGCG CGTAGGCGGA TTAGTAAGTT GGGAGTGAAA GCCCGGGGCT
DioDS124  CTCCGTGCCA GCAGCCGCGG TAAGACGGAG GGGGCTAGCG TTGTTCG-GA ATTACTGGGC GTAAAGGGCG CGTAGGCGGA TTAGTAAGTT GGGAGTGAAA GCCCGGGGCT
EupOct01  CTCCGTGCCA GCAGCCGCGG TAAGACGGAG GGGGCTAGCG TTGTTCG-GA ATTACTGGGC GTAAAGGGCG CGTAGGCGGA TTAGTAAGTT GGGAGTGAAA GCCCGGGGCT
NIES_425  CTCCGTGCCA GCAGCCGCGG TAAGACGGAG GGGGCTAGCG TTGTTCG-GA ATTACTGGGC GTAAAGGGCG CGTAGGCGGA TTAGTAAGTT GGGAGTGAAA GCCCGGGGCT
NIES_577  CTCCGTGCCA GCAGCCGCGG TAAGACGGAG GGGGCTAGCG TTGTTCG-GA ATTACTGGGC GTAAAGAGCG CGTAGGCGGA TTAGTAAGTT GGGAGTGAAA GCCCGGGGCT
UTEX2180  CTCCGTGCCA GCAGCCGCGG TAAGACGGAG GGGGCTAGCG TTGTTCG-GA ATTACTGGGC GTAAAGGGCG CGTAGGCGGA TTAGTAAGTT GGGAGTGAAA GCCCGGGGCT
EVE_____  ---------- ---------- ---------- ---------- ---------- ---------- ---------- ---------- ---------- ---------- ----------
NIES_732  ---------- ---------- ---------- ---------- ---------- ---------- ---------- ---------- ---------- --GAGTGAAA GCCCGGGGCT
OrnTsIke  CTCCGTGCCA GCAGCCGCGG TAAGACGGAG GGGGCTAGCG TTGTTCG-GA ATTACTGGGC GTAAAGGGCG CGTAGGCGGT TTAATAAGTT AGGAGTGAAA TCCCGGGGCT
OrnTsBor  CTCCGTGCCA GCAGCCGCGG TAAGACGGAG GGGGCTAGCG TTGTTCG-GA ATTACTGGGC GTAAAGGGCG CGTAGGCGGT TTAATAAGTT AGGAGTGAAA TCCCGGGGCT
CprPolyt  CTCCGTGCCA GCAGCCGCGG TAAGACGGAG GGGGCTAGCG TTGTTCG-GA ATTACTGGGC GTAAAGCGCA TGTAGGCGGT TTAGTAAGTT GGAAGTGAAA GCCCAGGGCT
```

```
           551
RicRicIo   TAACCTCGGA ATTGCTTTCA AAACTACTAA TCTAGAGTGT AGTAGGGGAT GATGGAATTC CTAGTGTAGA GGTGAAATTC TTAGATATTA GGAGGAACAC CGGTGGCGAA
RicLimo2   TAACCTCGGA ACTGCTTTCA AAACTACTAA TCTAGAGTGT AGTAGGGGAT GATGGAATTC CTAGTGTAGA GGTGAAATTC TTAGATATTA GGAGGAACAC CGGTGGCGAA
RicFeli2   TAACCTCGGA ATTGCTTTCA AAACTACTAA TCTAGAGTGT AGTAGGGGAT GATGGAATTC CTAGTGTAGA GGTGAAATTC TTAGATATTA GGAGGAACAC CGGTGGCGAA
RicProwa   TAACCTCGGA ATTGCTTTCA AAACTACTAA TCTAGAGTGT AGTAGGGGAT GATGGAATTC CTAGTGTAGA GGTGAAATTC TTAGATATTA GGAGGAACAC CGGTGGCGAA
RicTyphi   TAACCTCGGA ATTGCTTTCA AAACTACTAA TCTAGAGTGT AGTAGGGGAT GATGGAATTC CTAGTGTAGA GGTGAAATTC TTAGATATTA GGAGGAACAC CGGTGGCGAA
RicCono4   TAACCTCGGA ATTGCTTTCA AAACTACTAA TCTAGAGTGT AGTAGGGGAT GATGGAATTC CTAGTGTAGA GGTGAAATTC TTAGATATTA GGAGGAACAC CGGTGGCGAA
RicEmpPa   TAACCTCGGA ATTGCTTTCA AAACTACTAA TCTAGAGTGT AGTAGGGGAT GATGGAATTC CTAGTGTAGA GGTGAAATTC TTAGATATTA GGAGGAACAC CGGTGGCGAA
RicJapon   TAACCTCGGA ATTGCTTTCA AAACTACTAA TCTAGAGTGT AGTAGGGGAT GATGGAATTC CTAGTGTAGA GGTGAAATTC TTAGATATTA GGAGGAACAC CGGTGGCGAA
RicCana2   TAACCTCGGA ATTGCTTTCA AAACTACTAA TCTAGAGTGT AGTAGGGGAT GATGGAATTC CTAGTGTAGA GGTGAAATTC TTAGATATTA GGAGGAACAC CGGTGGCGAA
RicHelve   TAACCTCGGA ATTGCTTTCA AAACTACTAA TCTAGAGTGT AGTAGGGGAT GATGGAATTC CTAGTGTAGA GGTGAAATTC TTAGATATTA GGAGGAACAC CGGTGGCGAA
RicAust3   TAACCTCGGA ATTGCTTTCA AAACTACTAA TCTAGAGTGT AGTAGGGGAT GATGGAATTC CTAGTGTAGA GGTGAAATTC TTAGATATTA GGAGGAACAC CGGTGGCGAA
RicBell3   TAACCTCGGA ATTGCTTTCA AAACTACTAA TCTAGAGTGT AGTAGGGGAT GATGGAATTC CTAGTGTAGA GGTGAAATTC TTAGATATTA GGAGGAACAC CGGTGGCGAA
RicAkar3   TAACCTCGGA ATTGCTTTCA AAACTACTAA TCTAGAGTGT AGTAGGGGAT GATGGAATTC CTAGTGTAGA GGTGAAATTC TTAGATATTA GGAGGAACAC CGGTGGCGAA
TorTsuku   TAACCTCGGA ACTGCTTTCA AAACTACTAA TCTAGAGTGT AGTAGGGGAT GATGGAATTC CTAGTGTAGA GGTGAAATTC TTAGATATTA GGAGGAACAC CGGTGGCGAA
TorTagoi   TAACCTCGGA ACTGCTTTCA AAACTACTAA TCTAGAGTGT AGTAGGGGAT GATGGAATTC CTAGTGTAGA GGTGAAATTC TTAGATATTA GGAGGAACAC CGGTGGCGAA
HemMarg2   TAACCTCGGA ACTGCTTTCA AAACTACTAA TCTAGAGTGT AGTAGGGGAT GATGGAATTC CTAGTGTAGA GGTGAAATTC TTAGATATTA GGAGGAACAC CGGTGGCGAA
HydRick1   TAACCTCGGA ACTGCTTTCA AAACTGCTAG TCTAGAGTGA AGTAGGGGGT GATGGAATTC CTAGTGTAGA GGTGAAATTC TTAGATATTA GGAGGAACAC CGGTGGCGAA
IchMulti   TAACCTCGGA ACTGCTTTCA AAACTGCTAG TCTAGAGTGA AGTAGGGGAT GATGGAATTC CTAGTGTAGA GGTGAA-TTC TTAGATATTA GGAGGAACAC CGGTGGCGAA
BryRick1   AAACCTCGGA ACTGCTTTCA AAACTGCTAG TCTTGAGTGA AGTAGGGGGT GATGGAATTC CTAGTGTAGA GGTGAAATTC TTAGATATTA GGAGGAACAC CGGTGGCGAA
BryRick2   TAACCTCGGA ACTGCTTTCA AAACTGCTAG TCTTGAGTGA AGTAGGGGGT GATGGAATTC CTAGTGTAGA GGTGAAATTC TTAGATATTA GGAGGAACAC CGGTGGCGAA
BryRick3   TAACCTCGGA ACTGCTTTCA AAACTGCTAG TCTTGAGTGA AGTAGGGGGT GATGGAATTC CTAGTGTAGA GGTGAAATTC TTAGATATTA GGAGGAACAC CGGTGGCGAA
MonFaveo   TAACCTCGGA ACTGCTTTCA AAACTGCTAG TCTTGAGTGA AGTAGGGGGT GATGGAATTC CTAGTGTAGA GGTGAAATTC TTAGATATTA GGAGGAACAC CGGTGGCGAA
RicNepCi   TAACCTCGGA ACTGCTTTCA AAACTACTAA TCTAGAGTGT AGTAGGGGAT GATGGAATTC CTAGTGTAGA GGTGAAATTC TTAGATATTA GGAGGAACAC CGGTGGCGAA
Kalahari   TAACCTCGGA ACTGCTTTCA AAACTGCTAG TCTTGAGTAA AGTAGGGGAT GATGGAATTC CTAGTGTAGA GGTGAAATTC TTAGATATTA GGAGGAACAC CGGTGGCGAA
T6025C27   TAACCTCGGA ACTGCTTTCA AAACTGCTAG TCTTGAGTGA AGCAGGGGAT GATGGAATTC CTAGTGTAGA GGTGAAATTC TCAGATATTA GGAGGAACAC CGGTGGCGAA
T6025C01   TAACCTCGGA ACTGCTTTCA AAACTGCTAG TCTTGAGTGA AGTAGGGGAT GATGGAATTC CTAGTGTAGA GGTGAAATTC TTAGATATTA GGAGGAACAC CGGTGGCGAA
WakWat77   TAACCTCGGA ACTGCTTTCA AA-CTGCTAG TCTTGAGTGA AGTAGGGGAT GATGGAATTC CTAGTGTAGA GGTGAAATTC TTAGATATTA GGAGGAACAC CGGTGGCGAA
Waste214   TAACCTCGGA ACTGCTTTCA AAACTGCTAG TCTTGAGTGA AGTAGGGGAT GATGGAATTC CTAGTGTAGA GGTGAAATTC TTAGATATTA GGAGGAACAC CGGTGGCGAA
TaihAS94   TAACCTCGGA ACTGCTTTCA AAACTGCTAG TCTTGAGTGA AGTAGGGGAT GATGGAATTC CTAGTGTAGA GGTGAAATTC TTAGATATTA GGAGGAACAC CGGTGGCGAA
HydRick2   TAACCTCGGA ACTGCTTTCA AAACTGCTAG TCTAGAGTGA AGTAGGGGGT GATGGAATTC CTAGTGTAGA GGTGAAATTC TTAGATATTA GGAGGAACAC CGGTGGCGAA
DioOBOD9   TAACCTCGGA ACTGCTTTCA AAACTGCTAG TCTTGAGTGA AGTAGGGGAT GATGGAATTC CTAGTGTAGA GGTGAAATTC TTAGATATTA GGAGGAACAC CGGTGGCGAA
PrmCauda   TAACCTCGGA ACTGCTTTCA AAACTGCTAG TCTTGAGTGA AGTAGGGGAT GATGGAATTC CTAGTGTAGA GGTGAAATTC TTAGATATTA GGAGGAACAC CGGTGGCGAA
Sprsmm01   TAACCTCGGA ACTGCTTTCA AAACTGCTAG TCTTGAGTGA AGTAGGGGAT GATGGAATTC CTAGTGTAGA GGTGAAATTC TTAGATATTA GGAGGAACAC CGGTGGCGAA
DioDS124   TAACCTCGGA ACTGCTTTCA AAACTGCTAG TCTTGAGTGA AGTAGGGGAT GATGGAATTC CTAGTGTAGA GGTGAAATTC TTAGATATTA GGAGGAACAC CGGTGGCGAA
EupOct01   TAACCTCGGA ACTGCTTTCA AAACTGCTAG TCTTGAGTGA AGTAGGGGAT GATGGAATTC CTAGTGTAGA GGTGAAATTC TTAGATATTA GGAGGAACAC CGGTGGCGAA
NIES_425   TAACCTCGGA ACTGCTTTCA AAACTGCTAG TCTTGAGTGA AGTAGGGGAT GATGGAATTC CTAGTGTAGA GGTGAAATTC TTAGATATTA GGAGGAACAC CGGTGGCGAA
NIES_577   TAACCTCGGA ACTGCTTTCA AAACTGCTAG TCTTGAGTGA AGTAGGAGAT GATGGAATTC CTAGTGTAGA GGTGAAATTC TTAGATATTA GGAGGAACAC CGGTGGCGAA
UTEX2180   TAACCTCGGA ACTGCTTTCA AAACTGCTAG TCTTGAGTGA AGTAGGGGAT GATGGAATTC CTAGTGTAGA GGTGAAATTC TTAGATATTA GGAGGAACAC CGGTGGCGAA
EVE_____   ---------- ---------- ---------- ---------- ---------- ---------- ---------- ---------- ---------- ---------- ----------
NIES_732   TAACCTCGGA ACTGCTTTCA AAACTGCTAG TCTTGAGTGA AGTAGGGGAT GATGGAATTC CTAGAGTAGA GGTGAAATTA --GGG--CT- GGAGGAACAA CGGTGGCGAT
OrnTsIke   TAACCCTGGA ACTGCTTCTA AAACTGTTAG GCTAGAGTGT GGTAGAGGAT GATGGAATTT CTAGTGTAGA GGTAAAATTC TTAGATATTA GAAGGAACAC CAGTGGCGAA
OrnTsBor   TAACCCTGGA ACTGCTTCTA AAACTGTTAG GCTAGAGTAT GGTAGAGGAT GATGGAATTT CTAGTGTAGA GGTAAAATTC TTAGATATTA GAAGGAACAC CAGTGGCGAA
CprPolyt   CAACCCTGGA ACTGCTTTCA AAACTGCTAA GCTAGAGTGC GGAAGGGGGT GATGGAATTC CTAGTGTAGA GGTGAAATTC ATAGATATTA GGAGGAACAC CAAAGGCGAA
```

```
        661
RicRicIo  GGCGGTCATC TGGGCTACCA CTGACGCTGA TGCACGAAAG CGTGGGGAGC AAACAGGATT AGATACCCTG GTAGTCCACG CCGTAAACGA TGAGTGCTAG ATATCGGAAG
RicLimo2  GGCGGTCATC TAGGCTACAA CTGACGCTGA TGCACGAAAG CGTGGGGAGC AAACAGGATT AGATACCCTG GTAGTCCACG CCGTAAACGA TGAGTGCTAG ATATCGGGAG
RicFeli2  GGCGGTCATC TGGGCTACAA CTGACGCTGA TGCACGAAAG CGTGGGGAGC AAACAGGATT AGATACCCTG GTAGTCCACG CCGTAAACGA TGAGTGCTAG ATATCGGAAG
RicProwa  GGCGGTCATC TGGGCTACAA CTGACGCTGA TGCACGAAAG CGTGGGGAGC AAACAGGATT AGATACCCTG GTAGTCCACG CCGTAAACGA TGAGTGCTAG ATATCGGAGG
RicTyphi  GGCGGTCATC TGGGCTACAA CTGACGCTGA TGCACGAAAG CGTGGGGAGC AAACAGGATT AGATACCCTG GTAGTCCACG CCGTAAACGA TGAGTGCTAG ATATCGGAGG
RicCono4  GGCGGTCATC TGGGCTACAA CTGACGCTGA TGCACGAAAG CGTGGGGAGC AAACAGGATT AGATACCCTG GTAGTCCACG CCGTAAACGA TGAGTGCTAG ATATCGGAAG
RicEmpPa  GGCGGTCATC TGGGCTACAA CTGACGCTGA TGCACGAAAG CGTGGGGAGC AAACAGGATT AGATACCCTG GTAGTCCACG CCGTAAACGA TGAGTGCTAG ATATTGGGAG
RicJapon  GGCGATCATC TGGGCTACAA CTGACGCTGA TGCACGAAAG CGTGGGGAGC AAACAGGATT AGATACCCTG GTAGTCCACG CCGTAAACGA TGAGTGCTAG ATATCGGAAG
RicCana2  GGCGATCATC TAGGCTATAA CTGACGCTGA TGCACGAAAG CGTGGGGAGC AAACAGGATT AGATACCCTG GTAGTCCACG CCGTAAACGA TGAGTGCTAG ATATCGGGAG
RicHelve  GGCGATCATC TGGGCTACAA CTGACGCTGA TGCACGAAAG CGTGGGGAGC AAACAGGATT AGATACCCTG GTAGTCCACG CCGTAAACGA TGAGTGCTAG ATATCGGAAG
RicAust3  GGCGGTCATC TGGGCTACAA CTGACGCTGA TGCACGAAAG CGTGGGGAGC AAACAGGATT AGATACCCTG GTAGTCCACG CCGTAAACGA TGAGTGCTAG ATATCGGAAG
RicBell3  GGCGGTCATC TGGGCTACAA CTGACGCTGA TGCACGAAAG CGTGGGGAGC AAACAGGATT AGATACCCTG GTAGTCCACG CCGTAAACGA TGAGTGCTAG ATATTGGGAG
RicAkar3  GGCGGTCATC TGGGCTACGA CTGACGCTGA TGCACGAAAG CGTGGGGAGC AAACAGGATT AGATACCCTG GTAGTCCACG CCGTAAACGA TGAGTGCTAG ATATCGGAAG
TorTsuku  GGCGGTCATT TAGGCTACAA CTGACGCTGA TGCACGAAAG CGTGGGGAGC AAACAGGATT AGATACCCTG GTAGTCCACG CCGTAAACGA TGAGTGCTAG ATATCGGGAG
TorTagoi  GGCGGTCATT TAGGCTACAA CTGACGCTGA TGCACGAAAG CGTGGGGAGC AAACAGGATT AGATACCCTG GTAGTCCACG CCGTAAACGA TGAGTGCTAG ATATCGGGAG
HemMarg2  GGCGATCATA TAGGCTACAA CTGACGCTGA TACACGAAAG CGTGGGGAGC AAACAGGATT AGATACCCTG GTAGTCCACG CCGTAAACGA TGAGTGCTAG ATATCGGGAG
HydRick1  GGCGGTCACC TGGACTTCAA CTGACGCTGA GGCGCGAAAG CGTGGGGAGC AAACAGGATT AGATACCCTG GTAGTCCACG CTGTAAACGA TGAGTGCTAG ATGTCGGGAA
IchMulti  GGCGGTCATC TGGACTTCAA CTGACGCTGA GGCGCGAAAG CGTGGGGAGC AAACAGGATT AGATACCCTG GTAGTCCACG CCGTAAACGA TGAGTGCTAG ATGTCGGAAG
BryRick1  GGCGGTCACC TGGACTTCAA CTGACGCTGA GGCGCGAAAG CGTGGGGAGC AAACAGGATT AGATACCCTG GTAGTCCACG CCGTAAACGA TGAGTGCTAG ATATCGGGAA
BryRick2  GGCGGTCACC TGGACTTCAA CTGACGCTGA GGCGCGAAAG CGTGGGGAGC AAACAGGATT AGATACCCTG GTAGTCCATG CCGTAAACGA TGAGTGCTAG ATATCGGGAA
BryRick3  GGCGGTCACC TGGACTTCAA CTGACGCTGA GGCGCGAAAG CGTGGGGAGC AAACAGGATT AGATACCCTG GTAGTCCACG CCGTAAACGA TGAGTGCTAG ATATCGGGAA
MonFaveo  GGCGGTCACC TGGACTTCAA CTGACGCTGA GGCGCGAAAG CGTGGGGAGC AAACAGGATT AGATACCCTG GTAGTCCACG CCGTAAACGA TGAGTGCTAG ATATCGGGAA
RicNepCi  GGCGGTCATC TAGGCTACAA CTGACGCTGA TGCACGAAAG CGTGGGGAGC AAACAGGATT AGATACCCTG GTAGTCCACG CCGTAAACGA TGAGTGCTAG ATATCGGGAG
Kalahari  GGCGGTCATC TGGACTTCAA CTGACGCTGA GGCGCGAAAG CGTGGGGAGC AAACAGGATT AGATACCCTG GTAGTCCACG CCGTAAACGA TGAGTGCTAG ATATCGGGAG
T6025C27  GGCGGTCATC TGGACTTCAA CTGACGCTGA GGCGCGAAAG CGTGGGGAGC AAACAGGATT AGATACCCTG GTAGTCCACG CTGTAAACGA TGAGTGCTAG ATATCGGGAG
T6025C01  GGCGGTCATC TGGACTTCAA CTGACGCTGA GGCGCGAAAG CGTGGGGAGC AAACAGGATT AGATACCCTG GTAGTCCACG CCGTAAACGA TGAGTGCTAG ATATCGGGAG
WakWat77  GGCGGTCATC TGGACTTCAA CTGACGCTGA GGCGCGAAAG CGTGGGGAGC AAACAGGATT AGATACCCTG GTAGTCCACG CTGTAAACGA TGAGTGCTAG ATATCGGGAG
Waste214  GGCGGTCATC TGGACTTCAA CTGACGCTGA GGCGCGAAAG CGTGGGGAGC AAACAGGATT AGATACCCTG GTAGTCCACG CCGTAAACGA TGAGTGCTAG ATATCGGGAG
TaihAS94  GGCGGTCATC TGGACTTCAA CTGACGCTGA GGCGCGAAAG CGTGGGGAGC AAACAGGATT AGATACCCTG GTAGTCCACG CCGTAAACGA TGAGTGCTAG ATATCGGGAG
HydRick2  GGCGGTCACC TGGACTTCAA CTGACGCTGA GGCGCGAAAG GTGGGGGAGC AAACAGGATT AGATACCCTG GTAGTCCACG CTGTAAACGA TGAGTGCTAG ATGTCGGGAA
DioOBOD9  GGCGGTCATC TGGACTTCAA CTGACGCTGA GGCGCGAAAG CGTGGGGAGC AAACAGGATT AGATACCCTG GTAGTCCACG CCGTAAACGA TGAGTGCTAG ATATCGGGAG
PrmCauda  GGCGGTCATC TGGACTTCAA CTGACGCTGA GGCGCGAAAG CGTGGGGAGC AAACAGGATT AGATACCCTG GTAGTCCACG CCGTAAACGA TGAGTGCTAG ATATCGGGAG
Sprsmm01  GGCGGTCATC TGGACTTCAA CTGACGCTGA GGCGCGAAAG CGTGGGGAGC AAACAGGATT AGATACCCTG GTAGTCCACG CCGTAAACGA TGAGTGCTAG ATATCGGGAG
DioDS124  GGCGGTCATC TGGACTTCAA CTGACGCTGA GGCGCGAAGG CGTGGGGAGC AAACAGGATT AGATACCCTG GTAGTCCACG CCGTAAACGA TGAGTGCTAG ATATCGGGAG
EupOct01  GGCGGTCATC TGGACTTCAA CTGACGCTGA GGCGCGAAAG CGTGGGGAGC AAACAGGATT AGATACCCTG GTAGTCCACG CCGTAAACGA TGAGTGCTAG ATATCGGGAG
NIES_425  GGCGGTCATC TGGACTTCAA CTGACGCTGA GGCGCGAAAG CGTGGGGAGC AAACAGGATT AGATACCCTG GTAGTCCACG CCGTAAACGA TGAGTGCTAG ATATCGGGAG
NIES_577  GGCGGTCATC TGGACTTCAA CTGACGCTGA GGCGCGAAAG CGTGGGGAGC AAACAGGATT AGATACCCTG GTAGTCCACG CCGTAAACGA TGAGTGCTAG ATATCGGGAG
UTEX2180  GGCGGTCATC TGGACTTCAA CTGACGCTGA GGCGCGAAAG CGTGGGGAGC AAACAGGATT AGATACCCTG GTAGTCCACG CCGTAAACGA TGAGTGCTAG ATATCGGGAG
EVE_____  ---------- ---------- ---------- ---------- ---------- ---------- ---------- ---------- ---------- ---------- ----------
NIES_732  GGCGGTCATC TGGAATTCAA CTGACGCTGA GGCGCGAAAG CGTGGGGAGC AAACAGGATT AGATACCCTG GTAGTCCACG CCGTAAACGA TGAGTGCTAG ATATCGGGGT
OrnTsIke  AGCTGTCATC TGGGCCATTA CTGACGCTGA GGCGCGAAAG CGTGGGGAGC AAACAGGATT AGATACCCTG GTAGTCCACG CTGTAAACGA TGAGTGCTAG ATATTGGGGG
OrnTsBor  AGCTGTCATC TGGGCCATTA CTGACGCTGA GGCGCGAAAG CGTGGGGAGC AAACAGGATT AGATACCCTG GTAGTCCACG CTGTAAACGA TGAGTGCTAG ATATTGGGGG
CprPolyt  AGCATTCACC TGGTCCGCAA CTGACGCTGA GATGCGAAAG CGTGGGGAGC AAACAGGATT AGATACCCTG GTAGTCCACG CTGTAAACGA TGAGTGCTAG ATATCGGGAA
```

```
          771
RicRicIo  ATTC-TCTTT CGGTTTCGCA GCTAACGCAT TAAGCACTCC GCCTGGGGAG TACGGTCGCA AGATTAAAAC TCAAAGGAAT TGACGGGGGC TCGCACAAGC GGTGGAGCAT
RicLimo2  AAAT-TCTTT CGGTTTCGTA GCTAACGCAT TAAGCACTCC GCCTGGGGAG TACGGTCGCA AGATTAAAAC TCAAAGGAAT TGACGGGGGC TCGCACAAGC GGTGGAGCAT
RicFeli2  ATTC-TCTTT CGGTTTCGCA GCTAACGCAT TAAGCACTCC GCCTGGGGAG TACGGTCGCA AGATTAAAAC TCAAAGGAAT TGACGGGGGC TCGCACAAGC GGTGGAGCAT
RicProwa  ATTC-TCTTT CGGTTTCGCA GCTAACGCAT TAAGCACTCC GCCTGGGGAG TACGGTCGCA AGATTAAAAC TCAAAGGAAT TGACGGGGGC TCGCACAAGC GGTGGAGCAT
RicTyphi  ATTC-TCTTT CGGTTTCGCA GCTAACGCAT TAAGCACTCC GCCTGGGGAG TACGGTCGCA AGATTAAAAC TCAAAGGAAT TGACGGGGGC TCGCACAAGC GGTGGAGCAT
RicCono4  ATTC-TCTTT CGGTTTCGCA GCTAACGCAT TAAGCACTCC GCCTGGGGAG TACGGTCGCA AGATTAAAAC TCAAAGGAAT TGACGGGGGC TCGCACAAGC GGTGGAGCAT
RicEmpPa  ATTT-TCTCT CGGTTTCGCA GCTAACGCAT TAAGCACTCC GCCTGGGGAG TACGGTCGCA AGATTAAAAC TCAAAGGAAT TGACGGGGGC TCGCACAAGC GGTGGAGCAT
RicJapon  ATTC-TCTTT CGGTTTCGCA GCTAACGCAT TAAGCACTCC GCCTGGGGAG TACGGTCGCA AGATTAAAAC TCAAAGGAAT TGACGGGGGC TCGCACAAGC GGTGGAGCAT
RicCana2  AATA-TCTCT CGGTTTCGCA GCTAACGCAT TAAGCACTCC GCCTGGGGAG TACGGTCGCA AGATTAAAAC TCAAAGGAAT TGACGGGGGC TCGCACAAGC GGTGGAGCAT
RicHelve  ATTC-TCTTT CGGTTTCGCA GCTAACGCAT TAAGCACTCC GCCTGGGGAG TACGGTCGCA AGATTAAAAC TCAAAGGAAT TGACGGGGGC TCGCACAAGC GGTGGAGCAT
RicAust3  ATTT-TCTTT CGGTTTCGCA GCTAACGCAT TAAGCACTCC GCCTGGGGAG TACGGTCGCA AGATTAAAAC TCAAAGGAAT TGACGGGGGC TCGCACAAGC GGTGGAGCAT
RicBell3  ATTT-TCTCT CGGTTTCGCA GCTAACGCAT TAAGCACTCC GCCTGGGGAG TACGGTCGCA AGATTAAAAC TCAAAGGAAT TGACGGGGGC TCGCACAAGC GGTGGAGCAT
RicAkar3  AGTC-TCTTT CGGTTTCGCA GCTAACGCAT TAAGCACTCC GCCTGGGGAG TACGGTCGCA AGATTAAAAC TCAAAGGAAT TGACGGGGGC TCGCACAAGC GGTGGAGCAT
TorTsuku  AATT-TCTTT CGGTTTCGTA GCTAACGCAT TAAGCACTCC GCCTGGGGAG TACGATCGCA AGATTAAAAC TCAAAGGAAT TGACGGGGGC TCGCACAAGC GGTGGAGCAT
TorTagoi  AATT-TCTTT CGGTTTCGTA GCTAACGCAT TAAGCACTCC GCCTGGGGAG TACGATCGCA AGATTAAAAC TCAAAGGAAT TGACGGGG-G TCGCACAAGC GGTGGAGCAT
HemMarg2  AATT-TCTTT CAGTTTCGTA GCTAACGCAT TAAGCACTCC GCCTGGGGAG TACGGTCGCA AGATTAAAAC TCAAAGGAAT TGACGGGGGC TCGCACAAGC GGTGGAGCAT
HydRick1  T-TT-ATTCT CGGTTTCGCC ACTAACGCAT TAAGCACTCC GCCTGGGGAC TACGATCGCA AGATTAAAAC TCAAAGGAAT TGACGGGGGC TCGCACAAGT GGTGGAGCAT
IchMulti  ATTT-TCTTT CGGTTTCGCA GCTAACGCAT TAAGCACTCC GCCTGGGGAC TACGATCGCA AGATTAAAAC TCAAAGGAAT TGACGGGGGC TCGCACAAGT GGTGGAGCAT
BryRick1  TTAA-ATTCT CGGTTTCGCA GCTAACGCAT TAAGCACTCC GCCTGGGGAC TACGATCGCA AGATTAAAAC TCAAAGGAAT TGACGGGGGC TCGCACAAGT GGTGGAGCAT
BryRick2  TTTA-TTTTT CGGTTTCGCA GCTAACGCAT TAAGCACTCC GCCTGGGGAC TACGATCGCA AGATTAAAAC TCAAAGGAAT TGACGGGGGC TCGCACAAGT GGTGGAGCAT
BryRick3  TTAA-ATTCT CGGTTTCGCA GCTAACGCAT TAAGCACTCC GCCTGGGGAC TACGATCGCA AGATTAAAAC TCAAAGGAAT TGACGGGGGC TCGCACAAGT GGTGGAGCAT
MonFaveo  TTAA-ATTTT CGGTTTCGCA GCTAACGCAT TAAGCACTCC GCCTGGGGAC TACGATCGCA AGATTAAAAC TCAAAGGAAT TGACGGGGGC TCGCACAAGT GGTGGAGCAT
RicNepCi  AATT-TCTTT CGGTTTCGTA GCTAACGCAT TAAGCACTCC GCCTGGGGAG TACGGTCGCA AGATTAAAAC TCAAAGGAAT TGACGGGGGC TCGCACAAGC GGTGGAGCAT
Kalahari  TTTT-TCTTT CGGTTTCGTA GTTAACACGT TAAGCACTCC GCCTGGGGAG TACGATCGCA AGATTAAAAC TCAAAGGAAT TGACGGGGGC TCGCACAAGT GGTGGAGCAT
T6025C27  ATTT-TCTTT CGGTTTCGTA GCTAACGCAT TAAGCACTCC GCCTGGGGAG TACGATCGCA AGATTAAAAC TCAAAGGAAT TGACGGGGGC TCGCACAAGT GGTGGAGCAT
T6025C01  TTTT-TCTTT CGGTTTCGTA GTTAACACGT TAAGCACTCC GCCTGGGGAG TACGATCGCA AGATTAAAAC TCAAAGGAAT TGACGGGGGC TCGCACAAGT GGTGGAGCAT
WakWat77  AATT-TCTTT CGGTTTCGTA GCTAACGCAT TAAGCACTCC GCCTGGGGAG TACGACCGCA AGATTAAAAC TCAAAGGAAT TGACGGGGGC TCGCACAAGT GGTGGAGCAT
Waste214  TTTT-TCTTT CGGTTTCGTA GTTAACACGT TAAGCACTCC GCCTGGGGAG TACGATCGCA AGATTAAAAC TCAAAGGAAT TGACGGGGGC TCGCACAAGT GGTGGAGCAT
TaihAS94  TTTT-TCTTT CGGTTTCGTA GTTAACACGT TAAGCACTCC GCCTGGGGAG TACGATCGCA AGATTAAAAC TCAAAGGAAT TGACGGGGGC TCGCACAAGT GGTGGAGCAT
HydRick2  T-TT-ATTCT CGGTTTCGCC ACTAACGCAT TAAGCACTCC GCCTGGGGAC TACGATCGCA AGATTAAAAC TCAAAGGAAT TGACGGGGGC TCGCACAAGT GGTGGAGCAT
DioOBOD9  TTTT-TCTTT CGGTTTCGTA GTTAACACGT TAAGCACTCC GCCTGGGGAG TACGATCGCA AGATTAAAAC TCAAAGGAAT TGACGGGGGC TCGCACAAGT GGTGGAGCAT
PrmCauda  TTTT-TCTTT CGGTTTCGTA GTTAACACGT TAAGCACTCC GCCTGGGGAG TACGATCGCA AGATTAAAAC TCAAAGGAAT TGACGGGGGC TCGCACAAGT GGTGGAGCAT
Sprsmm01  TTTT-TCTTT CGGTTTCGTA GTTAACACGT TAAGCACTCC GCCTGGGGAG TACGATCGCA AGATTAAAAC TCAAAGGAAT TGACGGGGGC TCGCACAAGT GGTGGAGCAT
DioDS124  TTTT-TCTTT CGGTTTCGTA GTTAACACGT TAAGCACTCC GCCTGGGGAG TACGATCGCA AGATTAAAAC TCAAAGGAAT TGACGGGGGC TCGCACAAGT GGTGGAGCAT
EupOct01  TTTT-TCTTT CGGTTTCGTA GTTAACACGT TAAGCACTCC GCCTGGGGAG TACGATCGCA AGATTAAAAC TCAAAGGAAT TGACGGGGGC TCGCACAAGT GGTGGAGCAT
NIES_425  TTTT-TCTTT CGGTTTCGTA GTTAACACGT TAAGCACTCC GCCTGGGGAG TACGATCGCA AGATTAAAAC TCAAAGGAAT TGACGGGGGC TCGCACAAGT GGTGGAGCAT
NIES_577  TTTT-TCTTT CGGTTTCGTA GTTAACACGT TAAGCACTCC GCCTGGGGAG TACGATCGCA AGATTAAAAC TCAAAGGAAT TGACGGGGGC TCGCACAAGT GGTGGAGCAT
UTEX2180  TTTT-TCTTT CGGTTTCGTA GTTAACACGT TAAGCACTCC GCCTGGGGAG TACGATCGCA AGATTAAAAC TCAAAGGAAT TGACGGGGGC TCGCACAAGT GGTGGAGCAT
EVE_____  ---------- ---------- ---------- ---------- ---------- ---------- ---------- ---------- ---------- ---------- ----------
NIES_732  T-TT-TCTTT CGGTTTCGTA GTTAACACGT TAAGCACTCC GCCTGGGGAG TACAATCGCA AGATTAAAAC TCAAAGGAAT TGACGGGGGC TCGCACAAGT GGTGGAGCAT
OrnTsIke  ATTTTTCTTT CAGTTTCGTA GCTAACGCAT TAAGCACTCC GCCTGGGGAG TACGGTCGCA AGATTAAAAC TCAAAGGAAT TGACGGGGGC CCGCACAAGC GGTGGAGCAT
OrnTsBor  ATTTTTCTTT CAGTTTCGTA GCTAACGCAT TAAGCACTCC GCCTGGGGAG TACGGTCGCA AGATTAAAAC TCAAAGGAAT TGACGGGGGC CCGCACAAGC GGTGGAGCAT
CprPolyt  TTTT-ATTCT CGGTTTCGCA GCTAACGCAT TAAGCACTCC GCCTGGGGAG TACGGTCGCA AGATTAAAAC TTAAAGGAAT TGACGGGGGC CCGCACAAGC GGTGGAGCAT
```

```
           881
RicRicIo   GCGGTTTAAT TCGATGTTAC GCGAAAAACC TTACCAACCC TTGACATGGT GGTTGCGGAT CGC--A-GA- G-ATGCTTTT CTTCAGCTCG GCTGGACCAC ACACAGGTGT
RicLimo2   GCGGTTTAAT TCGATGTTAC GCGAAAAACC TTACCAACCC TTGACATGGT GGTCGCGGGA AGC--A-GA- G-ATGCATCC CTTCAGTTCG GCTGGACCAC ACACAGGTGT
RicFeli2   GCGGTTTAAT TCGATGTTAC GCGAAAAACC TTACCAACCC TTGACATGGT GGTCGCGGAT CGC--A-GA- G-ATGCTTTC CTTCAGCTCG GCTGGACCAC ACACAGGTGT
RicProwa   GCGGTTTAAT TCGATGTTAC GCGAAAAACC TTACCAACCC TTGACATGGT GGTTACGGAT TGC--A-GA- G-ATGCTTTC CTTCAGTTCG GCTGGGCCAC ACACAGGTGT
RicTyphi   GCGGTTTAAT TCGATGTTAC GCGAAAAACC TTACCAACCC TTGACATGGT GGTTATGGAT TGC--A-GA- G-ATGCTTTC CTTCAGTTCG GCTGGGCCAC ACACAGGTGT
RicCono4   GCGGTTTAAT TCGATGTTAC GCGAAAAACC TTACCAACCC TTGACATGGT GGTCGCGGAT CGC--A-GA- G-ATGCTTTT CTTCAGCTCG GCTGGACCAC ACACAGGTGT
RicEmpPa   GCGGTTTAAT TCGATGTTAC GCGAAAAACC TTACCAACCC TTGATATGGT GGTCGCGGAT CGC--A-GA- G-ATGCTTTC CTTCAGTTCG GCTGGACCAC ACACAGGTGT
RicJapon   GCGGTTTAAT TCGATGTTAC GCGAAAAACC TTACCAACCC TTGACATGGT GGTCGCGGAT CGC--A-GA- G-ATGCTTTT CTTCAGCTCG GCTGGATCAC ACACAGGTGT
RicCana2   GCGGTTTAAT TCGATGTTAC GCGAAAAACC TTACCAACCC TTGACATGGT GGTCGCGGAT TGC--A-GA- G-ATGCTTTC CTTCAGTTCG GCTGGACCAC ACACAGGTGT
RicHelve   GCGGTTTAAT TCGATGTTAC GCGAAAAACC TTACCAACCC TTGACATGGT GGTCGCGGAT CGC--A-GA- G-ATGCTTTC CTTCAGCTCG GCTGGACCAC ACACAGGTGT
RicAust3   GCGGTTTAAT TCGATGTTAC GCGAAGAACC TTACCAACCC TTGACATGGT GGTTGCGGAT CGC--A-GA- G-ATGCTTTC CTTCAGCTCG GCTGGACCAC ACACAGGTGT
RicBell3   GCGGTTTAAT TCGATGTTAC GCGAAAAACC TTACCAACCC TTGACATGGT GGTCGCGGAT CGC--A-GA- G-ATGCTTTC CTTCAGTTCG GCTGGACCAC ACACAGGTGT
RicAkar3   GCGGTTTAAT TCGATGTTAC GCGAAAAACC TTACCAACCT TTGACATGGT GGTTGCGGAT CGC--A-GA- G-ATGCTTTC CTTCAGTTCG GCTGGACCAC ACACAGGTGT
TorTsuku   GCGGTTTAAT TCGATGTTAC GCGAAAAACC TTACCAACCC TTGACATGGT GGTCGCGGGA AGCGAAGCAG AGATGCATCC CTTCAGTTCG GCTGGACCAC ACACAGGTGT
TorTagoi   GCGGTTTAAT TCGATGTTAC GCGAAAAACC TTACCAACCC TTGACATGGT GGTCGCGGGA AGCGAAGCAG AGATGCATCC CTTCAGTTCG GCTGGACCAC ACACAGGTGT
HemMarg2   GCGGTTTAAT TCGATGTTAC GCGAAAAACC TTACCAACCC TTGACATGGT GGTCGCGGGA AGC--A-GA- G-ATGCATCC CTTCAGTTC- GCTGGACCAC ACACAGGTGT
HydRick1   GCGGTTTAAT TCGAATCTAC GCGAAAAACC TTACCAACCC TTGACATGGT GGTCGCGGGG ACC--A-GA- G-ATGGACCC CTTCAGTTCG GCTGGACCAC ACACAGGTGT
IchMulti   GCGGTTTAAT TCGATGCTAC GCGAAAAACC TTACCAACCC TTGACATGGT GGTCATAGAT TCC--A-GA- G-ATGGATTC G-TCAGTTCG GCTGGACCAC ACACAGGTGT
BryRick1   GCGGTTTAAT TCGATGCTAC GCGAAAAACC TTACCAACCC TTGACATGGT GGTCGCGGGT CTC--A-GA- G-ATGAGACC CTTCAGTTCG GCTGGACCAC ACACAGGTGT
BryRick2   GCGGTTTAAT TCGATGCTAC GCGAAAAACC TTACCAACCC TTGACATGGT GGTCGCGGGG ACC--A-GA- G-ATGGACCC TTTCAGTTCG GCTGGACCAC ACACAGGTGT
BryRick3   GCGGTTTAAT TCGATGCTAC GCGAAAAACC TTACCAACCC TTGACATGGT GGTCGCGGGT CTC--A-GA- G-ATGAGACC CTTCAGTTCG GCTGGACCAC ACACAGGTGT
MonFaveo   GCGGTTTAAT TCGATGCTAC GCGAAAAACC TTACCAACCC TTGACATGGT GGTCGCGAGA GAC--A-GA- G-ATGACTCT CTTCAGTTCG GCTGGACCAC ACACAGGTGT
RicNepCi   GCGGTTTAAT TCGATGTTAC GCGAAAAACC TTACCAACCC TTGACATGGT GGTCGCGGGA AGC--A-GA- G-ATGTATCC CTTCAGTTCG GCTGGACCAC ACACAGGTGT
Kalahari   GCGGCTTAAT TCGATGCTAC GCGAAAAACC TTACCAACCC TTGACATGGT GATCGCAGGT TAC--A-GA- A-ATGTAACC G-TCAGTTCG GCTGGATCAC ACACAGGTGT
T6025C27   GCGGTTTAAT TCGATGCTAC GCGAAAAACC TTACCAACCC TTGACATGGT GATCGTAAGC TGT--A-GA- G-ATATAGCT G-TCAGTTAG GCTGGATCAC ACACAGGTGT
T6025C01   GCGGTTTAAT TCGATGCTAC GCGAAAAACC TTACCAACCC TTGACATGGT GATCGTAGGT TAC--A-GA- A-ATGTAACC G-TCAGTTCG GCTGGATCAC ACACAGGTGT
WakWat77   GCGGTTTAAT TCGATGCTAC GCGAAAAACC TTACCAACCC TTGACATGGT GATCGTAGGC TAC--A-GA- G-ATGTAGCC G-TCAGTTCG GCTGGATCAC ACACAGGTGT
Waste214   GCGGTTTAAT TCGATGCTGC GCGAAAAACC TTGCCAACCC TTGACATGGT GATCGTAGGT TAC--A-GA- A-ATGTAACC G-TCAGTTCG GCTGGATCAC ACACAGGTGT
TaihAS94   GCGGTTTAAT TCGATGCTAC GCGAAAAACC TTACCAACCC TTGACATGGT GATCGTAGGT TAC--A-GA- A-ATGTAAGC G-TCAGTTCG GCTGGATCAC ACACAGGTGT
HydRick2   GCGGTTTAAT TCGGATCTAC GCGAAAAACC TTACCAACCC TTGACATGGT GGTCGCGGGG ACC--A-GA- G-ATGGACCC CTTCAGTTCG GCTGGACCAC ACACAGGTGT
DioOBOD9   GCGGTTTAAT TCGATGCTAC GCGAAAAACC TTACCAACCC TTGACATGGT GATCGTAGGT TAC--A-GA- A-ATGTAACC G-TCAGTTCG GCTGGATCAC ACACAGGTGT
PrmCauda   GCGGTTTAAT TCGATGCTAC GCGAAAAACC TTACCAACCC TTGACATGGT GATCGTAGGT TAC--A-GA- A-ATGTAACC G-TCAGTTCG GCTGGATCAC ACACAGGTGT
Sprsmm01   GCGGTTTAAT TCGATGCTAC GCGAAAAACC TTACCAACCC TTGACATGGT GATCGTAGGT TAC--A-GA- A-ATGTAACC G-TCAGTTCG GCTGGATCAC ACACAGGTGT
DioDS124   GCGGTTTAAT TCGATGCTAC GCGAAAAACC TTACCAACCC TTGACATGGT GATCGTAGGT TAC--A-GA- A-ATGTAACA G-TCAGTTCG GCTGGATCAC ATACAGGTGT
EupOct01   GCGGTTTAAT TCGATGATAC GC-AAAAACC TTACCAACCC TTGACATGGT GATCGTAGGT TAC--A-GA- A-ATGTAACC G-TCAGTTCG GCTGGATCAC ACACAGGTGT
NIES_425   GCGGTTTAAT TCGATGCTAC GCGAAAAACC TTACCAACCC TTGACATGGT GATCGTAGGT TAC--A-GA- A-ATGTAACC G-TCAGTTCG GCTGGATCAC ACACAGGTGT
NIES_577   GCGGTTTAAT TCGATGCTAC GCGAAAAACC TTACCAACCC TTGACATGGT GATCGTAGGT TAC--A-GA- A-ATGTAACC G-TCAGTTCG GCTGGATCAC ACACAGGTGT
UTEX2180   GCGGTTTAAT TCGATGCTAC GCGAAAAACC TTACCAACCC TTGACATGGT GATCGTAGGT TAC--A-GA- A-ATGTAACC G-TCAGTTCG GCTGGATCAC ACACAGGTGT
EVE_____   ---------- ---------- ---------- ---------- ---------- ---------- ---------- ---------- ---------- ---------- ----------
NIES_732   GCGGTTTAAT TCGATGCTAC GCGAAAAACC TTACCAACCC TTGTCATGGT GATCGTAGGC TAC--A-AA- A-ATGTAACC G-TCAGTTCG GCTGGATCAC ACACAGGTGT
OrnTsIke   GCGGTTTAAT TCGATGATCC GCGAAAAACC TTACCAACTC TTGACATGGT AGTCGCGAAA AAT--G-GA- G-ACATTTTT CTTCAGTTTG GCTGGACTAC ACACAGGTGT
OrnTsBor   GCGGTTTAAT TCGATGATCC GCGAAAAACC TTACCAACTC TTGACATGGT AGTCGCGAAA AAT--G-GA- G-ACATTTTT CTTCAGTTTG GCTGGACTAC ACACAGGTGT
CprPolyt   GCGGTTTAAT TCGATGCTAC GCGAAAAACC TTACCAACTC TTGACATGGT GGTTAGGAAG AAC--A-GA- G-ATGTTTTT CTTCGGTTCG GCCGGGCCAC ACACAGGTGC
```

```
          991
RicRicIo   TGCATGGCTG TCGTCAGCTC GTGTCGTGAG ATGTTGGGTT AAGTCCCGCA ACGAGCGCAA CCCTCATTCT TATTTGCCAG CGGGTAATGC CGGGAACTAT AAGAAAACTG
RicLimo2   TGCATGGCTG TCGTCAGCTC GTGTCGTGAG ATGTTGGGTT AAGTCCCGCA ACGAGCGCAA CCCTCATTCT TATTTGCCAG CGGGTAATGC CGGGAACTAT AAGGAAACTG
RicFeli2   TGCATGGCTG TCGTCAGCTC GTGTCGTGAG ATGTTGGGTT AAGTCCCGCA ACGAGCGCAA CCCTCATTCT TATTTGCCAG CGGGTAATGC CGGGAACTAT AAGAAAACTG
RicProwa   TGCATGGCTG TCGTCAGCTC GTGTCGTGAG ATGTTGGGTT AAGTCCCGCA ACGAGCGCAA CCCTTATTCT TATTTGCCAG TGGGTAATGC CGGGAACTAT AAGAAAACTG
RicTyphi   TGCATGGCTG TCGTCAGCTC GTGTCGTGAG ATGTTGGGTT AAGTCCCGCA ACGAGCGCAA CCCTTATTCT TATTTGCCAG CGGGTAATGC CGGGAACTAT AAGAAAACTG
RicCono4   TGCATGGCTG TCGTCAGCTC GTGTCGTGAG ATGTTGGGTT AAGTCCCGCA ACGAGCGCAA CCCTCATTCT TATTTGCCAG CGGGTAATGC CGGGAACTAT AAGAAAACTG
RicEmpPa   TGCATGGCTG TCGTCAGCTC GTGTCGTGAG ATGTTGGGTT AAGTCCCGCA ACGAGCGCAA CCCTCATTCT TATTTGCCAG CGGGTAATGC CGGGAACTAT AAGAAAACTG
RicJapon   TGCATGGCTG TCGTCAGCTC GTGTCGTGAG ATGTTGGGTT AAGTCCCGCA ACGAGCGCAA CCCTCATTCT TATTTGCCAG CGGGTAATGC CGGGAACTAT AAGAAAACTG
RicCana2   TGCATGGCTG TCGTCAGCTC GTGTCGTGAG ATGTTGGGTT AAGTCCCGCA ACGAGCGCAA CCCTCATTCT TATTTGCCAG CGGATAATGC CGGGAACTAT AAGAAAACTG
RicHelve   TGCATGGCTG TCGTCAGCTC GTGTCGTGAG ATGTTGGGTT AAGTCCCGCA ACGAGCGCAA CCCTCATTCT TATTTGCCAG CGGGTAATGC CAGGAACTAT AAGAAAACTG
RicAust3   TGCATGGCTG TCGTCAGCTC GTGTCGTGAG ATGTTGGGTT AAGTCCCGCA ACGAGCGCAA CCCTCATTCT TATTTGCCAG CGGGTAATGC CGGGAACTAT AAGAAAACTG
RicBell3   TGCATGGCTG TCGTCAGCTC GTGTCGTGAG ATGTTGGGTT AAGTCCCGCA ACGAGCGCAA CCCTCATTCT TATTTGCCAG CGGGTAATGC CGGGAACTAT AAGAAAACTG
RicAkar3   TGCATGGCTG TCGTCAGCTC GTGTCGTGAG ATGTTGGGTT AAGTCCCGCA ACGAGCGCAA CCCTCATTCT TATTTGCCAG CGGGTAATGC CGGGAACTAT AAGAAAACTG
TorTsuku   TGCATGGCTG TCGTCAGCTC GTGTCGTGAG ATGTTGGGTT AAGTCCCGCA ACGAGCGCAA CCCTCATTCT TATTTGCCAG CGGGTAATGC CGGGAACTAT AAGGAAACTG
TorTagoi   TGCATGGCTG TCGTCAGCTC GTGTCGTGAG ATGTTGGGTT AAGTCCCGCA ACGAGCGCAA CCCTCATTTT TATTTGCCAG CGGGTAATGC CGGGAACTAT AAGGAAACTG
HemMarg2   TGCATGGCTG TCGTCAGCTC GTGTCGTGAG ATGTTGGGTT AAGTCCCGCA ACGAGCGCAA CCCTCATTAT TATTTGCCAG CGGGTAATGC CGGGAACTAT AAGGAAACTG
HydRick1   TGCATGGCTG TCGTCAGCTC GTGTCGTGAG ATGTTGGGTT AAGTCCCGCA ACGAGCGAAA CCCTCATCCT TATTTGCCAG CGGTTCG-GC CGGGAACTAT AAGGAAACTG
IchMulti   TGCATGGCTG TCGTCAGCTC GTGTCGTGAG ATGTTGGGTT AAGTCCCGCA ACGAGCGAAA CCCTCATCCT TATTTGCCAG CGATTCGG-T CGGGAACTAT AAGGAAACTG
BryRick1   TGCATGGCTG TCGTCAGCTC GTGTCGTGAG ATGTTGGGTT AAGTCCCGCA ACGAGCGAAA CCCTCATCCT TATTTGCCAG CGGTTCG-GC CGGGGACTAT AAGGAAACTG
BryRick2   TGCATGGCTG TCGTCAGCTC GTGTCGTGAG ATGTTGGGTT AAGTCCCGCA ACGAGCGAAA CCCTCATCCT TATTTGCCAG CGGTTCG-GC CGGGAACTAT AAGGAAACTG
BryRick3   TGCATGGCTG TCGTCAGCTC GTGTCGTGAG ATGTTGGGTT AAGTCCCGCA ACGAGCGAAA CCCTCATCCT TATTTGCCAG CGGTTCG-GC CGGGGACTAT AAGGAAACTG
MonFaveo   TGCATGGCTG TCGTCAGCTC GTGTCGTGAG ATGTTGGGTT AAGTCCCGCA ACGAGCGAAA CCCTCATCCT TATTTGCCAG CGGTTCG-GC CGGGGACTAT AAGGAAACTG
RicNepCi   TGCATGGCTG TCGTCAGCTC GTGTCGTGAG ATGTTGGGTT AAGTCCCGCA ACGAGCGCAA CCCTCATTCT TATTTGCCAG CGGGTAATGC CGGGAACTAT AAGGAAACTG
Kalahari   TGCATGGCTG TCGTCAGCTC GTGTCGTGAG ATGTTGGGTT AAGTCCCGCA ACGAGCGCAA CCCTCATCCT TATTTGCCAG CGGTTAG-GC CGGGAACTAT AAGGAAACTG
T6025C27   TGCATGGCTG TCGTCAGCTC GTGTCGTGAG ATGTTGGGTT AAGTCCCGCA ACGAGCGCAA CCCTCATTCT TATTTGCCAG CGGTTCG-GC CGGGAACTAT AAGAAAACTG
T6025C01   TGCATGGCTG TCGTCAGCTC GTGTCGTGAG ATGTTGGGTT AAGTCCCGCA ACGAGCGCAA CCCTCATCCT TATTTGCCAG CGGTTAG-GC CGGGAACTAT AAGGAAACTG
WakWat77   TGCATGGCTG TCGTCAGCTC GTGTCGTGAG ATGTTGGGTT AAGTCCCGCA ACGAGCGCAA CCCTCATCCT TATTTGCCAG CGGTTCG-GC CGGGGACTAT AAGGAAACTG
Waste214   TGCATGGCTG TCGTCAGCTC GTGTCGTGAG ATGTTGGGTT AAGTCCCGCA ACGAGCGCAA CCCTCATCCT TATTTGCCAG CGGTTAG-GC CGGGAACTAT AAGGAAACTG
TaihAS94   TGCATGGCTG TCGTCAGCTC GTGTCGTGAG ATGTTGGGTT AAGTCCCGCA ACGAGCGCAA CCCTCATCCT TATTTGCCAG CGGTTAG-GC CGGGAACTAT AAGGAAACTG
HydRick2   TGCATGGCTG TCGTCAGCTC GTGTCGTGAG ATGTTGGGTT AAGTCCCGCA ACGAGCGAAA CCCTCATCCT TATTTGCCAG CGGTTCG-GC CGGGAACTAT AAGGAAACTG
DioOBOD9   TGCATGGCTG TCGTCAGCTC GTGTCGTGAG ATGTTGGGTT AAGTCCCGCA ACGAGCGCAA CCCTCATCCT TATTTGCCAG CGGTTAG-GC CGGGAACTAT AAGGAAACTG
PrmCauda   TGCATGGCTG TCGTCAGCTC GTGTCGTGAG ATGTTGGGTT AAGTCCCGCA ACGAGCGCAA CCCTCATCCT TATTTGCCAG CGGTTAG-GC CGGGAACTAT AAGGAAACTG
Sprsmm01   TGCATGGCTG TCGTCAGCTC GTGTCGTGAG ATGTTGGGTT AAGTCCCGCA ACGAGCGCAA CCCTCATCCT TATTTGCCAG CGGTTAG-GC CGGGAACTAT AAGGAAACTG
DioDS124   TGCATGGCTG TCGTCAGCTC GTGTCGTGAG ATGTTGGGTT AAGTCCCGCA ACGAGCGCAA CCCTCATCCT TATTTGCCAG CGGTTAG-GC CGGGAACTAT AAGGAAACTG
EupOct01   TGCATGGCTG TCGTCAGCTC GTGTCGTGAG ATGTTGGGTT AAGTCCCGCA ACGAGCGCAA CCCTCATCCT TATTTGCCAG CGGTTAG-GC CGGGAACTAT AAGGAAACTG
NIES_425   TGCATGGCTG TCGTCAGCTC GTGTCGTGAG ATGTTGGGTT AAGTCCCGCA ACGAGCGCAA CCCTCATCCT TATTTGCCAG CGGTTAG-GC CGGGAACTAT AAGGAAACTG
NIES_577   TGCATGGCTG TCGTCAGCTC GTGTCGTGAG ATGTTGGGTT AAGTCCCGCA ACGAGCGCAA CCCTCATCCT TATTTGCCAG CGGTTAG-GC CGGGAACTAT AAGGAAACTG
UTEX2180   TGCATGGCTG TCGTCAGCTC GTGTCGTGAG ATGTTGGGTT AAGTCCCGCA ACGAGCGCAA CCCTCATCCT TATTTGCCAG CGGTTAG-GC CGGGAACTAT AAGGAAACTG
EVE_____   ---------- ---------- ---------- ---------- ---------- ---------- ---------- ---------- ---------- ---------- ----------
NIES_732   TGCATGGCTG TCGTCAGCTC GTGTCGTGAG ATGTTGGTTT AAGTCCCGCA ACGAGTGCAA CCCTCATCCT TGTTTGCCAG CGGTTAG-GC CGGGAACTAT AAGGAAACTG
OrnTsIke   TGCATGGCTG TCGTCAGCTC GTGTCGTGAG ATGTTGGGTT AAGTCCCGCA ACGAGCGCAA CCCTTATTCT TATTTGCCAG CGGGTAATGC CGGGAACTAT AGGAAAACTG
OrnTsBor   TGCATGGCTG TCGTCAGCTC GTGTCGTGAG ATGTTGGGTT AAGTCCCGCA ACGAGCGCAA CCCTTATTCT TATTTGCCAG CGGGTAATGC CGGGAACTAT AGGAAAACTG
CprPolyt   TGCATGGCTG TCGTCAGCTC GTGTCGTGAG ATGTTGGGTT AAGTCCCGTA ACGAGCGCAA CCCTTATCCT TATTTGCCAT CAGGTTAAGC TGGGAACTAT AAGGAAACTG
```

```
      1101
RicRicIo   CCGGTGATAA GCCGGAGGAA GGTGGGGACG ACGTCAAGTC ATCATGGCCC TTAC-GGGTT GGGCTACACG CGTGCTACAA TGGTGTTTAC AGAGGGAAGC AAGACGGCGA
RicLimo2   CCGGTGATAA GCCGGAGGAA GGTGGGGACG ATGTCAAGTC ATCATGGCCC TTAT-GGGTT GGGCTACACG CGTGCTACAA TGGTATCCAC AGAGGGAAGC AAGACGGTGA
RicFeli2   CCGGTGATAA GCCGGAGGAA GGTGGGGACG ACGTCAAGTC ATCATGGCCC TTAC-GGGTT GGGCTACACG CGTGCTACAA TGGTGTTTAC AGAGGGAAGC AAGACGGCGA
RicProwa   CCGGTGATAA GCCGGAGGAA GGTGGGGACG ACGTCAAGTC ATCATGGCCC TTAC-GGGTT GGGCTACACG CGTGCTACAA TGGTGTTTAC AGAGGGAAGC AATACGGTGA
RicTyphi   CCGGTGATAA GCCGGAGGAA GGTGGGGACG ACGTCAAGTC ATCATGGCCC TTAC-GGGTT GGGCTACACG CGTGCTACAA TGGTGTTTAC AGAGGGAAGC AAGACGGTGA
RicCono4   CCGGTGATAA GCCGGAGGAA GGTGGGGACG ACGTCAAGTC ATCATGGCCC TTAC-GGGTT GGGCTACACG CGTGCTACAA TGGTGTTTAC AGAGGGAAGC AAGACGGCGA
RicEmpPa   CCGGTGATAA GCCGGAGGAA GGTGGGGACG ACGTCAAGTC ATCATGGCCC TTAC-GGGTT GGGCTACACG CGTGCTACAA TGGTGTTTAC AGAGGGAAGC AAGACGGCGA
RicJapon   CCGGTGATAA GCCGGAGGAA GGTGGGGACG ACGTCAAGTC ATCATGGCCC TTAC-GGGTT GGGCTACACG CGTGCTACAA TGGTGTTTAC AGAGGGAAGC AAGACGGCGA
RicCana2   CCAGTGATAA GCTGGAGGAA GGTGGGGACG ACGTCAAGTC ATCATGGCCC TTAC-GGGTT GGGCTACACG CGTGCTACAA TGGTGTTTAC AGAGGGAAGC AAAACGGCGA
RicHelve   CCGGTGATAA GCCGGAGGAA GGTGGGGACG ACGTCAAGTC ATCATGGCCC TTAC-GGGTT GGGCTACACG CGTGCTACAA TGGTGTTTAC AGAGGGAAGC AAGACGGCGA
RicAust3   CCGGTGATAA GCCGGAGGAA GGTGGGGACG ACGTCAAGTC ATCATGGCCC TTAC-GGGTT GGGCTACACG CGTGCTACAA TGGTGTTTAC AGAGGGAAGC AAGACGGCGA
RicBell3   CCGGTGATAA GCCGGAGGAA GGTGGGGACG ACGTCAAGTC ATCATGGCCC TTAC-GGGTT GGGCTACACG CGTGCTACAA TGGTGTTTAC AGAGGGAAGC AAGACGGCGA
RicAkar3   CCGGTGATAA GCCGGAGGAA GGTGGGGACG ACGTCAAGTC ATCATGGCCC TTAT-GGGTT GGGCTACACG CGTGCTACAA TGGTGTTTAC AGAGGGAAGC AAGACGGTAA
TorTsuku   CCGGTGATAA GCCGGAGGAA GGTGGGGACG ATGTCAAGTC ATCATGGCCC TTAT-GGGTT GGGCTACACG CGTGCTACAA TGGTATCCAC AGAGGGAAGC AAGACGGTGA
TorTagoi   CCGGTGATAA GCCGGAGGAA GGTGGGGACG ATGTCAAGTC ATCATGGCCC TTAT-GGGTT GGGCTACACG CGTGCTACAA TGGTATCCAC AGAGGGAAGC AAGACGGTGA
HemMarg2   CCGGTGATAA GCTGGAGGAA GGTGGGGACG ATGTCAAGTC ATCATGGCCC TTAT-GGGTT GGGCTACACG CGTGCTACAA TGGTATCCAC AGAGGGAAGC AATACGGTGA
HydRick1   CCGGTGATAA ACCGGAGGAA GGTGGGGACG ATGTCAAGTC ATCATGG-CC TTAC-GGGTT GGGCTACACG CGTGCTACAA TGGTGCTTAC AGAGGGATGC AATACGGCGA
IchMulti   CCGGTGATAA ACCGGAGGAA GGTGGGGACG ATGTCAAGTC ATCATGGCCC TTAC-GGGTT GGGCTACACG CGTGCTACAA TGGTGCTTAC AGAGGGAAGC AATACAGCGA
BryRick1   CCGGTGATAA GCCGGAGGAA GGTGGGGATG ATGTCAAGTC ATCATGGCCC TTAT-GGGTT GGGCTACACG CGTGCTACAA TGGTATCTAC AGAGGGAAGC AATATGGCGA
BryRick2   CCGGTGATAA GCCGGAGGAA GGTGGGGACG ATGTCAAGTC ATCATGGCCC TTAT-GGGTT GGGCTACACG CGTGCTACAA TGGTACCTAC AGAGGGAAGC AATACGGCAA
BryRick3   CCGGTGATAA GCCGGAGGAA GGTGGGGATG ATGTCAAGTC ATCATGGCCC TTAT-GGGTT GGGCTACACG CGTGCTACAA TGGTATCTAC AGAGGGAAGC AATATGGCGA
MonFaveo   CCGGTGATAA GCCGGAGGAA GGTGGGGACG ATGTCAAGTC ATCATGGCCC TTAC-GGGTT GGGCTACACG CGTGCTACAA TGGTATCTAC AGAGGGAAGC AATATGGCAA
RicNepCi   CCGGTGATAA GCCGGAGGAA GGTGGGGACG ATGTCAAGTC ATCATGGCCC TTAC-GGGTT GGGCTACACG CGTGCTACAA TGGTATCTAC AGAGGGAAGC AAGACGGTGA
Kalahari   CCGGTGATAA GCCGGAGGAA GGTGGGGATG ATGTCAAGTC ATCATGGCCC TTAC-GGGTT GGGCTACACG CGTGCTACAA TGGTACTTAC AGAGGGAAGC AAGACGGCGA
T6025C27   CCGGTGATAA ACCGGAGGAA GGTGGGGACG ATGTCAAGTC ATCATGGCCC TTAC-GGGTT GGGCTACACG CGTGCTACAA TGGTACTTAC AGAGGGAAGC AAGACGGCGA
T6025C01   CCGGTGATAA GCCGGAGGAA GGTGGGGATG ATGTCAAGTC ATCATGGCCC TTAC-GGGTT GGGCTACACG CGTGCTACAA TGGTACTTAC AGAGGGAAGC AAGACGGCGA
WakWat77   CCGGTGATAA ACCGGAGGAA GGTGGGGATG ATGTCAAGTC ATCATGGCCC TTAC-GGGTT GGGCTACACG CGTGCTACAA TGGTACCTAC AGAGGGAAGC AATACGGCGA
Waste214   CCGGTGATAA GCCGGAGGAA GGTGGGGATG ATGTCAAGTC ATCATGGCCC TTAC-GGGTT GGGCTACACG CGTGCTACAA TGGTACTTAC AGAGGGAAGC AAGACGGCGA
TaihAS94   CCGGTGATAA GCCGGAGGAA GGTGGGGATG ATGTCAAGTC ATCATGGCCC TTAC-GGGTT GGGCTACACG CGTGCTACAA TGGTACTTAC AGAGGGAAGC AAGACGGCGA
HydRick2   CCGGTGATAA ACCGGAGGAA GGTGGGGACG ATGTCAAGTC ATCATGG-CC TTAC-GGGTT GGGCTACACG CGTGCTACAA TGGTGCTTAC AGAGGGATGC AATACGGCGA
DioOBOD9   CCGGTGATAA GCCGGAGGAA GGTGGGGATG ATGTCAAGTC ATCATGGCCC TTAC-GGGTT GGGCTACACG CGTGCTACAA TGGTACTTAC AGAGGGAAGC AAGACGGCGA
PrmCauda   CCGGTGATAA GCCGGAGGAA GGTGGGGATG ATGTCAAGTC ATCATGGCCC TTAC-GGGTT GGGCTACACG CGTGCTACAA TGGTACTTAC AGAGGGAAGC AAGACGGCGA
Sprsmm01   CCGGTGATAA GCCGGAGGAA GGTGGGGATG ATGTCAAGTC ATCATGGCCC TTAC-GGGTT GGGCTACACG CGTGCTACAA TGGTACTTAC AGAGGGAAGC AAGACGGCGA
DioDS124   CCGGTGATAA GCCGGAGGAA GGTGGGGATG ATGTCAAGTC ATCATGGCCC TTAC-GGGTT GGGCTACACG CGTGTTACAA TGGTACTTAC AGAGGGAAGC AAGACGGCGA
EupOct01   CCGGTGATAA GCCGGAGGAA GGTGGGGATG ATGTCAAGTC ATCATGGCCC TTAC-GGGTT GGGCTACACG CGTGCTACAA TGGTACTTAC AGAGGGAAGC AAGACGGCGA
NIES_425   CCTGTGATAA GCCGGAGGAA GGTGGGGATG ATGTCAAGTC ATCATGGCCC TTAC-GGGTT GGGCTACACG CGTGCTACAA TGGTACTTAC AGAGGGAAGC AAGACGGCGA
NIES_577   CCGGTGATAA GCCGGAGGAA GGTGGGGATG ATGTCAAGTC ATCATGGCCC TTAC-GGGTT GGGCTACACG CGTGCTACAA TGGTACTTAC AGAGGGAAGC AAGACGGCGA
UTEX2180   CCGGTGATAA GCCGGAGGAA GGTGGGGATG ATGTCAAGTC ATCATGGCCC TTAC-GGGTT GGGCTACACG CGTGCTACAA TGGTACTTAC AGAGGGAAGC AAGACGGCGA
EVE_____   ---------- ---------- ---------- ---------- ---------- ---------- ---------- ---------- ---------- ---------- ----------
NIES_732   CCGGTGATAA ACCGGAGGAA GGTGGGGATG ATGTCAAGTC ATCATGGCCC TTAC-GGGTT GGGCTACACG CGTGCTACAA TGGTACTTAC AGAGGGAAGC AAGACGGCGA
OrnTsIke   CCGGTGCTAA GCCGGAGGAA GGTGGGGATG AGGTCAAGTC ATCATGGCCC TTAT-GAGTT GGGCTACACG CGTGCTACAA TGGTACCTAC AGAATGATGC AATACGGTAA
OrnTsBor   CCGGTGCTAA GCCGGAGGAA GGTGGGGATG AGGTCAAGTC ATCATGGCCC TTAT-GAGTT GGGCTACACG CGTGCTACAA TGGTACCTAC AGAGTGATGC GATACGGTGA
CprPolyt   CCGGCTTTAA GCCGGAGGAA GGTGGGGACG AGGTCAAGTC ATCATGGCCT TTACTGAGTT GGGCTACACG CGTGCTACAA TGGTATCTAC AGAGGGAAGC CAAGTGGCGA
```

```
          1211
RicRicIo   CGTGGAGCAA ATCC-CT-AA AAGACATCTC AGTTCGGATT GTTCTCTGCA ACTCGAGAGC ATGAAGTT-G GAATCGCTAG TAATCGCGGA TCAGCATGCC GCGGTGAATA
RicLimo2   CGTGGAGCAA ATCC-CT-AA AAGATATCTC AGTTCGGATT GTTCTCTGCA ACTCGAGAAC ATGAAGTT-G GAATCGCTAG TAATCGCGGA TCAGCATGCC GCGGTGAATA
RicFeli2   CGTGGAGCAA ATCC-CT-AA AAGACATCTC AGTTCGGATT GTTCTCTGCA ACTCGAGAGC ATGAAGTT-G GAATCGCTAG TAATCGCGGA TCAGCATGCC GCGGTGAATA
RicProwa   CGTGGAGCAA ATCC-CT-AA AAGACATCTC AGTTCGGATT GTTCTCTGCA ACTCGAGAGC ATGAAGTT-G GAATCGCTAG TAATCGCGGA TCAGCATGCC GCGGTGAATA
RicTyphi   CGTGGAGCAA ATCC-CT-AA AAGACATCTC AGTTCGGATT GTTCTCTGCA ACTCGAGAGC ATGAAGTT-G GAATCGCTAG TAATCGCGGA TCAGCATGCC GCGGTGAATA
RicCono4   CGTGGAGCAA ATCC-CT-AA AAGACATCTC AGTTCGGATT GTTCTCTGCA ACTCGAGAGC ATGAAGTT-G GAATCGCTAG TAATCGCGGA TCAGCATGCC GCGGTGAATA
RicEmpPa   CGTGGAGCAA ATCC-CT-AA AAGACATCTC AGTTCGGATT GTTCTCTGCA ACTCGAGAGC ATGAAGTT-G GAATCGCTAG TAATCGCGGA TCAGCATGCC GCGGTGAATA
RicJapon   CGTGGAGCAA ATCC-CT-AA AAGACATCTC AGTTCGGATT GTTCTCTGCA ACTCGAGAGC ATGAAGTT-G GAATCGCTAG TAATCGCGGA TCAGCATGCC GCGGTGAATA
RicCana2   CGTGGAGCAA ATCC-CT-AA AAGACATCTC AGTTCGGATT GTTCTCTGCA ACTCGAGAGC ATGAAGTT-G GAATCGCTAG TAATCGCGGA TCAGCATGCC GCGGTGAATA
RicHelve   CGTGGAGCAA ATCC-CT-AA AAGACATCTC AGTTCGGATT GTTCTCTGCA ACTCGAGAGC ATGAAGTT-G GAATCGCTAG TAATCGCGGA TCAGCATGCC GCGGTGAATA
RicAust3   CGTGGAGCAA ATCC-CT-AA AAGACATCTC AGTTCGGATT GTTCTCTGCA ACTCGAGAGC ATGAAGTT-G GAATCGCTAG TAATCGCGGA TCAGCATGCC GCGGTGAATA
RicBell3   CGTGGAGCAA ATCC-CT-AA AAGACATCTC AGTTCGGATT GTTCTCTGCA ACTCGAGAGC ATGAAGTT-G GAATCGCTAG TAATCGCGGA TCAGCATGCC GCGGTGAATA
RicAkar3   CGTGGAGCAA ATCC-CT-AA AAGACATCTC AGTTCGGATT GTTCTCTGCA ACTCGAGAGC ATGAAGTT-G GAATCGCTAG TAATCGCGGA TCAGCATGCC GCGGTGAATA
TorTsuku   CGTGGAGCAA ATCC-CT-AA AAGATATATC AGTTCGGATT GCTCTCTGCA ACTCGAGAGC ATGAAGTTGG GAATCGCTAG TAATCGCGGA TCAGCATGCC GCGGTGAATA
TorTagoi   CGTGGAGCAA ATCC-CT-AA AAGATATCTC AGTTCGGATT GCTCTCTGCA ACTCGAGAGC ATGAAGTT-G GAATCGCTAG TAATCGCGGA TCAGCATGCC GCGGTGAATA
HemMarg2   CGTGGAGCAA ATCC-CT-AA AAGATATCTC AGTTCGGATT GTTCTCTGCA ACTCGAGAAC ATGAAGTT-G GAATCGCTAG TAATCGCGGA TCAGCATGCC GCGGTGAATA
HydRick1   CGTGGAGCAA ATCC-TC-AA AAGGCATCTC AGTTCGGATT GTTCTCTGCA ACTCGAGAGC ATGAAGTC-G GAATCACTAG TAATCGCGGA TCAGCATGCC GCGGTGAATA
IchMulti   TGTGGAGCAA ATCCTTT-AA AAGGCATCTC AGTTCGGATT GCTCTCTGCA ACTCGAGAGC ATGAAGTC-G GAATCACTAG TAATCGCGGA TCAGCATGCC GCGGTGAATA
BryRick1   CATGGAGCAA ATCC-TC-AA AGGATATCTC AGTTCGGATT GGTCTCTGCA ACTCGAGACC ATGAAGTC-G GAATCACTAG TAATCGCGGA TCAGCATGCC GCGGTGAATA
BryRick2   CGTGGAGCGA ATCC-TC-AA AAGGTATCTC AGTTCGGATT GGTCTCTGCA ACTCGAGACC ATGAAGTC-G GAATCACTAG TAATCGCGGA TCAGCATGCC GCGGTGAATA
BryRick3   CATGGAGCAA ATCC-TC-AA AGGATATCTC AGTTCGGATT GGTCTCTGCA ACTCGAGACC ATGAAGTC-G GAATCACTAG TAATCGCGGA TCAGCATGCC GCGGTGAATA
MonFaveo   CATGGAGCAA ATCC-TC-AA AGGATATCTC GGTTCGGATT GGCCTCTGCA ACTCGAGACC ATGAAGTC-G GAATCACTAG TAATCGCGGA TCAGCATGCC GCGGTGAATA
RicNepCi   CGTGGAGCAA ATCC-CT-AA AAGATATCTC AGTTCGGATT GCTCTCTGCA ACTCGAGAGC ATGAAGTT-G GAATCGCTAG TAATCGCGGA TCAGCATGCC GCGGTGAATA
Kalahari   CGTGGAGCAA ATCC-CT-AA AAAGTATCTC AGTTCGGATT GTTCTCTGCA ACTCGAGAGC ATGAAGTC-G GAATCACTAG TAATCGCGGA TCAGCATGCC GCGGTGAATA
T6025C27   CGTGGAGCAA ATCC-CT-AA AAAGTATCTC AGTTCAGATT GTTCTCTGCA ACTCGAGAGC ATGAAGTC-G GAATCACTAG TAATCGCGGA TCAGCATGCC GCGGTGAATA
T6025C01   CGTGGAGCAA ATCC-CT-AA AAAGTATCTC AATTCGGATT GCTCTCTGCA ACTCGAGAGC ATGAAGTC-G GAATCACTAG TAATCGCGGA TCAGCATGCC GCGGTGAATA
WakWat77   CGTGGAGCAA ATCC-CT-AA AAAGTATCTC AGTTCGGATT GCTCTCTGCA ACTCGAGAGC ATGAAGTC-G GAATCACTAG TAATCGCGGA TCAACATGCC GCGGTGAATA
Waste214   CGTGGAGCAA ATCC-CT-AA AAAGTATCTC AGTTCGGATT GCTCTCTGCA ACTCGAGAGC ATGAAGTC-G GAATCACTAG TAATCGCGGA TCAGCATGCC GCGGTGAATA
TaihAS94   CGTGAAGCAA ATCC-CT-AA AAAGTATCTC AGTTCGGATT GTTCTCTGCA ACTCGAGAGC ATGAAGTC-G GAATCACTAG TAATCGCGGA TCAGCATGCC GCGGTGAATA
HydRick2   CGTGGAGCAA ATCC-TC-AA AAGGCATCTC AGTTCGGATT GTTCTCTGCA ACTCGAGAGC ATGAAGTC-G GAATCACTAG TAATCGCGGA TCAGCATGCC GCGGTGAATA
DioOBOD9   CGTGGAGCAA ATCC-CT-AA AAAGTATCTC AGTTCGGATT GCTCTCTGCA ACTCGAGAGC ATGAAGTC-G GAATCACTAG TAATCGCAGA TCAGCATGCT GCGGTGAATA
PrmCauda   CGTGGAGCAA ATCC-CT-AA AAAGTATCTC AGTTCGGATT GCTCTCTGCA ACTCGAGAGC ATGAAGTC-G GAATCACTAG TAATCGCAGA TCAGCATGCT GCGGTGAATA
Sprsmm01   CGTGGAGCAA ATCC-CT-AA AAAGTATCTC AGTTCGGATT GCTCTCTGCA ACTCGAGAGC ATGAAGTC-G GAATCACTAG TAATCGCAGA TCAGCATGCT GCGGTGAATA
DioDS124   CGTGGAGCAA ATCC-CT-AA AAAGTATCTC AGTTCGGATT GTTCTCTGCA ACTCGAGAGC ATGAAGTC-G GAATCACTAG TAATCGCGGA TCAGCATGCC GCGGTGAATA
EupOct01   CGTGGAGCAA ATCC-CT-AA AAAGTATCTC AGTTCGGATT GCTCTCTGCA ACTCGAGAGC ATGAAGTC-G GAATCACTAG TAATCGCAGA TCAGCATGCT GCGGTGAATA
NIES_425   CGTGGAGCAA ATCC-CT-AA AAAGTATCTC AGTTCGGATT GCTCTCTGCA ACTCGAGAGC ATGAAGTC-G GAATCACTAG TAATCGCAGA TCAGCATGCT GCGGTGAATA
NIES_577   CGTGGAGCAA ATCC-CT-AA AAAGTATCTC AGTTCGGATT GCTCTCTGCA ACTCGAGAGC ATGAAGTC-G GAATCACTAG TAATCGCGGA TCAGCATGCC GCGGTGAATA
UTEX2180   CGTGGAGCAA ATCC-CT-AA AAAGTATCTC AGTTCGGATT GTTCTCTGCA ACTCGAGAGC ATGAAGTC-G GAATCACTAG TAATCGCGGA TCAGCATGCC GCGGTGAATA
EVE_____   ---------- ---------- ---------- ---------- ---------- ---------- ---------- ---------- ---------- ---------- ----------
NIES_732   CGTGGAGCAA ATCC-CT-AA AAAGTATCTC AGTTCGATTT GTTCTCTGCA ACTCGAGAGC ATGAAGTC-G GAATCACTAG TAATCGCGGA TCCGCATGCC GCGGTGAATA
OrnTsIke   CGTGGAGCTA ATCA-TT-AA AAGGTATCTC AGTTCGGATT GCACTCTGCA ACTCGAGTGC ATGAAGTC-G GAATCGCTAG TAATCGCGGA TCAGCATGCC GCGGTGAATA
OrnTsBor   CGTGGAGCTA ATCA-TT-AA AAGGTATCTC AGTTCGGATT GCACTCTGCA ACTCGAGTGC ATGAAGTC-G GAATCGCTAG TAATCGCGGA TCAGCATGCC GCGGTGAATA
CprPolyt   CACGGAGCAA ATCC-CAAAA AAGATATCTC AGTTCGGATT GCACTCTGCA ACTCGAGTGC ATGAAGTC-G GAATCGCTAG TAATCGCGGA TCAGCATGCC GCGGTGAATA
```

```
           1321
RicRicIo   CGTTCTCGGG CCTTGTACAC ACTGCCCGTC ACGCCATGGG AGTTAGTTTT ACCTGAAGGT GGTGAGCTAA CGCAAGAGGC AGCCAACCAC GGTAAAATTA GC
RicLimo2   CGTTCTCGGG CCTTGTACAC ACTGCCCGTC ACGCCATGGG AGTTGGTTTT ACCTGAAGGT GGTGAGCTAA CGTAAGAGGC AGCCAACCAC GGTAAAATTA GC
RicFeli2   CGTTCTCGGG CCTTGTACAC ACTGCCCGTC ACGCCATGGG AGTTGGTTTT ACCTGAAGGT GGTGAGCTAA CGCAAGAGGC AGCCAACCAC GGTAAAATTA GC
RicProwa   CGTTCTCGGG CCTTGTACAC ACTGCCCGTC ACGCCATGGG AGTTGGTTTT ACCTGAAGGT GGTGAGCTAA CGCAAGAGGC AGCCAACCAC GGTAAAATTA GC
RicTyphi   CGTTCTCGGG CCTTGTACAC ACTGCCCGTC ACGCCATGGG AGTTGGTTTT ACCTGAAGGT GGTGAGCTAA CGCAAGAGGC AGCCAACCAC GGTAAAATTA GC
RicCono4   CGTTCTCGGG CCTTGTACAC ACTGCCCGTC ACGCCATGGG AGTTAGTTTT ACCTGAAGGT GGTGAGCTAA CGCAAGAGGC AGCCAACCAC GGTAAAATTA GC
RicEmpPa   CGTTCTCGGG CCTTGTACAC ACTGCCCGTC ACGCCATGGG AGTTGGTTTT ACCTGAAGGT GGTGCAGTAA CGCAAGAGGC AGCCAACCAC GGTAAAATTA GC
RicJapon   CGTTCTCGGG CCTTGTACAC ACTGCCCGTC ACGCCATGGG AGTTGGTTTT ACCTGAAGGT GGTGAGCTAA CGCAAGAGGC AGCCAACCAC GGTAAAATTA GC
RicCana2   CGTTCTCGGG CCTTGTACAC ACTGCCCGTC ACGCCATGGG AGTTGGTTTT ACCTGAAGGT GGTGAGCTAA CGCAAGAGGC AGCCAACCAC GGTAAAATTA GC
RicHelve   CGTTCTCGGG CCTTGTACAC ACTGCCCGTC ACGCCATGGG AGTTGGTTTT ACCTGAAGGT GGTGAGCTAA CGCAAGAGGC AGCCAACCAC GGTAAAATTA GC
RicAust3   CGTTCTCGGG CCTTGTACAC ACTGCCCGTC ACGCCATGGG AGTTGGTTTT ACCTGAAGGT GGTGAGCTAA CGCAAGAGGC AGCCAACCAC GGTAAAATTA GC
RicBell3   CGTTCTCGGG CCTTGTACAC ACTGCCCGTC ACGCCATGGG AGTTGGTTTT ACCTGAAGGT GGTGAGCTAA CGCAAGAGGC AGCCAACCAC GGTAAAATTA GC
RicAkar3   CGTTCTCGGG CCTTGTACAC ACTGCCCGTC ACGCCATGGG AGTTGGTTTT ACCTGAAGGT GGTGAGCTAA CGCAAGAGGC AGCCAACCAC GGTAGAATTA GC
TorTsuku   CGTTCTCGGG TCTTGTACAC ACTGCCCGTC ACGCCATGGG AGTTGGTTTT ACCTGAAGGT GGTGAGCTAA CGTAAGAGGC AGCCAACCCC GGTAAAATTA GC
TorTagoi   CGTTCTCGGG TTTTGTACAC ACTGCCCGTC ACGCCATGGG AGTTGGTTTT ACCTGAAGGT GGTGAGCTAA CGTAAGAGGC AGCCAACCAC GGTAAAATTA GC
HemMarg2   CGTTCTCGGG CCTTGTACAC ACTGCCCGTC ACGCCATGGG AGTTGGTTTT ACCTGAAGGT GGTGAGCTAA CGTAAGAGGC AGCCAACCAC GGTAAAATTA GC
HydRick1   CGTTCTCGGG CCTTGTACAC ACTGCCCGTC ACGCCATGGG AGTTGGTCTT ACCTGAAGGT GGTGAGCTAA CGTAAGAGGC AGCCAACCAC GGTAGAATTA GC
IchMulti   CGTTCTCGGG CCTTGTACAC ACTGCCCGTC ACGCCATGGG AGTTGGTTTT ACCTGAAGCT GGTGTGCTAA CGCAAGAGGC AGCCAACCAC GGTAGAATTA GC
BryRick1   CGTTCTCGGG CCTTGTACAC ACTGCCCGTC ACGCCATGGG AGTTGGTTCT ACCTGAAGGT GGCTAGCTAA CGCAAGAGGC AACCAACCGC GGTAGAATTA GC
BryRick2   CGTTCTCGGG CCTTGTACAC ACTGCCCGTC ACGCCATGGG AGTTGGTTTT ACCTGAAGGT GGCAAGCTAA CGTAAGAGGC AACCAACCAC GGTAGAATTA GC
BryRick3   CGTTCTCGGG CCTTGTACAC ACTGCCCGTC ACGCCATGGG AGTTGGTTCT ACCTGAAGGT GGCTAGCTAA CGCAAGAGGC AACCAACCAC GGTAGAATTA GC
MonFaveo   CGTTCTCGGG CCTTGTACAC ACTGCCCGTC ACGCCATGGG AGTTGGTTCT ACCTGAAGGT GGCTAGCTAA CGCAAGAGGC AACCAACCAC GGTAGAATTA GC
RicNepCi   CGTTCTCGGG CCTTGTACAC ACTGCCCGTC ACGCCATGGG AGTTGGTTTT ACCTGAAGGT GGTGAGCTAA CGTAAGAGGC AGCCAACCAC GGTAAAATTA GC
Kalahari   CGTTCTCGGG CCTTGTACAC ACTGCCCGTC ACGCCATGGG AGTTGGTTTT ACCCGAAGCT GGTGAGCTAA CGTAAGAGGC AGCCAACCAC GGTAAAATTA GC
T6025C27   CGTTCTCGGG CCTTGTACAC ACTGCCCGTC ACGCCATGGG AGTTGGTTTT ACCTGAAGCT GGTGAGCTAA CGCAAGAGGC AGCCAACCAC GGTAAAATTA GC
T6025C01   CGTTCTCGGG CCTTGTACAC ACTGCCCGTC ACGCCATGGG AGTTGGTTTT ACCCGAAGCT GGTGAGCTAA CGTAAGAGGC AGCCAACCGC GGTAAAATTA GC
WakWat77   CGTTCTCGGG CCTTGTACAC ACTGCCCGTC ACGCCATGGG AGTTGGTTTT ACCTGAAGCT GGTGAGCTAA CGTAAGAGGC AGCCAACCAC GGTAAAATTA GC
Waste214   CGTTCTCGGG CCTTGTACAC ACTGCCCGTC ACGCCATGGG AGTTGGTTTT ACCCGAAGCT GGTGAGCTAA CGTAAGAGGC AGCCAACCAC GGTAAAATTA GC
TaihAS94   CGTTCTCGGG CCTTGTACAC ACTGCCCGTC ACGCCATGGG AGTTGGTTTT ACCCGAAGCT GGTGAGCTAA CGTAAGAGGC AGTCAACCAC GGTAAAATTA GC
HydRick2   CGTTCTCGGG CCTTGTACAC ACTGCCCGTC ACGCCATGGG AGTTGGTTTT ACCTGAAGGT GGTGAGCTAA CGTAAGAGGC AGCCAACCAC GGTAGAATTA GC
DioOBOD9   CGTTCTCGGG CCTTGTACAC ACTGCCCGTC ACGCCATGGG AGTTGGTTTT ACCCGAAGCT GGTGAGCTAA CGTAAGAGGC AGCCAACCAC GGTAAAATTA GC
PrmCauda   CGTTCTCGGG CCTTGTACAC ACTGCCCGTC ACGCCATGGG AGTTGGTTTT ACCCGAAGCT GGTGAGCTAA CGTAAGAGGT AGCCAACCAC GGTAAAATTA GC
Sprsmm01   CGTTCTCGGG CCTTGTACAC ACTGCCCGTC ACGCCATGGG AGTTGGTTTT ACCCGAAGCT GGTGAGCTAA CGTAAGAGGC AGCCAACCAC GGTAAAATTA GT
DioDS124   CGTTCTCGGG CCTTGCACAC ACTGCCCGTC ACGCCATGGG AGTTGGTTTT ACCCGAAGCT GGTGAGCTAA CGTAAGAGGC AGCCAACCAC GGTAAAATTA GC
EupOct01   CGTTCTCGGG CCTTGTACAC ACTGCCCGTC ACGCCATGGG AGTTGGTTTT ACCCGAAGCT GGTGAGCTAA CGTAAGAGGC AGCCAACCAC GGTAAAATTA GC
NIES_425   CGTTCTCGGG CCTTGTACAC ACTGCCCGTC ACGCCATGGG AGTTGGTTTT ACCCGAAGCT GGTGAGCTAA CGTAAGAGGC AGCCAACCAC GGTAAAATTA GC
NIES_577   CGTTCTCGGG CCTTGTACAC ACTGCCCGTC ACGCCATGGG AGTTGGTTTT ACCCGAAGCT GGTGAGCTAA CGTAAGAGGC AGCCAACCAC GGTAAAATTA GC
UTEX2180   CGTTCTCGGG CCTTGTACAC ACTGCCCGTC ACGCCATGGG AGTTGGTTTT ACCCGAAGCT GGTGAGCTAA CGTAAGAGGC AGCCAACCAC GGTAAAATTA GC
EVE_____   ---------- ---------- ---------- ---------- ---------- ---------- ---------- ---------- ---------- ---------- --
NIES_732   CGTTCTCGGG CCTTGTACAC ACTGCCCGTC ACGCCATGGG AGTTGGTTTT ACCCGAAGCT GGTGAGCTAA CGCAAGAGGC AGCCAACCAC GGTAAAATTA GC
OrnTsIke   CGTTCTCGGG CCTTGTACAC ACTGCCCGTC GCACCATGGG AGTCAGTGGT ACCTGAAGTT GGTGAGCTAA CGTAAGAGGC AGCTAACCAC GGTAGAACTG GT
OrnTsBor   CGTTCTCGGG CCTTGTACAC ACTGCCCGTC GCACCATGGG AGTCAGTGGT ACCTGAAGTT GGTGAGCTAA CGTAAGAGGC AGCTAACCAC GGTAGAACTG GT
CprPolyt   CGTTCCCGGG CCTTGTACAC ACTGCCCGTC AAGCCATGGG AGTTAATGTT ACCTTAAGTT GGTGAGCTAA CGAAAGAGGC AGCCAACCAT GGTAAAATTA GT
```

murB (translated)

```
            1
RicMasM5    LKSFLIQNKQ KLPIKTFGAG SNIIIRDGGI EGVVIKLGQN FSNIEFIDN- HLI---VGSS CLNYNLAKFC QANAISGFEF LVGIPGTIGG GVAMNAGAYG SEFKDIIVQI
RicRicIo    LKSFLIQNKQ KLPIKTFGAG SNIIIRDGGI EGVVIKLGQN FSNIEFIDN- HLI---VGSS CLNYNLAKFC QANAISGFEF LVGIPGTIGG GVAMNAGAYG SEFKDIVVQI
RicConM7    LKSFLIQNKQ KLPIKTFGAG SNIIIRDGGI EGVVIKLGQN FSNIEFVDN- HLI---VGSS CLNYNLAKFC QANAISGFEF LVGIPGTIGG GVAMNAGAYG SEFKDIVVQI
RicJapYH    LKSFLIQNKQ KLPIKTFGAG SNIIIRDGGI EGVVIKLGQN FSNIEFIDN- HLI---VGSS CLNYNLAKFC QANAISGFEF LVGIPGTIGG GIAMNAGAYG SEFKDIVVQI
RicSloDC    LKSFLIQNKQ KLPIKTFGVG SNIIIRDGGI EGVVIKLGQN FSNIEFIDN- HLI---VGSS CLNYNLAKFC QANAISGFEF LVGIPGTIGG GVAMNAGAYG SEFKDIVVQI
RicParPo    LKSFLIQNKQ KLPIKTFGAG SNVIIRDGGI EGVVIKLGQN FSNIEFIDN- HLI---VGSS CLNYNLAKFC QANAISGFEF LVGIPGTIGG GVAMNAGAYG SEFKDIVVQI
RicRhipi    LKSFLIQNKQ KLPIKTFGAG SNIIIRDGGI EGVVIKLGQN FSNIEFIDN- HLI---VGSS CLNYNLAKFC QANAISGFEF LVGIPGTIGG GVAMNAGAYG SEFKDIIVQI
RicAmbly    LKSFLIQNKQ KLPIKTFGAG SNIIIRDGGI EGFVIKLGQN FSNIEFIDN- HLI---VGSS CLNYNLAKFC QANSISGFEF LVGIPGTIGG GVAMNAGAYG SEFKDIIVQI
RicPhili    LKSFLIQNKQ KLPIKTFGAG SNIIIRDGGI EGVVIKLGQN FSNIEFIDN- HLI---VGSS CLNYNLAKFC QANAISGFEF LVGIPGTIGG GVAMNAGAYG SEFKDIVVQI
RicAfrE5    LKSFLIQNKQ KLPIKTFGAG SNVIIRDGGI EGVVIKLGQN FSNIEFIDN- HLI---VGSS CLNYNLAKFC QANAISGFEF LVGIPGTIGG GVAMNAGAYG SEFKDIVVQI
RicMonta    LKSFLIQNKQ KLPIKTFGAG SNIIIRDGGI EGVVIKLGQN FSNIEFIDN- HLI---VGSS CLNYNLATFC QANAISGFEF LVGIPGTIGG GVAMNAGAYG SEFKDIIVQI
RicPeacR    LKSFLIQNKQ KLPIKTFGAG SNIIIRDGGI EGVVIKLGQN FSNIEFIDN- HLI---VGSS CLNYNLAKFC QANAISGFEF LVGIPGTIGG GVAMNAGAYG SEFKDIVVQI
RicTypWi    LKSFLIQNNK KLPITTFGSG SNIIIRDGGI EGVVIKLGKN FNNIEFLDN- HLI---VGSS CLNYNLAKFC QANAISGFEF LVGIPGTIGG GVVMNAGAYG SAFQDIIVQI
RicProME    LKSFLIQNKQ KLPITTFGSG SNIIIRDGGI EGVVIKLGQN FNKIEFLDN- HLI---VGSS CLNYNLARFC QANAISGFEF LVGIPGTIGG GVIMNAGAYG SAFQDIIVQV
RicAuC_c    LANFLIQNKQ KLPITTFGAG SNIIIRDGGI EGVVIKLGQN FSNIEFIDN- HLI---VGSS CLNYNLAKFC QANAISGFEF LVGIPGTIGG GVAMNAGAYG SEFKDIIVKI
RicAkarH    LASFLIQNRQ KLPITTFGAG SNIIIRDGGI EGVVIKLGQN FSNIEFIDN- HLI---VGSS CLNYNLAKFC QANDISGFEF LVGIPGTIGG GVAMNAGAYG SEFKDIIVRI
RicFel_c    LASFLIQNKQ KLPITTFGAG SNIIIRDGGI EGVTIKLGQN FSNIEFIDE- HLI---VGSS CLNYNLAKFC QANAITGFEF LVGIPGTIGG GVVMNAGAYD SEFKDIIVKI
RicCaMcK    LASFLRQNKQ KLPITTFGAG SNIIIRDGGI EGVTIKLGQS FSNIDFIDDN HLV---VGSS CLNYNLAKFC QANAISGFEF LVGIPGTIGG GAAMNAGAYG SEFKDIIVRI
RicCa410    LASFLRQNKQ KLPITTFGAG SNIIIRDGGI EGVTIKLGQS FSNIDFIDDN HLV---VGSS CLNYNLAKFC QANAISGFEF LVGIPGTIGG GAAMNAGAYG SEFKDIIVRI
RicBO389    LASFLVQNKQ KLPITTFGAG SNIIIRDGGI EGVTIKLGQN FSNIGFTDDG HLI---VGSS CLNFSLAKFC QVNAISGFEF LVGIPGTIGG GVAMNAGAYG CEFKDILVRI
RicBR3_c    LASFLVQNKQ KLPITTFGAG SNIIIRDGGI EGVTIKLGQN FSNIDFTDDG HLI---VGSS CLNFSLAKFC QVNAISGFEF LVGIPGTIGG GVAMNAGAYG CEFKDILVRI
OriTsuBo    LSCFLKNLQF DIQITVLGAG SNLLIRDNGI DGVTIKLGRS FNEINFVKNN HYNIISVGAG TLNYDVAKFC LQHNLGGLEF LVGIPGTIGG GIAMNAGAYG QEFKDVVYSV
OriTsuIk    LSYFLANLQS NIKITVLGAG SNLLIRDNGI DGVTIKLGRS FNEINFVKNN HYNIISVGAG TLNYDVAKFC LQHNLGGLEF LVGIPGTIGG GIAMNAGAYG QEFKDVVYSV
NIES_425    LKTFLKEHNN RFPITVIGAG SNLIIRDKGI EGVVIKLGRN FTDIQFVDNN -FI--AAGAG CLNSSLAKFC LVNAITGFEF LIGIPGTVGG GMAMNAGSYG CEFKDIVASV
UTEX2180    LKTFLKENNN RFPITVIGAG SNLIIRDKGV EGIVIKLGRN FTDIQFIDNN -LI--VAGAG CLNSNLAKFC LVNSIAGFEF LVGIPGTVGG GVAMNAGSYG REFKDIVASV
NIES_577    LKTFFKGNND KFPITVIGAG SNLIIRDKGI EGVVIKLGRN FTDIQFVDNN -LI--VAGTG CLNSSLAKFC LVNSITGFEF LIGIPGTVGG GVAMNAGSYG REFKDIVASV
NIES_866    LKTFFKGNND KFPITVIGAG SNLIIRDKGI EGVVIKLGRN FTDIQFVDNN -LI--VAGAG CLNSSLAKFC LVNSITGFEF LIGIPGTVGG GVAMNAGSYG REFKDIVASV
EVE_____    LKTFFKGNND KFPITVIGAG SNVIIRDKGI EGVVIKLGRN FTDVQFVDNN -LI--VAGAG CLNSSLAKFC LVNSITGFEF LIGIPGTVGG GVAMNAGSYG REFKDIVASV
```

```
            111
RicMasM5    EAIDFAGNFL TFTNEEIGFK YRSNNLPKNL IILKAVFKIN KGDS-ENILL RMNEINNARS STQPIKERTG GSTFANPAG- RKSWELIDKV GLRGYRIGGA SMSELHCNFM
RicRicIo    EAIDFAGNFL TFTNEEIGFK YRSNNLPKNL IILKVIFKIN KGDS-ENILL RMNEINNARS STQPIKERTG GSTFANPEG- CKSWELIDKA GLRGYRIGGA SMSELHCNFM
RicConM7    EAIDFAGNFL TFTNEEIGFK YRSNNLPKNL IILKAVFKIN KGDS-ENILL RMNEIKNARS STQPIKERTG GSTFANPEG- RKSWELIDKA GLRGYRIGGA SMSEFHCNFM
RicJapYH    EAIDFAGNFL TFTNEEIGFK YRSNNLPKNL IILKAVFKIN KGDS-ENILL RMNEINNARS STQPIKERTG GSTFANPEG- RKSWELIDKA GLRGYRIGGA SMSELHCNFM
RicSloDC    EAIDFAGNFL TFTNEEIGFK YRSNNLPKNL IILKAVFKIN KGDS-ENILL RMNEINNARS STQPIKERTG GSTFANPEG- RKSWELIDKA GLRGYRIGGA SMSELHCNFM
RicParPo    EAIDFAGNFL TFTNEEIGFK YRSNNLPKNL IILKAVFKIN KGNS-ENILL RMNEINNARS STQPIKERTG GSTFANPEG- RKSWELIDKA GLRGYRIGGA SMSELHCNFM
RicRhipi    EAIDFAGNFL TFTNEEIGFK YRSNNLPKNL IILKAVFKIN KGDS-ENILL RMNEINNARS STQPIKERTG GSTFANPEG- RKSWELIDKA GLRGYRIGGA SMSELHCNFM
RicAmbly    EAIDFAGNFL TFTNEEIGFK YRSNNLPKNL IILKAVFKIN KGDS-ENILL RMNEINNARL STQPIKERTG GSTFANPEG- RKSWELIDKA GLRGYRIGGA SMSELHCNFM
RicPhili    EAIDFAGNFL TFTNEEIGFK YRSNNLPKNL IILKVVFKIN KGDS-ENILL RMNEINNARS STQPIKERTG GSTFANPEG- CKSWELIDKA GLRGYRIGGA SMSELHCNFM
RicAfrE5    EAIDFAGNFL TFTNEEIGFK YRSNNLPKNL IILKAVFKIN KGDS-ENILL RMNEIHNARS STQPIKERTG GSTFANPEG- RKSWELIDKA GLRGYRIGGA SMSELHCNFM
RicMonta    EAIDFAGNVL TFTNEEIGFK YRSNNLPKNL IILKAVFKIN KGDS-ENILL RMNEINNARS STQPIKERTG GSTFANPEG- RKSWELIDKA GLRGYRIGGA SMSELHCNFM
RicPeacR    EAIDFAGNFL TFTNEAIGFK YRSNNLPKNL IILKVVFKIN KGDS-ENILL RMNEINNARS STQPIKERTG GSTFANPEG- HKSWELIDKA GLRGYRIGGA SMSELHCNFM
RicTypWi    EALDFLGNFL TFTNKEIGFK YRGNNLPKDL ILLKAIFKAN KGDS-QNILL KMNKINTTRS STQPIKERTG GSTFKNPVG- CKSWELIDKA GLRGYRIGGA SMSELHCNFM
RicProME    EALDFSGNFL TFTNKEIGFK YRGNNLPKDL ILLKAVFKVN KGDS-QNILL KMNKINNTRS STQPIKERTG GSTFINPEG- RKSWELIDKA GLRGYRIGGA SISELHCNFM
RicAuC_c    EAIDFAGNFR TFTNEESGFK YRGNNLPKDL IILKAVFKVN KGDS-ENILL RMNEINNARS STQPIKERTG GSTFANPEG- LKSWELIDKA GLRGYRIGGA SMSELHCNFM
RicAkarH    EAIDFAGNFR TFTNAESGFK YRGNNLPKDL IILKAVFKVN KGDS-ENILL RMNEINNTRS LTQPIKERTG GSTFANPEG- LKSWELIDKA GLRGYRIGGA SMSELHCNFM
RicFel_c    EAIDFAGNFL TFTNEEIGFK YRSNNLPKDL IILKAVFKVN KGDS-ENILL RMNEINNARS ATQPIKERTG GSTFANPEG- LKSWELIDKA GLRGYRIGGA SMSELHCNFM
RicCaMcK    EAIDFAGNFL TFTNEEIGFK YRSNNLPKNL IILKAIFKVN KGDS-ENILL RMNEINATRS RTQPIKERTG GSTFANPEGG LKSWQLIDKA GLRGYRIGGA SVSELHCNFM
RicCa410    EAIDFAGNFL TFTNEEIGFK YRSNNLPKNL IILKAIFKVN KGDS-ENILL RMNEIKATRS RTQPIKERTG GSTFANPEG- LKSWQLIDKA GLRGYRIGGA SVSELHCNFM
RicBO389    EAIDFAGNFR TFTNEEIGFK YRGNNLPKDL IILKAVFKVN KGNS-EDILA RMNEINAARS STQPIKERTG GSTFANPEG- FKSWQLIDKA GLRGYRIGDA SISELHCNFM
RicBR3_c    EAIDFAGNFR TFTNEEIGFK YRGNNLPKDL IILKAVFKVN KGNS-EDILA RMNEINAARS STQPIKERTG GSTFANPEG- FKSWQLIDKA GLRGYRIGDA SISELHCNFM
OriTsuBo    EALDRLGNKH IFLSKDLNFE YRQCIVNGFL IFTKTNLICY N-DSKTSISQ KLQKIQTVRK LTQPINQKTA GSAFRNTNN- YKAWQLIDKV GLRGHSIGGA QVSNLHCNFL
OriTsuIk    EALDRSGNKH IFLSKDLNFE YRQCIVNGFL IFTKTNLICY N-DSKPSISQ KLQKIQTVRK LTQPINQKTA GSAFRNTNN- YKAWQLIDKV GLRGHSIGGA QVSNLHCNFL
NIES_425    EVLDKEGNLV ILDIDQIGFS YRANSLPKDL IFTRVFFKAK NKEDINKIKQ KMDEISDIRS SSQPVSEKTG GSTFANPEG- FRAWELIDKA GLRKTRVGGA CMSEMHCNFM
UTEX2180    EVLDTEGNLI IIPVDRIGFS YRTNSLPKDL IFTRVFFQAK NKEDTNKIKQ KMNEISAIRS SSQPVSEKTG GSTFANPKG- FKAWELIDKA GLRGVRVGGA CMSEMHCNFM
NIES_577    EVLDKEGNLI IIPIDQIGFS YRANSLPKDL IFTRVFFQAK NKEDVNKIKQ KMDEISAIRS ASQPINERTG GSTFANPEG- FRAWELIDKA GLRNARVGGA CMSEMHCNFM
NIES_866    EVLDKEGNLI IIPIDQIGFS YRANSLPKDL IFTRVFFQAK NKEDVNKIKQ KMDEISAIRS ASQPINERTG GSTFANPEG- FRAWELIDKA GLRSARVGGA CMSEMHCNFM
EVE______   EVLDKEGDLI IIPIHQIGFS YRANSLPKDL IFTRVFFQAK NK-DVNKIIQ KMDEISAIRS ASQPINERTG GSTFANPEG- FRAWELIDKA GLRSARVGGA CMSEMHCNFM
```

```
           221
RicMasM5  INNGDAT
RicRicIo  INNGDAT
RicConM7  INNGDAT
RicJapYH  INNGDAT
RicSloDC  INNGDAT
RicParPo  INNGDAT
RicRhipi  INNGDAT
RicAmbly  INNGDAT
RicPhili  INNGDAT
RicAfrE5  INNGDAT
RicMonta  INNGDAT
RicPeacR  INNGDAT
RicTypWi  INNGNAT
RicProME  INNGNAT
RicAuC_c  INNGDAT
RicAkarH  INNGDAT
RicFel_c  INNGDAT
RicCaMcK  INNGDAT
RicCa410  INNGDAT
RicBO389  INNGNAT
RicBR3_c  INNGNAT
OriTsuBo  INNGNAT
OriTsuIk  INNGNAT
NIES_425  INDNNAT
UTEX2180  INANNAT
NIES_577  INYNNAT
NIES_866  INYNNAT
EVE_____  INYNNAT
```

ddlB (translated)

```
           1
RicMasM5   MH--KYQTHW VEHSIVKILS S--------- ---------- ---------- ----AGKKYI ALMAGGMSAE REVSLVSSEG VSKALIELGY SVTFIDMGAD ITVRLQEIK-
RicMaA_c   MHKYKYQTHW VEHSIVKILS S--------- ---------- ---------- ----TGKKYI ALMAGGMSAE REVSLVSSEG VSKALIELGY RVTFIDMGAD ITVRLQEIK-
RicRicIo   VH--KYQTHW VEHSIVKILS S--------- ---------- ---------- ----TGKKHI ALMAGGMSAE REVSLVSSEG VSKALIELGY RVTFIDMGAD IAVRLQEIK-
RicConM7   VH--KYQTHW VEHSIVKILS S--------- ---------- ---------- ----TGKKHI ALMAGGMSAE REVSLVSSEG VSKALIELGY RVTFIDMGAD IAVRLQEIK-
RicJapYH   MH--KYQTHW VEHSIVKILS S--------- ---------- ---------- ----TGKKHI ALMAGGMSAE REVSLVSSEG VSKALIELGY RVTFIDMGAD IAVRLQEIK-
RicSloDC   VH--KYQTHW VEHSIVKILS S--------- ---------- ---------- ----TGKKHI ALMAGGMSAE REVSLVSSEG VSKALIELGY RVTFIDMGAD IAVRLQEIK-
RicParPo   VH--KYQTHW VEHSIVKILS S--------- ---------- ---------- ----TGKKHI ALMAGGMSAE REVSLVSSEG VSKALIELGY RVTFIDMGAD IAVRLQEIK-
RicRhipi   MH--KYQTHW VEHSIVKILS S--------- ---------- ---------- ----TGKKYI ALMAGGMSAE REVSLVSSEG VSKALIELGY RVTFIDMGAD ITVRLQEIK-
RicHeilo   MH--KYQTHW VEHSIVKILS S--------- ---------- ---------- ----TGKKHI ALMAGGMSAE REVSLVSSEG VSKALIELGY RVTFIDMGAD IAVRLQERK-
RicAmbly   MH--KYQTHW VEHSIVKILS S--------- ---------- ---------- ----TGKKHI ALMAGGMSAE REVSLVSSKG VSKALIELGY RVTFIDMGVD IAVRLQEIK-
RicPhili   VH--KYQTHW VEHSIVKILS S--------- ---------- ---------- ----TGKKHI ALMAGGMSAE REVSLVSSEG VSKALIELGY RVTFIDMGAD IAVRLQEIK-
RicAfrE5   VH--KYQTHW VEHSIVKILS S--------- ---------- ---------- ----TGKKHI ALMVGGMSAE REVSLVSSEG VSKALIELGY RVTFIDMGAD IAVRLQEIK-
RicMonta   MH--KYQTHW VEHSIVKILS S--------- ---------- ---------- ----TGKKHI ALMAGGMSAE REVSLVSSEG VSKALIELGY RVTFIDMGAD IAVRLQEIK-
RicPeacR   VH--KYQTHW VEHSIVKILS S--------- ---------- ---------- ----TGKKHI ALMAGGMSAE REVSLVSSEG VSKALIELGY RVTFIDMGAD IAVRLQEIK-
RicTypWi   MN--KYQTHL LEHSVVKILS G--------- ---------- ---------- ----TGKKHI ALVAGGMSAE REVSLISSVG VSKALIELGY KVTFIDMGAD IAVKLQEIN-
RicProME   MH--KYQTHF VEHSVVKILS S--------- ---------- ---------- ----TGKKHI ALVAGGMSAE REVSLVSSAG VSKALIELGY KVTFVDMGAD IAVKLQEIN-
RicAuC_c   M---KYQTHW IEHSVVKILS T--------- ---------- ---------- ----TGKKHI ALVAGGMSAE REVSLVSSEG VHKALIELGY KVTFIDMGAD IAVKLQKIK-
RicAkarH   M---QYQTHW IEHSVVKILS T--------- ---------- ---------- ----TGKNHI ALVAGGMSAE REVSLVSSEG VQQALIALGY KVTFIDMGAD IAVKLQEIK-
RicFel_c   MN--KYQTHW VEHSEVKILS T--------- ---------- ---------- ----TGKKHI ALVAGGMSAE REVSLVSSEG VSKALIELGY KVTFIDMGAD IAVKLQEIK-
RicCaMcK   MH--QYQIHW VEHSEVKILS NIVVRLEYKE RGVKSITNRR ARHDAVSESK SIDYSGKKHI ALVAGGMSAE REVSLVSSKG VSKALIALGY KVTFIDMGAD IAFKLQEIK-
RicCa410   MH--QYQIHW VEHSEVKILS NIVVRLEYKE RGVKSITNRR ATHDTVSESK SIDYSGKKHI ALVAGGMSAE REVSLVSSKG VSKALIALGY KVTFIDMGAD IAFKLQEIK-
RicBO389   MH--KYQTHW VESSEIKILS D--------- ---------- ---------- ----KGKKHI ALVAGGMSAE REVSLISAEG VGKALIEAGY KVTFIDMGAD IAVKLHEIK-
RicBR3_c   MH--KYQTHW VESSEIKILS D--------- ---------- ---------- ----KGKKHI ALVAGGMSAE REVSLISAEG VGKALIEAGY KVTFIDMGAD ITVKLHEIK-
OriTsuBo   M--------- ---------- ---------- ---------- ---------- ---------- -------SAE REVSLMSNDN VQAALISNGY QVTRIDVGQD IAVKLSEITP
OriTsuIk   LLGKKAYSFQ RHNSVVESIT S--------- ---------- ---------- ----SGKKHV VILYGGMSAE REVSLMSNDN VQAALISNGY QVTRIDVGQD IAVKLSEITR
NIES_425   MSNGNFKTVY KEKSLIVELD SLRELTDMKL IDFVNLQGKP I-----EGFV SGRNKIKKHV VVIGGGMSAE REVSYMSSNG IVRSIIELGH HVTFVDMGAD IAVVLLNLK-
UTEX2180   MSNGNFKTVY KEKSLIVELD SLNKLADIKL IDFVQVQAKP I-----EKFI ARRSSAKKHV MVIGGGMSAE REVSYMSSNG IVRSLIELGH HVTFVDMGAD IAVVLLNLK-
NIES_577   MANGNFKTVY KEKSLIVEFD SLSKLGDMKL IDFVKLEGKS I-----ENFV AKRNTIKKHV VVIGGGMSAE REVSYMSSNG IVRSIIELGH HVTFVDMGAD IAVLLLNLK-
EVEmarge   MANGNFKTVY KEKSLIVEFD SFSKLGDMKL IDFVKLEGKS I-----ENFV AKRNTIKKHV VVIGGGMSAE REVSYMSSNG IVRSIIELGH HVTFVDMGAD IAVLLLNLK-
NIES_866   MANGNFKTVY KEKSLIVEFD SLSKLGDIKL IDFVKLEGKS I-----ENFV AKINTIKKHV VVIGGGMSAE REVSYMSSNG IVRSIIELGH HVTFVDMGAD IAVLLLNLK-
```

```
             111
RicMasM5   PDIVFNCLHG TYGEDGCLPG LLNIMRIPYT HSGMLSSALA FNKIHSRSWF LTNNINMAES IVVNKSDNIK NDPMKRPYVI KPLTQGSSIG VEVIFAEDNF NFADYDFPYG
RicMaA_c   PDIVFNCLHG TYGEDGCLPG LLNIMRIPYT HSGMLSSALA FNKIHSRSWF LTNNINMAES IVVNKSDNIK NDPMKRPYVI KPLTQGSSIG VEVIFAEDDF NFADYDFPYG
RicRicIo   PDIVFNCLHG TYGEDGGLPG LLNIMRIPYT HSGVLSSALA FDKIHSRIWF LTNNINMAES IVVNKSDNIK NDPMKRPYVI KPLTQGSSIG VEVIFAEDDF NFADYDFPYG
RicConM7   PDIVFNCLHG TYGEDGCLPG LLNIMRIPYT HSGVLSSALA FDKIHSRIWF LTNNINMAEG IVVNKSDNIK NDPMKRPYVI KPLTQGSSIG VEVIFAEDDF NFADYDFPYG
RicJapYH   PDIVFNCLHG TYGEDGCLPG LLNIMRIPYT HSGVLSSALA FNKIHSRSWF LTNNINMAES IVVNKSDNIK NDPMKRPYVI KPLTQGSSIG VEVIFAEDDF NFADYDFPYG
RicSloDC   PDIVFNCLHG TYGEDGCLPG LLNIMRIPYT HSGVLSSALA FDKIHSRIWF LTNNINMAES IVVNKSDNIK NDPMKRPYVI KPLTQGSSIG VEVIFAEDDF NFADYDFPYG
RicParPo   PDIVFNCLHG TYGEDGCLPG LLNIMRIPYT HSGVLSSALA FDKIHSRIWF LTNNINMAES IVVNKSDNIK NDPMKRPYVI KPLTQGSSIG VEVIFAEDDF NFADYDFPYG
RicRhipi   PDIVFNCLHG TYGEDGCLPG LLNIMRIPYT HSGMLSSALA FNKIHSRSWF LTNNINMAES IVVNKSDNIK NDPMKRPYVI KPLTQGSSIG VEVIFAEDDF NFADYDFPYG
RicHeilo   PDIVFNCLHG TYGEDGCLPG LLNIMRIPYT HSGVLSSALA FNKIHSRSWF LTNNINMAES IVVNKSDNIK NDPMKRPYVI KPLTQGSSIG VEVIFAEDDF NFADYDFPYG
RicAmbly   PDIVFNCLHG TYGEDGCLPG LLNIMRIPYT HSGVLSSVLA FDKIHSRRWF LTNNINMAES IVVNKSDNIK NDPMKRPYVI KPITQGSSIG VEVIFAEDDF NFADYDFPYG
RicPhili   PDIVFNCLHG TYGEDGCLPG LLNIMRIPYT HSGVLSSALA FDKIHSRIWF LTNNINMAES IVVNKSDNIK NDPMKRPYVI KPLTQGSSIG VEVIFAEDDF NFADYDFPYG
RicAfrE5   PDIVFNCLHG TYGEDGCLPG LLNIMRIPYT HSGMLSSALA FDKIHSRIWF LTNNINMAES IVVNKSDNIK NDPMKRPYVI KPLTQGSSIG VEVIFAEDDF NFADYDFPYG
RicMonta   PDIVFNCLHG TYGEDGCLPG LLNIMRIPYT HSGVLSSALA FDKIHSRSWF LTHNINMAES IVVNKSDNIK NDPMKRPYVI KPLAQGSSIG VEVIFAEDDF NFADYDFPYG
RicPeacR   PDIVFNCLHG TYGEDGCLPG LLNIMRIPYT HSGVLSSALA FNKIHSRIWF LTNNINMAES IVVNKSDNIK NDPMKRPYVI KPLAQGSSIG VEVIFAEDDF NFADYDFPYG
RicTypWi   PDIVFNCLHG TYGEDGCLPG LLNIMRIPYT HSGVLSSALA FDKIHSRSCF LKNNINMADS IVVSKSDHIN TDPMKRPYVI KPLKQGSSIG VEVIFEEDDF HFIDYDFPYG
RicProME   PDIVFNCLHG TYGEDGCLPG LLNIMRIPYT HSGLLSSALA FDKIHFRSCV LKNNINIADS IVVSKSDHIN TDPMKRPYVI KPLKQGSSIG VEVIFEEDDF NFTDYDFPYG
RicAuC_c   PDIVFNCLHG TYGEDGCLPG LLNIMRIPYT HSGVLSSALA FDKIHSSSWF VTNNINTAES IVVSKSDNIK TDPMKRPYVI KPLTQGSSIG VEVIFEEDDF NFADYDFPYG
RicAkarH   PDIVFNCLHG TYGEDGCLPG LLNIMRIPYT HSGVLSSALA FNKIHSSSWF FANSINTAES IVVSKSYNIN TDPMKRPYVI KPLTQGSSIG VEVIFEEDDF NFADYDFPYG
RicFel_c   PDIVFNCLHG TYGEDGCLPG LLNIMRIPYT HSGVLSSALA FDKIHSRSWF LTNNINMAES IIVNKSDNIK SDPVKRPYVI KPLTQGSSIG VEVIFEEDDF NFADYNFPYG
RicCaMcK   PDIVFNCLHG TYGEDGCLSG LLNIMRIPYT HSGVLSSALA FDKIYSRSWF LTNNINMAES IVVNKSDNIK IEPMKRPYVI KPITQGSSIG IEVIFEEDDF NFANYDFPYG
RicCa410   PDIVFNCLHG TYGEDGCLSG LLNIMGIPYT HSGVLSSALA FDKIYSRSWF LTNNINMAES IVVNKSDNIK IEPMKRPYVI KPITQGSSIG IEVIFEEDDF NFANYDFPYG
RicBO389   PDIVFNCLHG TYGEDGCLPG LLNIMRIPYT HSGVLASSLA FDKVHSRSWF LTNNINMAES IVISKGDNIK TDPIKRPYVI KPFTQGSSIG VEVIFEEDDF NFANYDFPYG
RicBR3_c   PDIVFNCLHG TYGEDGCLPG LLNIMRIPYT HSGVLASSLA FDKVHSRSWF LTNNINMAES IVISKGDNIK TDPIKRPYVI KPFTQGSSIG VEVIFEEDDF NFANYDFPYG
OriTsuBo   PYTVFNCLVG TYGEDGCIPG LLNIMNIPYT HSGVKTSAAA FDKQIAKTIL QCYKIKTPAS ITINSNDNVI NDPILRPYVI KPLQQGSSIG VKIVHLEDKF LFKDYKFQFG
OriTsuIk   PYTVFNCLVG TYGEDGCIPG LLNIMNIPYT HSGVKTSAAA FDKQIAKTIL QCYKIKTPAS ITINSNDNVI DDPILRPYVI KPLQQGSSIG VKIVHLEDKF LFKDYKFQFG
NIES_425   PDVVYNALHG TYGEDGCLPG LLNIMRIPYT GPGVLASAIA LNKRKSCEIF RSTGINIPKS KLIKKSDGYT KDPIKRPYVI KPLSQGSSVG VQVIFPEDIF SFGDYDFPYG
UTEX2180   PDVVYNALHG TYGEDGCLPG LLNIMRIPYT GPGVLASAIA LNKRKSCEIF QATGIKIPES KLIKKSDGYT KDPIKRPYVI KPLAQGSSVG VQVIFPDDIF SFGNYDFPYG
NIES_577   PDVVYNALHG TYGEDGCLPG LLNIMRIPYT GPGVLASAIA LNKRKSYEIF RSTGIKIPES KLIKKSDGYT KDPIKRPYVI KPLSQGSSVG VQVVFPEDIF SFSDYAFPYG
EVEmarge   PDVVYNALHG TYGEDGCLPG LLNIMRIPYT GPGVLASAIA LNKRKSYEIF RSTGIKIPES KLIKKSDGYT KDPIKRPYVI KPLSQGSSVG VQVVFPEDIF SFSDYDFPYG
NIES_866   PDVVYNALHG TYGEDGCLPG LLNIMRIPYT GPGVLASAIA LNKRKSYEIF RSTGIKIPES KLIKKSDGYT KDPIKRPYVI KPLSQGSSVG VQVVFPEDIF SFSDYDFPYG
```

```
           221
RicMasM5   DQVIIEQYIK GR--ELQVAV LNGKALGVLE IKLLKN-RFY DYETKYTEGF ADHLCPAPLP ANLYEKLLIE SEKIYKTMNC -KGPARAEFI LEEQTNKLYA LELNTHPGMT
RicMaA_c   DQVIIEQYIK GR--ELQVAL LNGKALGVLE IQLLKN-RFY DYETKYTEGF ADHLCPAPLP ANLYEKLLIE SEKIYKTMNC -KGPARAEFI LEEQTNKLYA LELNTHPGMT
RicRicIo   DQVIIEQYIK GR--ELQVAV LNGKALGALE IKLLKN-RFY DYETKYTAGF ADHLCPAPLP ANLYEKLLIE SEKIYKTMNC -KGPARAEFI LEEQTNKLYA LEINTHPGMM
RicConM7   DQVIIEQYIK GR--ELQVAV LNGKALGALE IKLLKN-RFY DYETKYTEGF ADHLCPAPLP ANLYEKLLIE SEKIYKTMNC -KGPARAEFI LEEQTNKLYA LEINTHPGMM
RicJapYH   DQVIIEQYIK GR--ELQVAV LNGKALGALE IKLLKN-RFY DYETKYTEGF ADHLCPVPLP ANLYEKLLIE SEKIYKTMNC -KGPARAEFI LEEQTNKLYA LEINTHPGMT
RicSloDC   DQVIIEQYIK GR--ELQVVV LNGKALGALE IKLLKN-RFY DYETKYTEGF ADHLCPAPLP ANLYEKLLIE SEKIYKTMNC -KGPARAEFI LEEQTNKLYA LEINTHPGMM
RicParPo   DQVIIEQYIK GR--ELQVAV LNGKALGALE IKLLKN-RFY DYETKYTEGF ADHLCPAPLP ANLYEKLLIE SEKIYKTMNC -KGPARAEFI LEEQTNKLYA LEINTHPGMM
RicRhipi   DQVIIEQYIK GR--ELQVAV LNGKALGVLE IKLLKN-RFY DYETKYTEGF ADHLCPAPLP ANLYEKLLIE SEKIYKTMNC -KGPARAEFI LEEQTNKLYV LELNTHPGMT
RicHeilo   DQVIIEQYIK GR--ELQVAV LNGKALGALE IKLLKN-RFY DYETKYTEGF ADHLCPVPLP ANLYEKLLIE SEKIYKTMNC -KGPARAEFI LEEQTNKLYA LEINTHPGMT
RicAmbly   DQVIIEQYIK GR--ELQVAV LNGKALGVLE IKLLKN-RFY DYETKYTEGF ADHLCPAPLP ANLYEKLLIE SEKIYKTINC -KGPARAEFI LEEQTNKLYA LEINTHPGMT
RicPhili   DQVIIEQYIK GR--ELQVAV LNGKALGALE IKLLKN-RFY DYETKYTAGF ADHLCPAPLP ANLYEKLLIE SEKIYKTMNC -KGPARAEFI LEEQTNKLYA LEINTHPGMM
RicAfrE5   DQVIIEQYIK GR--ELQVAV LNGKALGALE IKLLKN-RFY DYETKYTEGF ADHLCPAPLP ANLYEKLLIE SEKIYKTMNC -KGPARAEFI LEEQTNKLYA LEINTHPGMT
RicMonta   DQVIIEQYIK GR--ELQVVV LNGKALGALE IKLLKN-RFY DYETKYTEGF ADHLCPAPLP ANLHEKLLIE SEKIYKTMNC -KGPARAEFI LEEQTNKLYA LEINTHPGMT
RicPeacR   DQVIIEQYIK GQGRELQVAV LNGKALGALE IKLLKN-RFY DYETKYTEGF ADHLCPAPLP ANLYEKLLIE SEKIYKTMNC -KGPARAEFI LEEQTNKLYA LEINTHPGMM
RicTypWi   EDIIIEQYIQ GQ--ELQVAL LNGKALGVLE IKLLKN-RFY DYETKYNKGF AKHVCPAQLP ANLYKKLLIE SEKIYKTINC -KGPVRAEFI LEEQTNKLYV LEINTHPGMT
RicProME   EDIIIEQYIQ GQ--ELQVAL LNGKALGVLE IKLLKN-RFY DYETKYNEGF AEHVCPAPLP ANLYKKLLIE SEKIYKTINC -KGPVRAEFI LEEQTNKLYV LEINTHPGMT
RicAuC_c   DQVVIERYIK GR--ELQVAV LNSKALGVLE IKLLKN-RFY DYATKYTEGF AEHLCPAPLP ANLYEKLLIA SEKIYKTMNC -KGPARAEFI LEEQTNKLYA LEINTHPGMT
RicAkarH   DQVVIERYIK GR--EFQVAV LNSKALGALE IKLIKN-RFY DYETKYTEGF AEHLCPAPLH ANLYEKLLIE SEKIYKTMNC -KGPARAEFI LEEQTNKLYA LEINTHPGMT
RicFel_c   YQVIIEQYIK GR--ELQVAV LNGKALGALE IKLLKN-RFY DYETKYTEGF AEHLCPAPLP TNLYEKLLVE SEKIYKTMNC -KGPARAEFI LEEQTNKLYA LEINTHPGMT
RicCaMcK   DQVIIEKYIK GR--ELQVAV LNGKALGVLE IKLLKN-RFY DYETKYTEGF AEHLCPAPIP TNLYDKLLIE SEKIYKTMNC -KGPARVEFL LEDQTNKLYA LEINTHPGMT
RicCa410   DQVIIEKYIK GR--ELQVAV LNGKALGVLE IKLLKN-RFY DYETKYTEGF AEHLCPAPIP TNLYHKLLIE SEKIYKTMNC -KGPARVEFL LEDQTNKLYA LEINTHPGMT
RicBO389   DEVIIEKYIK GR--ELQVAI LNGKALGALE IKLLKN-RFY DYETKYTEGF AEHLCPAPLP TDIYDKLLKE SEKIYNTMNC -KGAARAEFI LEDGTNKLYA LEINTHPGMT
RicBR3_c   DEVIIEKYIK GR--ELQVAI LNGKALGALE IKLLKN-RFY DYETKYTEGF AEHLCPAPLP TDIYDKLLKE SEKIYNTMNC -KGAARVEFI LEDGTNKLYA LEINTHPGMT
OriTsuBo   NKVLVEQYIK GR--ELQVAL LDGKVVGILE IKMLKDKIFY DYQSKYSPGF AEHIIPPRLP VNTVNQIMNI AEKVYKIFDC -RGPCRLECI LSDIDNEVYV IELNTHPGMT
OriTsuIk   NKVLVEQYIK GR--ELQVAL LDGQVVGILE IKMLKDKIFY DYQSKYSPGF AEHIIPPMLP VNTVNQVINI AEKVYKIFDC -RGPCRLECI LSDIDNEVYV IELNTHPGMT
NIES_425   DEILVEEYIK GR--EMQVAV LNGRAIGILE IKLLKNKRFY DYETKYTEGF SEHLLPAPVS SEIYNNMKAM AEKACIALDC VTGMIRVEMI YSSEKNALYM LEVNTHPGMT
UTEX2180   DEILVEEYIK GR--EMQVAV LNGRAIGVLE IKLLKNKRFY DYETKYTEGF AEHLLPAPVS SEMYNNIKDL AEKACSVLDC VTGMIRVEMI YSPERNALYM LEVNTHPGMT
NIES_577   DEILIEEYIK GR--EMQVAV LNGRAIGVLE IKLLKNKRFY DYETKYTEGF AEHLLPAPVS LEMYNNIKAL AEKACSVLDC VTGMIRVEMI YSSEKNELYM LEVNTHPGMT
EVEmarge   DEILIEEYIK GR--EMQVAV LNGRAIGVLE IKLLKNKRFY DYETKYTEGF AEHLLPAPVS LEMYNNIKAL AEKACSVLDC VTGMIRVEMI YSSEKNELYM LEVNTHPGMT
NIES_866   DEILIEEYIK GR--EMQVAV LNGRAIGVLE IKLLKNKRFY DYETKYTEGF AEHLLPAPVS LEMYNNIKAL AEKACSVLDC VTGMIRVEMI CSSEKNELYM LEVNTHPGMT
```

```
               331
RicMasM5  PLSIVPEIAA YAGINFTNLI EEIIKTASFE S
RicMaA_c  PLSIVPEIAA YAGINFTNLI EEIIKTASFE S
RicRicIo  SLSIVPEIAA YAGINFTNLI EEIIKTASFE S
RicConM7  PLSIVPEIAA YAGINFTNLI EEIIKTASFE S
RicJapYH  PLSIVPEIAA YAGINFTNLI EEIIKTASFE S
RicSloDC  PLSIVPEIAA YAGINFTNLI EEIIKTASFE S
RicParPo  SLSIVPEIAA YAGINFTNLI EEIIKTASFE S
RicRhipi  PLSIVPEIAA YAGINFTNLI EEIIKTASFE S
RicHeilo  PLSIVPEIAA YAGINFTNLI EEIIKTASFE S
RicAmbly  PLSIVPEIAA YVGINFTNLI EEIIKTASFE S
RicPhili  SLSIVPEIAA YAGINFTNLI EEIIKTASFE S
RicAfrE5  PLSIVPEIAA YAGINFTNLI EEIIKMASFE S
RicMonta  PLSIVPEIAA YAGINFTNLI EEIIKTASFE S
RicPeacR  PLSIVPEIAA YAGINFTNLI EEIIKTASFE S
RicTypWi  PLSIVPEIAA YAGISFTNLI EEIIKMASFE S
RicProME  PLSIVPEIAA YAGINFTNLI EEIIKMASFE S
RicAuC_c  LLSIVPEIAA YAGINFTNLI EEIIKTASFE S
RicAkarH  PLSIVPEIAA YAGINFTNLI AEIIKTASFE S
RicFel_c  PLSIVPEIAA YAGINFTNLI EEIIKTASFE S
RicCaMcK  PLSIVPEIAA YAGINFTNLI EEIIKAASFE S
RicCa410  PLSIVPEIAA YAGINFTNLI EEIIKAASFE S
RicBO389  PLSIVPEIAA YHGIDFVNLI EEILKTASFE S
RicBR3_c  PLSIVPEIAA YHGIDFVNLI EEILKTASFE S
OriTsuBo  SLSSYPEIAQ YYGISFNELV ERILITAKCD -
OriTsuIk  SLSSYPEIAQ YYGISFNELV ERILITAKCD -
NIES_425  PLSICPEIAT LENMSYKDLV KEILEEARFE -
UTEX2180  PLSICPEIAT LENISYKDLV KEILEEAKFE -
NIES_577  QLSICPEIAT LENMSYKDLV KEILEEAKFE -
EVEmarge  PLSICPEIAT LENMSYKDLV KEILEEAKFE -
NIES_866  PLSICPEIAT LENMSYKGLV KEILEEAKFE -
```

Combined alignment of murB and ddlB (translated)

```
           1
RicMasM5   LKSFLIQNKQ KLPIKTFGAG SNIIIRDGGI EGVVIKLGQN FSNIEFIDN- HLI---VGSS CLNYNLAKFC QANAISGFEF LVGIPGTIGG GVAMNAGAYG SEFKDIIVQI
RicMaA_c   LKSFLIQNKQ KLPIKTFGAG SNIIIRDGGI EGVVIKLGQN FSNIEFIDN- HLI---VGSS CLNYNLAKFC QANAISGFEF LVGIPGTIGG GVAMNAGAYG SEFKDIIVQI
RicRicIo   LKSFLIQNKQ KLPIKTFGAG SNIIIRDGGI EGVVIKLGQN FSNIEFIDN- HLI---VGSS CLNYNLAKFC QANAISGFEF LVGIPGTIGG GVAMNAGAYG SEFKDIVVQI
RicConM7   LKSFLIQNKQ KLPIKTFGAG SNIIIRDGGI EGVVIKLGQN FSNIEFVDN- HLI---VGSS CLNYNLAKFC QANAISGFEF LVGIPGTIGG GVAMNAGAYG SEFKDIVVQI
RicJapYH   LKSFLIQNKQ KLPIKTFGAG SNIIIRDGGI EGVVIKLGQN FSNIEFIDN- HLI---VGSS CLNYNLAKFC QANAISGFEF LVGIPGTIGG GIAMNAGAYG SEFKDIVVQI
RicSloDC   LKSFLIQNKQ KLPIKTFGVG SNIIIRDGGI EGVVIKLGQN FSNIEFIDN- HLI---VGSS CLNYNLAKFC QANAISGFEF LVGIPGTIGG GVAMNAGAYG SEFKDIVVQI
RicParPo   LKSFLIQNKQ KLPIKTFGAG SNVIIRDGGI EGVVIKLGQN FSNIEFIDN- HLI---VGSS CLNYNLAKFC QANAISGFEF LVGIPGTIGG GVAMNAGAYG SEFKDIVVQI
RicRhipi   LKSFLIQNKQ KLPIKTFGAG SNIIIRDGGI EGVVIKLGQN FSNIEFIDN- HLI---VGSS CLNYNLAKFC QANAISGFEF LVGIPGTIGG GVAMNAGAYG SEFKDIIVQI
RicHeilo   LKSFLIQNKQ KLPIKTFGAG SNIIIRDGGI EGVVIKLGQN FSNIEFIDN- HLI---VGSS CLNYNLAKFC QANAISGFEF LVGIPGTIGG GVAMNAGAYG YEFKDIVVQI
RicAmbly   LKSFLIQNKQ KLPIKTFGAG SNIIIRDGGI EGFVIKLGQN FSNIEFIDN- HLI---VGSS CLNYNLAKFC QANSISGFEF LVGIPGTIGG GVAMNAGAYG SEFKDIIVQI
RicPhili   LKSFLIQNKQ KLPIKTFGAG SNIIIRDGGI EGVVIKLGQN FSNIEFIDN- HLI---VGSS CLNYNLAKFC QANAISGFEF LVGIPGTIGG GVAMNAGAYG SEFKDIVVQI
RicAfrE5   LKSFLIQNKQ KLPIKTFGAG SNVIIRDGGI EGVVIKLGQN FSNIEFIDN- HLI---VGSS CLNYNLAKFC QANAISGFEF LVGIPGTIGG GVAMNAGAYG SEFKDIVVQI
RicMonta   LKSFLIQNKQ KLPIKTFGAG SNIIIRDGGI EGVVIKLGQN FSNIEFIDN- HLI---VGSS CLNYNLATFC QANAISGFEF LVGIPGTIGG GVAMNAGAYG SEFKDIIVQI
RicPeacR   LKSFLIQNKQ KLPIKTFGAG SNIIIRDGGI EGVVIKLGQN FSNIEFIDN- HLI---VGSS CLNYNLAKFC QANAISGFEF LVGIPGTIGG GVAMNAGAYG SEFKDIVVQI
RicTypWi   LKSFLIQNNK KLPITTFGSG SNIIIRDGGI EGVVIKLGKN FNNIEFLDN- HLI---VGSS CLNYNLAKFC QANAISGFEF LVGIPGTIGG GVVMNAGAYG SAFQDIIVQI
RicProME   LKSFLIQNKQ KLPITTFGSG SNIIIRDGGI EGVVIKLGQN FNKIEFLDN- HLI---VGSS CLNYNLARFC QANAISGFEF LVGIPGTIGG GVIMNAGAYG SAFQDIIVQV
RicAuC_c   LANFLIQNKQ KLPITTFGAG SNIIIRDGGI EGVVIKLGQN FSNIEFIDN- HLI---VGSS CLNYNLAKFC QANAISGFEF LVGIPGTIGG GVAMNAGAYG SEFKDIIVKI
RicAkarH   LASFLIQNRQ KLPITTFGAG SNIIIRDGGI EGVVIKLGQN FSNIEFIDN- HLI---VGSS CLNYNLAKFC QANDISGFEF LVGIPGTIGG GVAMNAGAYG SEFKDIIVRI
RicFel_c   LASFLIQNKQ KLPITTFGAG SNIIIRDGGI EGVTIKLGQN FSNIEFIDE- HLI---VGSS CLNYNLAKFC QANAITGFEF LVGIPGTIGG GVVMNAGAYD SEFKDIIVKI
RicCaMcK   LASFLRQNKQ KLPITTFGAG SNIIIRDGGI EGVTIKLGQS FSNIDFIDDN HLV---VGSS CLNYNLAKFC QANAISGFEF LVGIPGTIGG GAAMNAGAYG SEFKDIIVRI
RicCa410   LASFLRQNKQ KLPITTFGAG SNIIIRDGGI EGVTIKLGQS FSNIDFIDDN HLV---VGSS CLNYNLAKFC QANAISGFEF LVGIPGTIGG GAAMNAGAYG SEFKDIIVRI
RicBO389   LASFLVQNKQ KLPITTFGAG SNIIIRDGGI EGVTIKLGQN FSNIGFTDDG HLI---VGSS CLNFSLAKFC QVNAISGFEF LVGIPGTIGG GVAMNAGAYG CEFKDILVRI
RicBR3_c   LASFLVQNKQ KLPITTFGAG SNIIIRDGGI EGVTIKLGQN FSNIDFTDDG HLI---VGSS CLNFSLAKFC QVNAISGFEF LVGIPGTIGG GVAMNAGAYG CEFKDILVRI
OriTsuBo   LSCFLKNLQF DIQITVLGAG SNLLIRDNGI DGVTIKLGRS FNEINFVKNN HYNIISVGAG TLNYDVAKFC LQHNLGGLEF LVGIPGTIGG GIAMNAGAYG QEFKDVVYSV
OriTsuIk   LSYFLANLQS NIKITVLGAG SNLLIRDNGI DGVTIKLGRS FNEINFVKNN HYNIISVGAG TLNYDVAKFC LQHNLGGLEF LVGIPGTIGG GIAMNAGAYG QEFKDVVYSV
NIES_425   LKTFLKEHNN RFPITVIGAG SNLIIRDKGI EGVVIKLGRN FTDIQFVDNN -FI--AAGAG CLNSSLAKFC LVNAITGFEF LIGIPGTVGG GMAMNAGSYG CEFKDIVASV
UTEX2180   LKTFLKENNN RFPITVIGAG SNLIIRDKGV EGIVIKLGRN FTDIQFIDNN -LI--VAGAG CLNSNLAKFC LVNSIAGFEF LVGIPGTVGG GVAMNAGSYG REFKDIVASV
NIES_577   LKTFFKGNND KFPITVIGAG SNLIIRDKGI EGVVIKLGRN FTDIQFVDNN -LI--VAGTG CLNSSLAKFC LVNSITGFEF LIGIPGTVGG GVAMNAGSYG REFKDIVASV
EVE_____   LKTFFKGNND KFPITVIGAG SNVIIRDKGI EGVVIKLGRN FTDVQFVDNN -LI--VAGAG CLNSSLAKFC LVNSITGFEF LIGIPGTVGG GVAMNAGSYG REFKDIVASV
NIES_866   LKTFFKGNND KFPITVIGAG SNLIIRDKGI EGVVIKLGRN FTDIQFVDNN -LI--VAGAG CLNSSLAKFC LVNSITGFEF LIGIPGTVGG GVAMNAGSYG REFKDIVASV
```

```
          111
RicMasM5  EAIDFAGNFL TFTNEEIGFK YRSNNLPKNL IILKAVFKIN KGDS-ENILL RMNEINNARS STQPIKERTG GSTFANPAG- RKSWELIDKV GLRGYRIGGA SMSELHCNFM
RicMaA_c  EAIDFAGNFL TFTNEEIGFK YRSNNLPKNL IILKAVFKIN KGDS-ENILL RMNEINNARS STQPIKERTG GSTFANPAG- RKSWELIDKV GLRGYRIGGA SMSELHCNFM
RicRicIo  EAIDFAGNFL TFTNEEIGFK YRSNNLPKNL IILKVIFKIN KGDS-ENILL RMNEINNARS STQPIKERTG GSTFANPEG- CKSWELIDKA GLRGYRIGGA SMSELHCNFM
RicConM7  EAIDFAGNFL TFTNEEIGFK YRSNNLPKNL IILKAVFKIN KGDS-ENILL RMNEIKNARS STQPIKERTG GSTFANPEG- RKSWELIDKA GLRGYRIGGA SMSEFHCNFM
RicJapYH  EAIDFAGNFL TFTNEEIGFK YRSNNLPKNL IILKAVFKIN KGDS-ENILL RMNEINNARS STQPIKERTG GSTFANPEG- RKSWELIDKA GLRGYRIGGA SMSELHCNFM
RicSloDC  EAIDFAGNFL TFTNEEIGFK YRSNNLPKNL IILKAVFKIN KGDS-ENILL RMNEINNARS STQPIKERTG GSTFANPEG- RKSWELIDKA GLRGYRIGGA SMSELHCNFM
RicParPo  EAIDFAGNFL TFTNEEIGFK YRSNNLPKNL IILKAVFKIN KGNS-ENILL RMNEINNARS STQPIKERTG GSTFANPEG- RKSWELIDKA GLRGYRIGGA SMSELHCNFM
RicRhipi  EAIDFAGNFL TFTNEEIGFK YRSNNLPKNL IILKAVFKIN KGDS-ENILL RMNEINNARS STQPIKERTG GSTFANPEG- RKSWELIDKA GLRGYRIGGA SMSELHCNFM
RicHeilo  EAIDFAGNFL TFTNEEIGFK YRSNNLPKNL IILKAVFKIN KGDS-ENILL RMNEINNARS STQPIKERTG GSTFANPEG- RKSWELIDKA GLRGYRIGGA SMSELHCNFM
RicAmbly  EAIDFAGNFL TFTNEEIGFK YRSNNLPKNL IILKAVFKIN KGDS-ENILL RMNEINNARL STQPIKERTG GSTFANPEG- RKSWELIDKA GLRGYRIGGA SMSELHCNFM
RicPhili  EAIDFAGNFL TFTNEEIGFK YRSNNLPKNL IILKVVFKIN KGDS-ENILL RMNEINNARS STQPIKERTG GSTFANPEG- CKSWELIDKA GLRGYRIGGA SMSELHCNFM
RicAfrE5  EAIDFAGNFL TFTNEEIGFK YRSNNLPKNL IILKAVFKIN KGDS-ENILL RMNEIHNARS STQPIKERTG GSTFANPEG- RKSWELIDKA GLRGYRIGGA SMSELHCNFM
RicMonta  EAIDFAGNVL TFTNEEIGFK YRSNNLPKNL IILKAVFKIN KGDS-ENILL RMNEINNARS STQPIKERTG GSTFANPEG- RKSWELIDKA GLRGYRIGGA SMSELHCNFM
RicPeacR  EAIDFAGNFL TFTNEAIGFK YRSNNLPKNL IILKVVFKIN KGDS-ENILL RMNEINNARS STQPIKERTG GSTFANPEG- HKSWELIDKA GLRGYRIGGA SMSELHCNFM
RicTypWi  EALDFLGNFL TFTNKEIGFK YRGNNLPKDL ILLKAIFKAN KGDS-QNILL KMNKINTTRS STQPIKERTG GSTFKNPVG- CKSWELIDKA GLRGYRIGGA SMSELHCNFM
RicProME  EALDFSGNFL TFTNKEIGFK YRGNNLPKDL ILLKAVFKVN KGDS-QNILL KMNKINNTRS STQPIKERTG GSTFINPEG- RKSWELIDKA GLRGYRIGGA SISELHCNFM
RicAuC_c  EAIDFAGNFR TFTNEESGFK YRGNNLPKDL IILKAVFKVN KGDS-ENILL RMNEINNARS STQPIKERTG GSTFANPEG- LKSWELIDKA GLRGYRIGGA SMSELHCNFM
RicAkarH  EAIDFAGNFR TFTNAESGFK YRGNNLPKDL IILKAVFKVN KGDS-ENILL RMNEINNTRS LTQPIKERTG GSTFANPEG- LKSWELIDKA GLRGYRIGGA SMSELHCNFM
RicFel_c  EAIDFAGNFL TFTNEEIGFK YRSNNLPKDL IILKAVFKVN KGDS-ENILL RMNEINNARS ATQPIKERTG GSTFANPEG- LKSWELIDKA GLRGYRIGGA SMSELHCNFM
RicCaMcK  EAIDFAGNFL TFTNEEIGFK YRSNNLPKNL IILKAIFKVN KGDS-ENILL RMNEINATRS RTQPIKERTG GSTFANPEGG LKSWQLIDKA GLRGYRIGGA SVSELHCNFM
RicCa410  EAIDFAGNFL TFTNEEIGFK YRSNNLPKNL IILKAIFKVN KGDS-ENILL RMNEIKATRS RTQPIKERTG GSTFANPEG- LKSWQLIDKA GLRGYRIGGA SVSELHCNFM
RicBO389  EAIDFAGNFR TFTNEEIGFK YRGNNLPKDL IILKAVFKVN KGNS-EDILA RMNEINAARS STQPIKERTG GSTFANPEG- FKSWQLIDKA GLRGYRIGDA SISELHCNFM
RicBR3_c  EAIDFAGNFR TFTNEEIGFK YRGNNLPKDL IILKAVFKVN KGNS-EDILA RMNEINAARS STQPIKERTG GSTFANPEG- FKSWQLIDKA GLRGYRIGDA SISELHCNFM
OriTsuBo  EALDRLGNKH IFLSKDLNFE YRQCIVNGFL IFTKTNLICY N-DSKTSISQ KLQKIQTVRK LTQPINQKTA GSAFRNTNN- YKAWQLIDKV GLRGHSIGGA QVSNLHCNFL
OriTsuIk  EALDRSGNKH IFLSKDLNFE YRQCIVNGFL IFTKTNLICY N-DSKPSISQ KLQKIQTVRK LTQPINQKTA GSAFRNTNN- YKAWQLIDKV GLRGHSIGGA QVSNLHCNFL
NIES_425  EVLDKEGNLV ILDIDQIGFS YRANSLPKDL IFTRVFFKAK NKEDINKIKQ KMDEISDIRS SSQPVSEKTG GSTFANPEG- FRAWELIDKA GLRKTRVGGA CMSEMHCNFM
UTEX2180  EVLDTEGNLI IIPVDRIGFS YRTNSLPKDL IFTRVFFQAK NKEDTNKIKQ KMNEISAIRS SSQPVSEKTG GSTFANPKG- FKAWELIDKA GLRGVRVGGA CMSEMHCNFM
NIES_577  EVLDKEGNLI IIPIDQIGFS YRANSLPKDL IFTRVFFQAK NKEDVNKIKQ KMDEISAIRS ASQPINERTG GSTFANPEG- FRAWELIDKA GLRNARVGGA CMSEMHCNFM
EVE_____  EVLDKEGDLI IIPIHQIGFS YRANSLPKDL IFTRVFFQAK NK-DVNKIIQ KMDEISAIRS ASQPINERTG GSTFANPEG- FRAWELIDKA GLRSARVGGA CMSEMHCNFM
NIES_866  EVLDKEGNLI IIPIDQIGFS YRANSLPKDL IFTRVFFQAK NKEDVNKIKQ KMDEISAIRS ASQPINERTG GSTFANPEG- FRAWELIDKA GLRSARVGGA CMSEMHCNFM
```

```
         221
RicMasM5  INNGDATAKD LEDLGDFVRQ KVFEDSGVKL EWEIKRIGRH P--------- ------MH-- KYQTHWVEHS IVKILSS--- ---------- ---------- ----------
RicMaA_c  INNGDATAKD LEDLGDFVRQ KVFEDSGVKL EWEIKRIGRH P--------- ------MHKY KYQTHWVEHS IVKILSS--- ---------- ---------- ----------
RicRicIo  INNGDATAKD LEDLGNFVQQ KVCEDSGVKL EWEIKRIGRH P--------- ------VH-- KYQTHWVEHS IVKILSS--- ---------- ---------- ----------
RicConM7  INNGDATAKD LEDLGDFVRQ KVCEDSGVKL EWEIKRIGRH P--------- ------VH-- KYQTHWVEHS IVKILSS--- ---------- ---------- ----------
RicJapYH  INNGDATAKD LEDLGDFVRQ KVFEDSGVKL EWEIKRIGRH S--------- ------MH-- KYQTHWVEHS IVKILSS--- ---------- ---------- ----------
RicSloDC  INNGDATAKD LEDLGDFVRQ KVCEDSGVKL EWEIKRIGRH P--------- ------VH-- KYQTHWVEHS IVKILSS--- ---------- ---------- ----------
RicParPo  INNGDATAKD LEDLGDFVRQ KVCEDSGVKL EWEIKRIGRH P--------- ------VH-- KYQTHWVEHS IVKILSS--- ---------- ---------- ----------
RicRhipi  INNGDATAKD LEDLGDFVRQ KVFEDSGVKL EWEIKRIGRH P--------- ------MH-- KYQTHWVEHS IVKILSS--- ---------- ---------- ----------
RicHeilo  INNGDATAKD LEDLGDFVRQ KVFEDSGVKL EWEIKRIGRH P--------- ------MH-- KYQTHWVEHS IVKILSS--- ---------- ---------- ----------
RicAmbly  INNGDATAKD LEDLGDFVRQ KVFEDSGVQL EWEIKRIGRH P--------- ------MH-- KYQTHWVEHS IVKILSS--- ---------- ---------- ----------
RicPhili  INNGDATAKD LEDLGNFVQQ KVCEDSGVKL EWEIKRIGRH P--------- ------VH-- KYQTHWVEHS IVKILSS--- ---------- ---------- ----------
RicAfrE5  INNGDATAKD LEDLGDFVRQ KVCEDSGVKL EWEIKRIGRH P--------- ------VH-- KYQTHWVEHS IVKILSS--- ---------- ---------- ----------
RicMonta  INNGDATAKD LEDLGDFVRQ KVFEDSGVKL EWEIKRIGRY P--------- ------MH-- KYQTHWVEHS IVKILSS--- ---------- ---------- ----------
RicPeacR  INNGDATAKD LEDLGDFVRQ KVCEDSGVKL EWEIKRIGRH P--------- ------VH-- KYQTHWVEHS IVKILSS--- ---------- ---------- ----------
RicTypWi  INNGNATAKD LEDLGNFVRQ KVFEDSGVEL NWEIKRIGKY V--------- ------MN-- KYQTHLLEHS VVKILSG--- ---------- ---------- ----------
RicProME  INNGNATAKD LEDLGNFVRQ KVFEDSGVEL NWEIKRIGKY V--------- ------MH-- KYQTHFVEHS VVKILSS--- ---------- ---------- ----------
RicAuC_c  INNGDATAKD LEDLADFVRQ KVFEDSGVEL KWEIKRIGQC G--------- ------M--- KYQTHWIEHS VVKILST--- ---------- ---------- ----------
RicAkarH  INNGDATSKD LEDLGDFVRQ KVFEDSGVEL KWEIKIIGKY V--------- ------M--- QYQTHWIEHS VVKILST--- ---------- ---------- ----------
RicFel_c  INNGDATAKD LEDLGDFVRQ KVFEDSGVEL KWEIKRLGRY V--------- ------MN-- KYQTHWVEHS EVKILST--- ---------- ---------- ----------
RicCaMcK  INNGDATAKD LEDLGNFVRQ NVFEDSGVKL NWEIKRIGKY V--------- ------MH-- QYQIHWVEHS EVKILSNIVV RLEYKERGVK SITNRRARHD AVSESKSIDY
RicCa410  INNGDATAKD LEDLGNFVRQ NVFEDSGVKL NWEIKRIGKY V--------- ------MH-- QYQIHWVEHS EVKILSNIVV RLEYKERGVK SITNRRATHD TVSESKSIDY
RicBO389  INNGNATAKE LEDLGNFVQQ KVFEDSGIKL NWEIKRIGKV SSRAWLDHGI QRKIIKMH-- KYQTHWVESS EIKILSD--- ---------- ---------- ----------
RicBR3_c  INNGNATAKE LEDLGNFVQQ KVFEDSGIKL NWEIKRIGKV SSR-GLTTG- ------MH-- KYQTHWVESS EIKILSD--- ---------- ---------- ----------
OriTsuBo  INNGNATASD IENLGELIRK NVFDHTGITL EWEIKIVGKK SL-------- ------M--- ---------- ---------- ---------- ---------- ----------
OriTsuIk  INNGNATASD IENLGELIRK KVFDHTGITL EWEIKIVGKK SL-------- ------LLGK KAYSFQRHNS VVESITS--- ---------- ---------- ----------
NIES_425  INDNNATATD MENLGELVRQ KVKENSGVEL QWEIKRIGKL ---------- ------MSNG NFKTVYKEKS LIVELDSLRE LTDMKLIDFV NLQGKPI--- --EGFVSGRN
UTEX2180  INANNATATD MENLGELVKQ KVKENSGIEL QWEIKRIGKL ---------- ------MSNG NFKTVYKEKS LIVELDSLNK LADIKLIDFV QVQAKPI--- --EKFIARRS
NIES_577  INYNNATAAD MENLGELVRQ KVKENSGIEL QWEIKRIGRE ---------- ------MANG NFKTVYKEKS LIVEFDSLSK LGDMKLIDFV KLEGKSI--- --ENFVAKRN
EVE_____  INYNNATAAD MENLGELVRQ KVKENSGIEL QWEIKRIGRE ---------- ------MANG NFKTVYKEKS LIVEFDSFSK LGDMKLIDFV KLEGKSI--- --ENFVAKRN
NIES_866  INYNNATAAD MENLGELVRQ KVKENSGIEL QWEIKRIGRE ---------- ------MANG NFKTVYKEKS LIVEFDSLSK LGDIKLIDFV KLEGKSI--- --ENFVAKIN
```

```
        331
RicMasM5   AGKKYIALMA GGMSAEREVS LVSSEGVSKA LIELGYSVTF IDMGADITVR LQEIK-PDIV FNCLHGTYGE DGCLPGLLNI MRIPYTHSGM LSSALAFNKI HSRSWFLTNN
RicMaA_c   TGKKYIALMA GGMSAEREVS LVSSEGVSKA LIELGYRVTF IDMGADITVR LQEIK-PDIV FNCLHGTYGE DGCLPGLLNI MRIPYTHSGM LSSALAFNKI HSRSWFLTNN
RicRicIo   TGKKHIALMA GGMSAEREVS LVSSEGVSKA LIELGYRVTF IDMGADIAVR LQEIK-PDIV FNCLHGTYGE DGGLPGLLNI MRIPYTHSGV LSSALAFDKI HSRIWFLTNN
RicConM7   TGKKHIALMA GGMSAEREVS LVSSEGVSKA LIELGYRVTF IDMGADIAVR LQEIK-PDIV FNCLHGTYGE DGCLPGLLNI MRIPYTHSGV LSSALAFDKI HSRIWFLTNN
RicJapYH   TGKKHIALMA GGMSAEREVS LVSSEGVSKA LIELGYRVTF IDMGADIAVR LQEIK-PDIV FNCLHGTYGE DGCLPGLLNI MRIPYTHSGV LSSALAFNKI HSRSWFLTNN
RicSloDC   TGKKHIALMA GGMSAEREVS LVSSEGVSKA LIELGYRVTF IDMGADIAVR LQEIK-PDIV FNCLHGTYGE DGCLPGLLNI MRIPYTHSGV LSSALAFDKI HSRIWFLTNN
RicParPo   TGKKHIALMA GGMSAEREVS LVSSEGVSKA LIELGYRVTF IDMGADIAVR LQEIK-PDIV FNCLHGTYGE DGCLPGLLNI MRIPYTHSGV LSSALAFDKI HSRIWFLTNN
RicRhipi   TGKKYIALMA GGMSAEREVS LVSSEGVSKA LIELGYRVTF IDMGADITVR LQEIK-PDIV FNCLHGTYGE DGCLPGLLNI MRIPYTHSGM LSSALAFNKI HSRSWFLTNN
RicHeilo   TGKKHIALMA GGMSAEREVS LVSSEGVSKA LIELGYRVTF IDMGADIAVR LQERK-PDIV FNCLHGTYGE DGCLPGLLNI MRIPYTHSGV LSSALAFNKI HSRSWFLTNN
RicAmbly   TGKKHIALMA GGMSAEREVS LVSSKGVSKA LIELGYRVTF IDMGVDIAVR LQEIK-PDIV FNCLHGTYGE DGCLPGLLNI MRIPYTHSGV LSSVLAFDKI HSRRWFLTNN
RicPhili   TGKKHIALMA GGMSAEREVS LVSSEGVSKA LIELGYRVTF IDMGADIAVR LQEIK-PDIV FNCLHGTYGE DGCLPGLLNI MRIPYTHSGV LSSALAFDKI HSRIWFLTNN
RicAfrE5   TGKKHIALMV GGMSAEREVS LVSSEGVSKA LIELGYRVTF IDMGADIAVR LQEIK-PDIV FNCLHGTYGE DGCLPGLLNI MRIPYTHSGM LSSALAFDKI HSRIWFLTNN
RicMonta   TGKKHIALMA GGMSAEREVS LVSSEGVSKA LIELGYRVTF IDMGADIAVR LQEIK-PDIV FNCLHGTYGE DGCLPGLLNI MRIPYTHSGV LSSALAFDKI HSRSWFLTHN
RicPeacR   TGKKHIALMA GGMSAEREVS LVSSEGVSKA LIELGYRVTF IDMGADIAVR LQEIK-PDIV FNCLHGTYGE DGCLPGLLNI MRIPYTHSGV LSSALAFNKI HSRIWFLTNN
RicTypWi   TGKKHIALVA GGMSAEREVS LISSVGVSKA LIELGYKVTF IDMGADIAVK LQEIN-PDIV FNCLHGTYGE DGCLPGLLNI MRIPYTHSGV LSSALAFDKI HSRSCFLKNN
RicProME   TGKKHIALVA GGMSAEREVS LVSSAGVSKA LIELGYKVTF VDMGADIAVK LQEIN-PDIV FNCLHGTYGE DGCLPGLLNI MRIPYTHSGL LSSALAFDKI HFRSCVLKNN
RicAuC_c   TGKKHIALVA GGMSAEREVS LVSSEGVHKA LIELGYKVTF IDMGADIAVK LQKIK-PDIV FNCLHGTYGE DGCLPGLLNI MRIPYTHSGV LSSALAFDKI HSSSWFVTNN
RicAkarH   TGKNHIALVA GGMSAEREVS LVSSEGVQQA LIALGYKVTF IDMGADIAVK LQEIK-PDIV FNCLHGTYGE DGCLPGLLNI MRIPYTHSGV LSSALAFNKI HSSSWFFANS
RicFel_c   TGKKHIALVA GGMSAEREVS LVSSEGVSKA LIELGYKVTF IDMGADIAVK LQEIK-PDIV FNCLHGTYGE DGCLPGLLNI MRIPYTHSGV LSSALAFDKI HSRSWFLTNN
RicCaMcK   SGKKHIALVA GGMSAEREVS LVSSKGVSKA LIALGYKVTF IDMGADIAFK LQEIK-PDIV FNCLHGTYGE DGCLSGLLNI MRIPYTHSGV LSSALAFDKI YSRSWFLTNN
RicCa410   SGKKHIALVA GGMSAEREVS LVSSKGVSKA LIALGYKVTF IDMGADIAFK LQEIK-PDIV FNCLHGTYGE DGCLSGLLNI MGIPYTHSGV LSSALAFDKI YSRSWFLTNN
RicBO389   KGKKHIALVA GGMSAEREVS LISAEGVGKA LIEAGYKVTF IDMGADIAVK LHEIK-PDIV FNCLHGTYGE DGCLPGLLNI MRIPYTHSGV LASSLAFDKV HSRSWFLTNN
RicBR3_c   KGKKHIALVA GGMSAEREVS LISAEGVGKA LIEAGYKVTF IDMGADITVK LHEIK-PDIV FNCLHGTYGE DGCLPGLLNI MRIPYTHSGV LASSLAFDKV HSRSWFLTNN
OriTsuBo   ---------- ---SAEREVS LMSNDNVQAA LISNGYQVTR IDVGQDIAVK LSEITPPYTV FNCLVGTYGE DGCIPGLLNI MNIPYTHSGV KTSAAAFDKQ IAKTILQCYK
OriTsuIk   SGKKHVVILY GGMSAEREVS LMSNDNVQAA LISNGYQVTR IDVGQDIAVK LSEITRPYTV FNCLVGTYGE DGCIPGLLNI MNIPYTHSGV KTSAAAFDKQ IAKTILQCYK
NIES_425   KIKKHVVVIG GGMSAEREVS YMSSNGIVRS IIELGHHVTF VDMGADIAVV LLNLK-PDVV YNALHGTYGE DGCLPGLLNI MRIPYTGPGV LASAIALNKR KSCEIFRSTG
UTEX2180   SAKKHVMVIG GGMSAEREVS YMSSNGIVRS LIELGHHVTF VDMGADIAVV LLNLK-PDVV YNALHGTYGE DGCLPGLLNI MRIPYTGPGV LASAIALNKR KSCEIFQATG
NIES_577   TIKKHVVVIG GGMSAEREVS YMSSNGIVRS IIELGHHVTF VDMGADIAVL LLNLK-PDVV YNALHGTYGE DGCLPGLLNI MRIPYTGPGV LASAIALNKR KSYEIFRSTG
EVE_____   TIKKHVVVIG GGMSAEREVS YMSSNGIVRS IIELGHHVTF VDMGADIAVL LLNLK-PDVV YNALHGTYGE DGCLPGLLNI MRIPYTGPGV LASAIALNKR KSYEIFRSTG
NIES_866   TIKKHVVVIG GGMSAEREVS YMSSNGIVRS IIELGHHVTF VDMGADIAVL LLNLK-PDVV YNALHGTYGE DGCLPGLLNI MRIPYTGPGV LASAIALNKR KSYEIFRSTG
```

```
           441
RicMasM5   INMAESIVVN KSDNIKNDPM KRPYVIKPLT QGSSIGVEVI FAEDNFNFAD YDFPYGDQVI IEQYIKGR-- ELQVAVLNGK ALGVLEIKLL KN-RFYDYET KYTEGFADHL
RicMaA_c   INMAESIVVN KSDNIKNDPM KRPYVIKPLT QGSSIGVEVI FAEDDFNFAD YDFPYGDQVI IEQYIKGR-- ELQVALLNGK ALGVLEIQLL KN-RFYDYET KYTEGFADHL
RicRicIo   INMAESIVVN KSDNIKNDPM KRPYVIKPLT QGSSIGVEVI FAEDDFNFAD YDFPYGDQVI IEQYIKGR-- ELQVAVLNGK ALGALEIKLL KN-RFYDYET KYTAGFADHL
RicConM7   INMAEGIVVN KSDNIKNDPM KRPYVIKPLT QGSSIGVEVI FAEDDFNFAD YDFPYGDQVI IEQYIKGR-- ELQVAVLNGK ALGALEIKLL KN-RFYDYET KYTEGFADHL
RicJapYH   INMAESIVVN KSDNIKNDPM KRPYVIKPLT QGSSIGVEVI FAEDDFNFAD YDFPYGDQVI IEQYIKGR-- ELQVAVLNGK ALGALEIKLL KN-RFYDYET KYTEGFADHL
RicSloDC   INMAESIVVN KSDNIKNDPM KRPYVIKPLT QGSSIGVEVI FAEDDFNFAD YDFPYGDQVI IEQYIKGR-- ELQVVVLNGK ALGALEIKLL KN-RFYDYET KYTEGFADHL
RicParPo   INMAESIVVN KSDNIKNDPM KRPYVIKPLT QGSSIGVEVI FAEDDFNFAD YDFPYGDQVI IEQYIKGR-- ELQVAVLNGK ALGALEIKLL KN-RFYDYET KYTEGFADHL
RicRhipi   INMAESIVVN KSDNIKNDPM KRPYVIKPLT QGSSIGVEVI FAEDDFNFAD YDFPYGDQVI IEQYIKGR-- ELQVAVLNGK ALGVLEIKLL KN-RFYDYET KYTEGFADHL
RicHeilo   INMAESIVVN KSDNIKNDPM KRPYVIKPLT QGSSIGVEVI FAEDDFNFAD YDFPYGDQVI IEQYIKGR-- ELQVAVLNGK ALGALEIKLL KN-RFYDYET KYTEGFADHL
RicAmbly   INMAESIVVN KSDNIKNDPM KRPYVIKPIT QGSSIGVEVI FAEDDFNFAD YDFPYGDQVI IEQYIKGR-- ELQVAVLNGK ALGVLEIKLL KN-RFYDYET KYTEGFADHL
RicPhili   INMAESIVVN KSDNIKNDPM KRPYVIKPLT QGSSIGVEVI FAEDDFNFAD YDFPYGDQVI IEQYIKGR-- ELQVAVLNGK ALGALEIKLL KN-RFYDYET KYTAGFADHL
RicAfrE5   INMAESIVVN KSDNIKNDPM KRPYVIKPLT QGSSIGVEVI FAEDDFNFAD YDFPYGDQVI IEQYIKGR-- ELQVAVLNGK ALGALEIKLL KN-RFYDYET KYTEGFADHL
RicMonta   INMAESIVVN KSDNIKNDPM KRPYVIKPLA QGSSIGVEVI FAEDDFNFAD YDFPYGDQVI IEQYIKGR-- ELQVVVLNGK ALGALEIKLL KN-RFYDYET KYTEGFADHL
RicPeacR   INMAESIVVN KSDNIKNDPM KRPYVIKPLA QGSSIGVEVI FAEDDFNFAD YDFPYGDQVI IEQYIKGQGR ELQVAVLNGK ALGALEIKLL KN-RFYDYET KYTEGFADHL
RicTypWi   INMADSIVVS KSDHINTDPM KRPYVIKPLK QGSSIGVEVI FEEDDFHFID YDFPYGEDII IEQYIQGQ-- ELQVALLNGK ALGVLEIKLL KN-RFYDYET KYNKGFAKHV
RicProME   INIADSIVVS KSDHINTDPM KRPYVIKPLK QGSSIGVEVI FEEDDFNFTD YDFPYGEDII IEQYIQGQ-- ELQVALLNGK ALGVLEIKLL KN-RFYDYET KYNEGFAEHV
RicAuC_c   INTAESIVVS KSDNIKTDPM KRPYVIKPLT QGSSIGVEVI FEEDDFNFAD YDFPYGDQVV IERYIKGR-- ELQVAVLNSK ALGVLEIKLL KN-RFYDYAT KYTEGFAEHL
RicAkarH   INTAESIVVS KSYNINTDPM KRPYVIKPLT QGSSIGVEVI FEEDDFNFAD YDFPYGDQVV IERYIKGR-- EFQVAVLNSK ALGALEIKLI KN-RFYDYET KYTEGFAEHL
RicFel_c   INMAESIIVN KSDNIKSDPV KRPYVIKPLT QGSSIGVEVI FEEDDFNFAD YNFPYGYQVI IEQYIKGR-- ELQVAVLNGK ALGALEIKLL KN-RFYDYET KYTEGFAEHL
RicCaMcK   INMAESIVVN KSDNIKIEPM KRPYVIKPIT QGSSIGIEVI FEEDDFNFAN YDFPYGDQVI IEKYIKGR-- ELQVAVLNGK ALGVLEIKLL KN-RFYDYET KYTEGFAEHL
RicCa410   INMAESIVVN KSDNIKIEPM KRPYVIKPIT QGSSIGIEVI FEEDDFNFAN YDFPYGDQVI IEKYIKGR-- ELQVAVLNGK ALGVLEIKLL KN-RFYDYET KYTEGFAEHL
RicBO389   INMAESIVIS KGDNIKTDPI KRPYVIKPFT QGSSIGVEVI FEEDDFNFAN YDFPYGDEVI IEKYIKGR-- ELQVAILNGK ALGALEIKLL KN-RFYDYET KYTEGFAEHL
RicBR3_c   INMAESIVIS KGDNIKTDPI KRPYVIKPFT QGSSIGVEVI FEEDDFNFAN YDFPYGDEVI IEKYIKGR-- ELQVAILNGK ALGALEIKLL KN-RFYDYET KYTEGFAEHL
OriTsuBo   IKTPASITIN SNDNVINDPI LRPYVIKPLQ QGSSIGVKIV HLEDKFLFKD YKFQFGNKVL VEQYIKGR-- ELQVALLDGK VVGILEIKML KDKIFYDYQS KYSPGFAEHI
OriTsuIk   IKTPASITIN SNDNVIDDPI LRPYVIKPLQ QGSSIGVKIV HLEDKFLFKD YKFQFGNKVL VEQYIKGR-- ELQVALLDGQ VVGILEIKML KDKIFYDYQS KYSPGFAEHI
NIES_425   INIPKSKLIK KSDGYTKDPI KRPYVIKPLS QGSSVGVQVI FPEDIFSFGD YDFPYGDEIL VEEYIKGR-- EMQVAVLNGR AIGILEIKLL KNKRFYDYET KYTEGFSEHL
UTEX2180   IKIPESKLIK KSDGYTKDPI KRPYVIKPLA QGSSVGVQVI FPDDIFSFGN YDFPYGDEIL VEEYIKGR-- EMQVAVLNGR AIGVLEIKLL KNKRFYDYET KYTEGFAEHL
NIES_577   IKIPESKLIK KSDGYTKDPI KRPYVIKPLS QGSSVGVQVV FPEDIFSFSD YAFPYGDEIL IEEYIKGR-- EMQVAVLNGR AIGVLEIKLL KNKRFYDYET KYTEGFAEHL
EVE_____   IKIPESKLIK KSDGYTKDPI KRPYVIKPLS QGSSVGVQVV FPEDIFSFSD YDFPYGDEIL IEEYIKGR-- EMQVAVLNGR AIGVLEIKLL KNKRFYDYET KYTEGFAEHL
NIES_866   IKIPESKLIK KSDGYTKDPI KRPYVIKPLS QGSSVGVQVV FPEDIFSFSD YDFPYGDEIL IEEYIKGR-- EMQVAVLNGR AIGVLEIKLL KNKRFYDYET KYTEGFAEHL
```

```
       551
RicMasM5  CPAPLPANLY EKLLIESEKI YKTMNC-KGP ARAEFILEEQ TNKLYALELN THPGMTPLSI VPEIAAYAGI NFTNLIEEII KTASFES
RicMaA_c  CPAPLPANLY EKLLIESEKI YKTMNC-KGP ARAEFILEEQ TNKLYALELN THPGMTPLSI VPEIAAYAGI NFTNLIEEII KTASFES
RicRicIo  CPAPLPANLY EKLLIESEKI YKTMNC-KGP ARAEFILEEQ TNKLYALEIN THPGMMSLSI VPEIAAYAGI NFTNLIEEII KTASFES
RicConM7  CPAPLPANLY EKLLIESEKI YKTMNC-KGP ARAEFILEEQ TNKLYALEIN THPGMMPLSI VPEIAAYAGI NFTNLIEEII KTASFES
RicJapYH  CPVPLPANLY EKLLIESEKI YKTMNC-KGP ARAEFILEEQ TNKLYALEIN THPGMTPLSI VPEIAAYAGI NFTNLIEEII KTASFES
RicSloDC  CPAPLPANLY EKLLIESEKI YKTMNC-KGP ARAEFILEEQ TNKLYALEIN THPGMMPLSI VPEIAAYAGI NFTNLIEEII KTASFES
RicParPo  CPAPLPANLY EKLLIESEKI YKTMNC-KGP ARAEFILEEQ TNKLYALEIN THPGMMSLSI VPEIAAYAGI NFTNLIEEII KTASFES
RicRhipi  CPAPLPANLY EKLLIESEKI YKTMNC-KGP ARAEFILEEQ TNKLYVLELN THPGMTPLSI VPEIAAYAGI NFTNLIEEII KTASFES
RicHeilo  CPVPLPANLY EKLLIESEKI YKTMNC-KGP ARAEFILEEQ TNKLYALEIN THPGMTPLSI VPEIAAYAGI NFTNLIEEII KTASFES
RicAmbly  CPAPLPANLY EKLLIESEKI YKTINC-KGP ARAEFILEEQ TNKLYALEIN THPGMTPLSI VPEIAAYVGI NFTNLIEEII KTASFES
RicPhili  CPAPLPANLY EKLLIESEKI YKTMNC-KGP ARAEFILEEQ TNKLYALEIN THPGMMSLSI VPEIAAYAGI NFTNLIEEII KTASFES
RicAfrE5  CPAPLPANLY EKLLIESEKI YKTMNC-KGP ARAEFILEEQ TNKLYALEIN THPGMTPLSI VPEIAAYAGI NFTNLIEEII KMASFES
RicMonta  CPAPLPANLH EKLLIESEKI YKTMNC-KGP ARAEFILEEQ TNKLYALEIN THPGMTPLSI VPEIAAYAGI NFTNLIEEII KTASFES
RicPeacR  CPAPLPANLY EKLLIESEKI YKTMNC-KGP ARAEFILEEQ TNKLYALEIN THPGMMPLSI VPEIAAYAGI NFTNLIEEII KTASFES
RicTypWi  CPAQLPANLY KKLLIESEKI YKTINC-KGP VRAEFILEEQ TNKLYVLEIN THPGMTPLSI VPEIAAYAGI SFTNLIEEII KMASFES
RicProME  CPAPLPANLY KKLLIESEKI YKTINC-KGP VRAEFILEEQ TNKLYVLEIN THPGMTPLSI VPEIAAYAGI NFTNLIEEII KMASFES
RicAuC_c  CPAPLPANLY EKLLIASEKI YKTMNC-KGP ARAEFILEEQ TNKLYALEIN THPGMTLLSI VPEIAAYAGI NFTNLIEEII KTASFES
RicAkarH  CPAPLHANLY EKLLIESEKI YKTMNC-KGP ARAEFILEEQ TNKLYALEIN THPGMTPLSI VPEIAAYAGI NFTNLIAEII KTASFES
RicFel_c  CPAPLPTNLY EKLLVESEKI YKTMNC-KGP ARAEFILEEQ TNKLYALEIN THPGMTPLSI VPEIAAYAGI NFTNLIEEII KTASFES
RicCaMcK  CPAPIPTNLY DKLLIESEKI YKTMNC-KGP ARVEFLLEDQ TNKLYALEIN THPGMTPLSI VPEIAAYAGI NFTNLIEEII KAASFES
RicCa410  CPAPIPTNLY HKLLIESEKI YKTMNC-KGP ARVEFLLEDQ TNKLYALEIN THPGMTPLSI VPEIAAYAGI NFTNLIEEII KAASFES
RicBO389  CPAPLPTDIY DKLLKESEKI YNTMNC-KGA ARAEFILEDG TNKLYALEIN THPGMTPLSI VPEIAAYHGI DFVNLIEEIL KTASFES
RicBR3_c  CPAPLPTDIY DKLLKESEKI YNTMNC-KGA ARVEFILEDG TNKLYALEIN THPGMTPLSI VPEIAAYHGI DFVNLIEEIL KTASFES
OriTsuBo  IPPRLPVNTV NQIMNIAEKV YKIFDC-RGP CRLECILSDI DNEVYVIELN THPGMTSLSS YPEIAQYYGI SFNELVERIL ITAKCD-
OriTsuIk  IPPMLPVNTV NQVINIAEKV YKIFDC-RGP CRLECILSDI DNEVYVIELN THPGMTSLSS YPEIAQYYGI SFNELVERIL ITAKCD-
NIES_425  LPAPVSSEIY NNMKAMAEKA CIALDCVTGM IRVEMIYSSE KNALYMLEVN THPGMTPLSI CPEIATLENM SYKDLVKEIL EEARFE-
UTEX2180  LPAPVSSEMY NNIKDLAEKA CSVLDCVTGM IRVEMIYSPE RNALYMLEVN THPGMTPLSI CPEIATLENI SYKDLVKEIL EEAKFE-
NIES_577  LPAPVSLEMY NNIKALAEKA CSVLDCVTGM IRVEMIYSSE KNELYMLEVN THPGMTQLSI CPEIATLENM SYKDLVKEIL EEAKFE-
EVE_____  LPAPVSLEMY NNIKALAEKA CSVLDCVTGM IRVEMIYSSE KNELYMLEVN THPGMTPLSI CPEIATLENM SYKDLVKEIL EEAKFE-
NIES_866  LPAPVSLEMY NNIKALAEKA CSVLDCVTGM IRVEMICSSE KNELYMLEVN THPGMTPLSI CPEIATLENM SYKGLVKEIL EEAKFE-
```

Internal transcribed spacer 2 region (ITS-2)

```
          1
UTEX1865  GGTTAATACT CAATGCAATG CACAT----G CAT----GTG TGTACTGCAT TGGATCTGGC TGTCTCGGTG CTTA------ ---------- ---------- ----CTAC--
EVE______ GGTTAAATCT CAATGCCGTA CACACGCATG ATGATGCATG TGTACGACAT TGGATCTTGT TTTCTCGGCA TTAA---ATC ATTT------ ---------- ----ACTCA-
UTEX1886  GGTTAAATCT CAATGYCGTA CACACGCATG ATGATGCATG TGTACGACAT TGGATCTTGT TTTCTCGGCA TTAA---ATC ATTT------ ---------- ----ACTCA-
NIES_398  GGTTAAATCT CAATGTCGTA CACACGCATG ATGATGCATG TGTACGACAT TGGATCTTGT TTTCTCGGCA TTAA---ATC ATTT------ ---------- ----ACTCA-
UTEX2903  GGTTAAATCT CAATGCCGTA CACACGCATG ATGATGCACG TGTACGACAT TGGATCTTGT TTTCTCGGCA TTAA---ATC ATTT------ ---------- ----ACTCA-
NIES_732  GGTTAAAACT CAGTGCCGTA CCCGCATATG TGTGTGTGTG TGTACGACAT TGGATCTGGT TTTCTCGGCA GTAA---A-C TTTT------ ---------- ----ATATA-
NIES_866  GGTTAAAGCT CATCGCCGTA CATGC----A CAT----GTG TGTACGGCGT TGGACCTGGT TTTCTCGGCA CATACAGATC TTTTAT--AT GTT------- ----TAATA-
UTEX2170  GGTTAAAACT CAGCGCCGTA CATAC----A AAT----GTG TGTACGACTT TGGATCTGGT TTTCTCGGCA CATGCAAATC GTTTGT--AT GTTC------ ----AATAA-
UTEX1874  GGTTAAATCT CAGTGCCGTA CATGC----A TCT----GTG TGTACGGCAT TGGATCTGGT TTCCTCGGCA CATACAAATC CTTTGTTTAT GTTTGAATTA TAGTAATAAT
UTEX2180  GGTTAAATCT CAGTGCCGTA CATGC----A TCT----GTG TGTACGGCAT TGGATCTGGT TTCCTCGGCA CATACAAATC CTTTGTTTAT GTTTGAATTA TAGTAATAAT

        111
UTEX1865  ---------- -----TAA-- ---------- TGCCGGGTCA GCTGAAGTAT GCAGATGCTG AACCATGGAC CGTT---CAT --------CG GGCCTCTACT GGGTAGGCAT
EVE______ ---------A A-TCGTTTAA ---------- TGCCGGGTGA ATTGAAGTAT GTAGATGCTT AACCGTGGAC CGTT--TCAT --------CG GGCCTTTACT GGGTAGGCAA
UTEX1886  ---------A A-TCGTTTAA ---------- TGCCGGGTGA ATTGAAGTAT GTAGATGCTT AACCGTGGAC CGTT--TCAT --------CG GGCCTTTACT GGGTAGGCAA
NIES_398  ---------A A-TCGTTTAA ---------- TGCCGGGTGA ATTGAAGTAT GTAGATGCTT AACCGTGGAC CGTT--TCAT --------CG GGCCTTTACT GGGTAGGCAA
UTEX2903  ---------A A-TCGTTTAA ---------- TGCCGGGTGA ATTGAAGTAT GTAGATGCTT AACCGTGGAC CGTT--TCAT --------CG GGCCTTTACT GGGTAGGCAA
NIES_732  ---------A AAT-GTTTAA -TG------- TGCCGGGTGG ACTGAAGTGT GTAGATGCTG AACCATGGAC CGTATTATCT --------CG GGCCTCTACT GGGTAGGCAA
NIES_866  ---------A TAACGTATAA CTG--AGGGG TGCCGGGTGA ACTGAAGTAT GCAGATGCTG GACCGTGGAC CATATACTAT --------CG GGCCTCTACT GGGTAGGCAA
UTEX2170  ---------- TGACGTACAA CCG--AGGGG TGCCGGGTGA ACTGAAGTAT GCAGATGCTG GACCGCGGAC CATA---CAT -----TATCG GGCCTCTACT GGGTAGGCAA
UTEX1874  AATAATTCGA ACACCTAACT CTGTGAGATA TGCCGGGTGA ACCGAAGTAT ACAGATGCTG GATCGTGGAC CGTTGATATT TTATATATCG GGCCTCTACT GGGTAGGCAA
UTEX2180  AATAATTCGA ACACCTAACT CTGTGAGATA TGCCGGGTGA ACCGAAGTAT ACAGATGCTG GATCGTGGAC CGTTGATATT TTATATATCG GGCCTCTACT GGGTAGGCAA

        221
UTEX1865  AACACACCTT T--------- ---------- ---------- ------GGTT GTTAGCTGCA A--TGCTTTA GTAGATGGCT T-CGGGTACT GTGTTTGGCA -CAAAGCAGG
EVE______ AGTCAGCGTT ATG------- ---------- -------TAA TGCATACGTT GCTTGCTGCA A--TGCTTTA GTAGATGGCT TTGAGGTTCT GTGTTTCGCA AAAAATCAGG
UTEX1886  AGTCAGCGTT ATG------- ---------- -------TAA TGCATACGTT GCTTGCTGCA A--TGCTTTA GTAGATGGCT TTGAGGTTCT GTGTTTCGCA AAAAATCAGG
NIES_398  AGTCAGCGTT ATG------- ---------- -------TAA TGCATACGTT GCTTGCTGCA A--TGCTTTA GTAGATGGCT TTGAGGTTCT GTGTTTCGCA AAAAATCAGG
UTEX2903  AGTCAGCGTT ATG------- ---------- -------TAA TGTATACGTT GCTTGCTGCA A--TGCTTTA GTAGATGGCT TTGAGGTTCT GTGTTTCGCA AAAAATCAGG
NIES_732  AGTCAACCTT A--------- ---------- ---------- --AGTGCGTT GTTTGCTGCA T--TGCTTTA GTAGATGGCT TTGAGGTCCT GTGCTTGGCA AAAAAACAGG
NIES_866  AATCAACCTT TGT------- ---------- --------TA CTACTGTGTT GTTTGCTGTG A--TGCTTTA GTAGATGGCT TTGGGGTCCT GTGTTTGGCA AAAAAGCAGG
UTEX2170  AATCAACCTT TGT------- ---------- --------TA TCACTGTGTT GTTTGCTGCA A--TGCTTTA GTAGATGGCT TTGGGGTCCT GTGTTTGGCA AAAAAGCAGG
UTEX1874  AATCAACCTG TAGCAGTGGT CTATACTATA CTATGCTCTA CTACTGTGTT GTTTGCTGCA AAATGCTTTA GTAGATGGCT TTGGGGTCCT GTGTTTGGCA AAAAAGCAGG
UTEX2180  AATCAACCTG TAGCAGTGGT CTATACTATA CTATGCTCTA CTACTGTGTT GTTTGCTGCA AAATGCTTTA GTAGATGGCT TTGGGGTCCT GTGTTTGGCA AAAAAGCAGG

        331
UTEX1865  AGTACTGC-- ---------- ---------- ---------- ---------- --------TT CAT------- ---------- ---------- ---------- ----------
EVE______ A-T-TACTGT TGTACTGCAC GGGACT---- ---------- ------CTGT ---------- ---------- AGTAC----- ---TGTG-TA ---------- ------GTAC
UTEX1886  A-T-TACTGT TGTACTGCAC GGGACT---- ---------- ------CTGT ---------- ---------- AGTAC----- ---TGTG-TA ---------- ------GTAC
NIES_398  A-T-TACTGT TGTACTGCAC GGGACT---- ---------- ------CTGT ---------- ---------- AGTAC----- ---TGTG-TA ---------- ------GTAC
UTEX2903  A-T-TACTGT TGTACTGCAC AGGACT---- ---------- ------CTGT ---------- ---------- AGTAC----- ---TGTG-TA ---------- ------GTAC
NIES_732  A-T------- ---------- ---------- ---------- ---------- -----ATATT TTA------A TCTAAACAAC A--------- ---------- ----------
NIES_866  AGTATGCGAC TCTGC-ACAC GCGTGTGCT- ---------- --------GT ACT-----TT ------GTGC AGTACAAACC ATATGTG-TA TGTGT-TGTT G------TAC
UTEX2170  AGTATGCGAC TCTGC-ACAT GTGTGTGCT- ---------- --------GT ACT-----TT ------GTGC AGTACACATG GCATGTG-TA CGTGT-TGTT G------TAC
UTEX1874  AGTGTACGTG GCTACAATAC GTATCTGCTC TGCACTTGAG TGTGTGCTGT ACTACAACTT AAACGTGTGC AGTACACACC ATGTGTGGTG TGCATGTGTT GTCGTCGTAC
UTEX2180  AGTGTACGTG GCTACAATAC GTATCTGCTC TGCACTTGAG TGTGTGCTGT ACTACAACTT AAACGTGTGC AGTACACACC ATGTGTGGTG TGCATGTGTT GTCGTCGTAC
```

```
           441
UTEX1865  -----GCAGT ACAACCCCCC CATCATTTTC T
EVE______ A----GCAAG TG-----TAC TATTTTTTTC T
UTEX1886  A----GCAAG TG-----TAC TATTTTTTTC T
NIES_398  A----GCAAG TG-----TAC TATTTTTTTC T
UTEX2903  A----GCAAG TG-----TAC TATTTTTTTC T
NIES_732  ---------- ---------- ---TTTTTTC T
NIES_866  ---------- -------TTT TTATTTTTCC T
UTEX2170  ---------- ------TTTA TATTTTTTTC T
UTEX1874  ATCATTATAT ATA--TATAT AATTATTTTC T
UTEX2180  ATCATTATAT ATATATATAT AATTATTTTC T
```
