# Supplementary material for: Two Different Rickettsial Bacteria Invading Volvox carteri
Source: PLoS One. 2015 Feb 11;10(2):e0116192. doi: 10.1371/journal.pone.0116192 (PMC4324946; doi:10.1371/journal.pone.0116192)

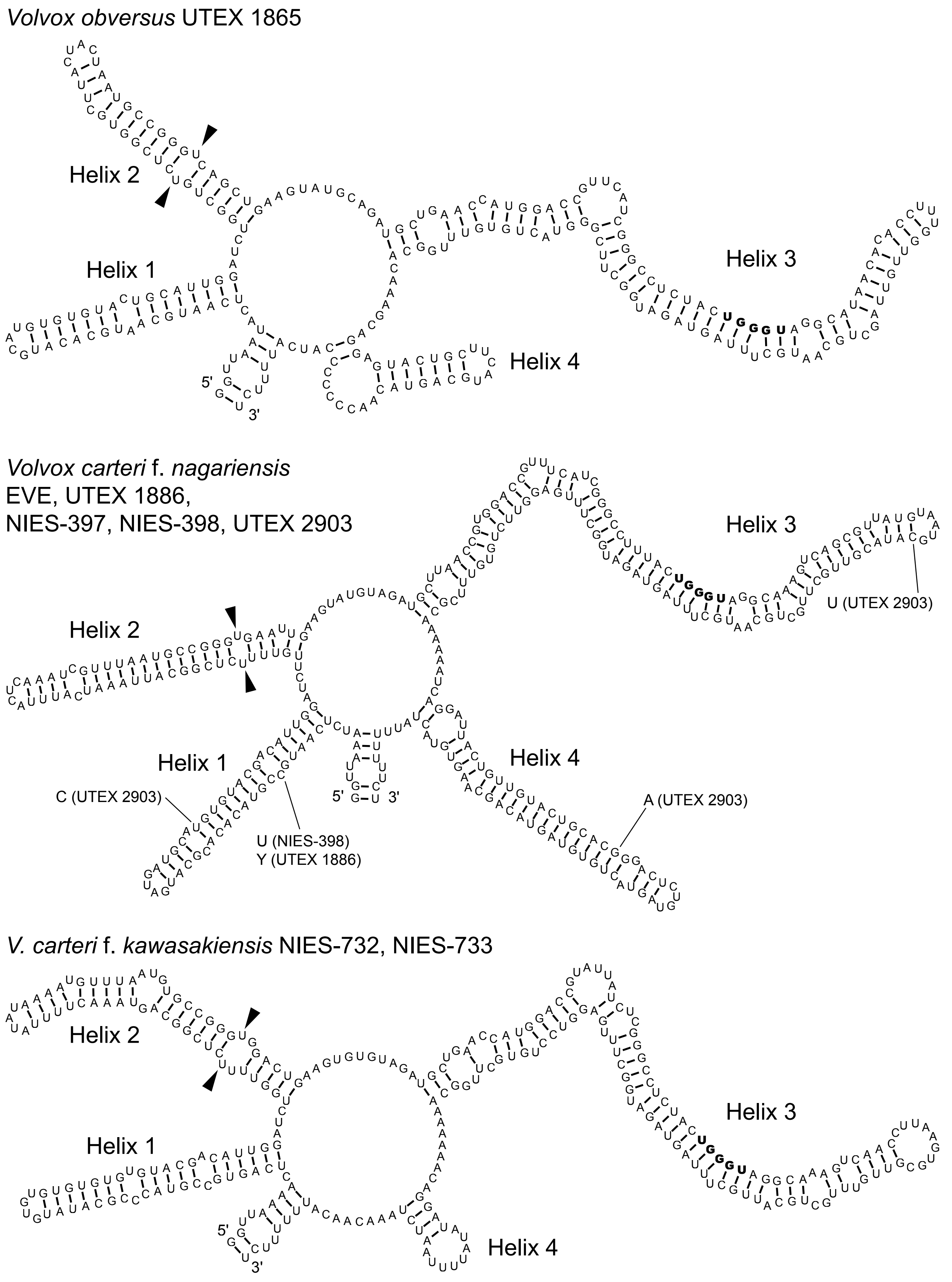

*Volvox obversus* UTEX 1865
Helix 2
Helix 1
Helix 3
Helix 4
5'
3'
*Volvox carteri* f. *nagariensis*
EVE, UTEX 1886,
NIES-397, NIES-398, UTEX 2903
Helix 3
U (UTEX 2903)
Helix 2
Helix 1
Helix 4
C (UTEX 2903)
A (UTEX 2903)
U (NIES-398)
Y (UTEX 1886)
5'
3'
*V. carteri* f. *kawasakiensis* NIES-732, NIES-733
Helix 2
Helix 1
Helix 3
Helix 4
5'
3'

*Volvox carteri* f. *weismannia*
UTEX 1875, UTEX 1876

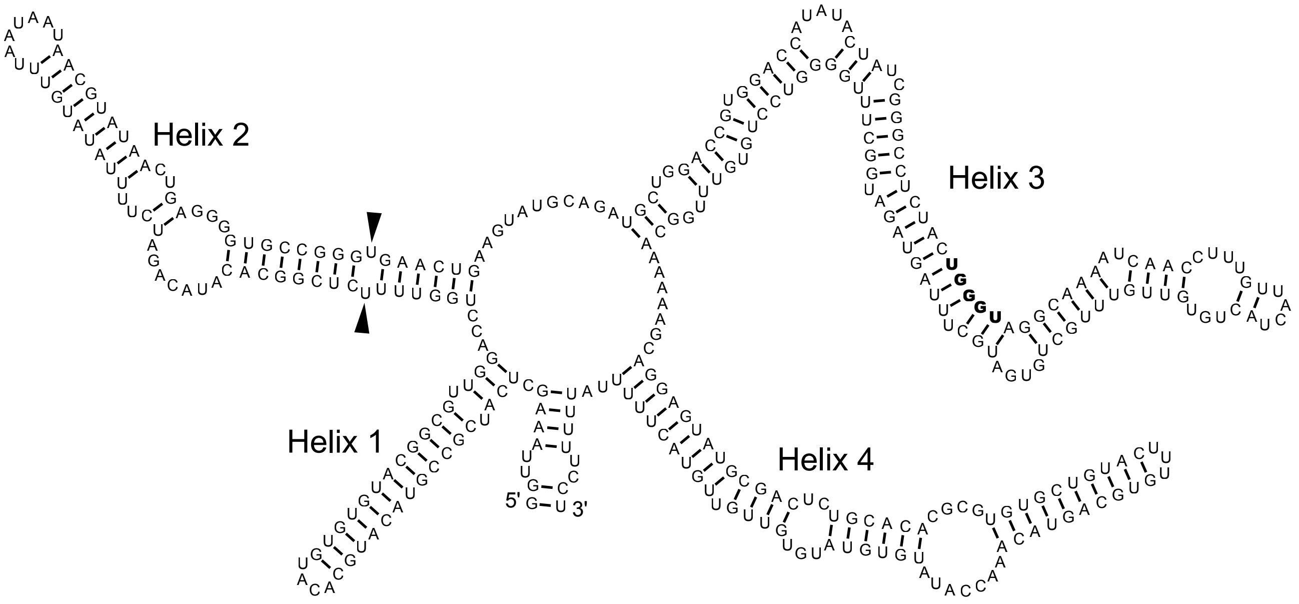


*Volvox carteri* f. *weismannia*
UTEX 2170

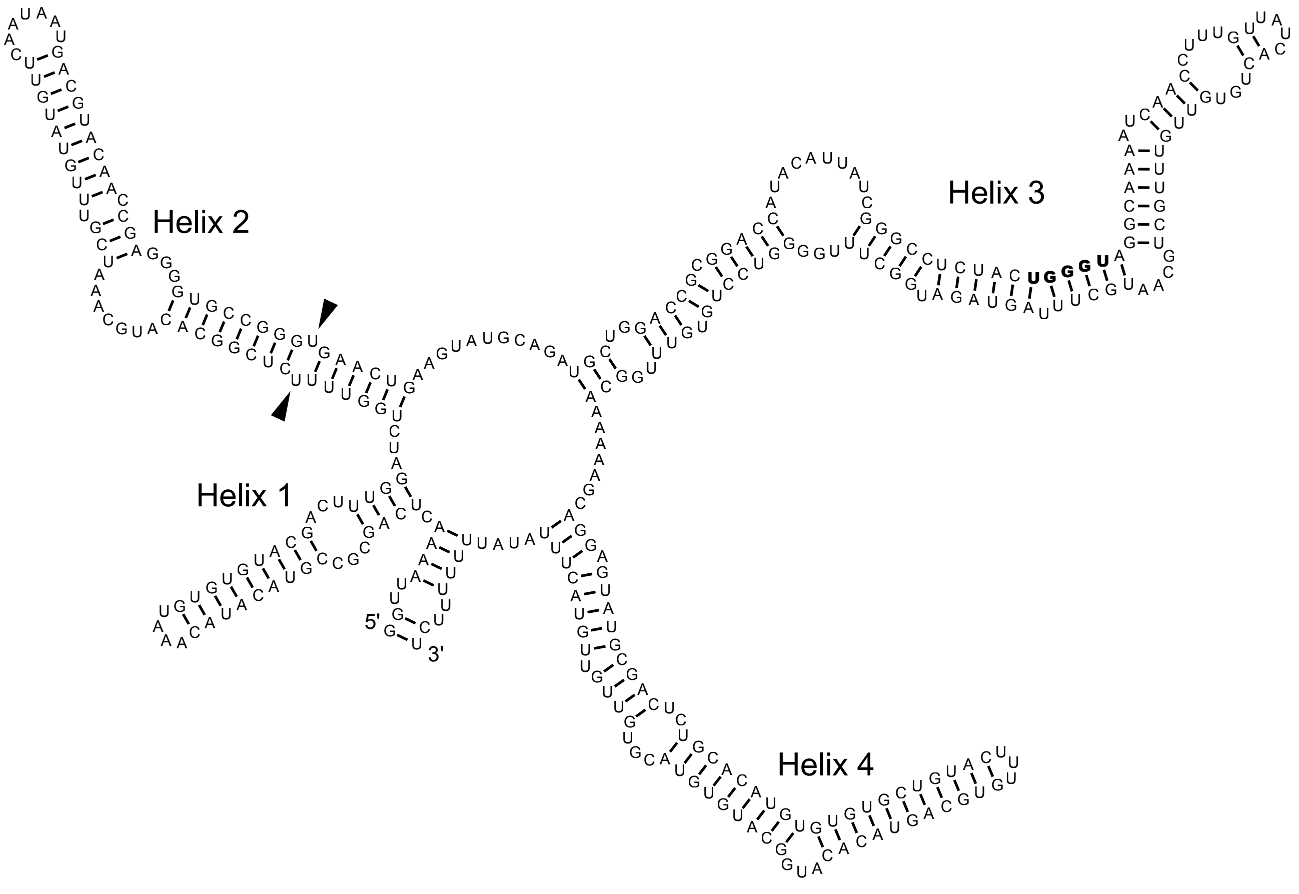

*Volvox carteri* f. *weismannia*
UTEX 2180, UTEX 1874, UTEX 2904

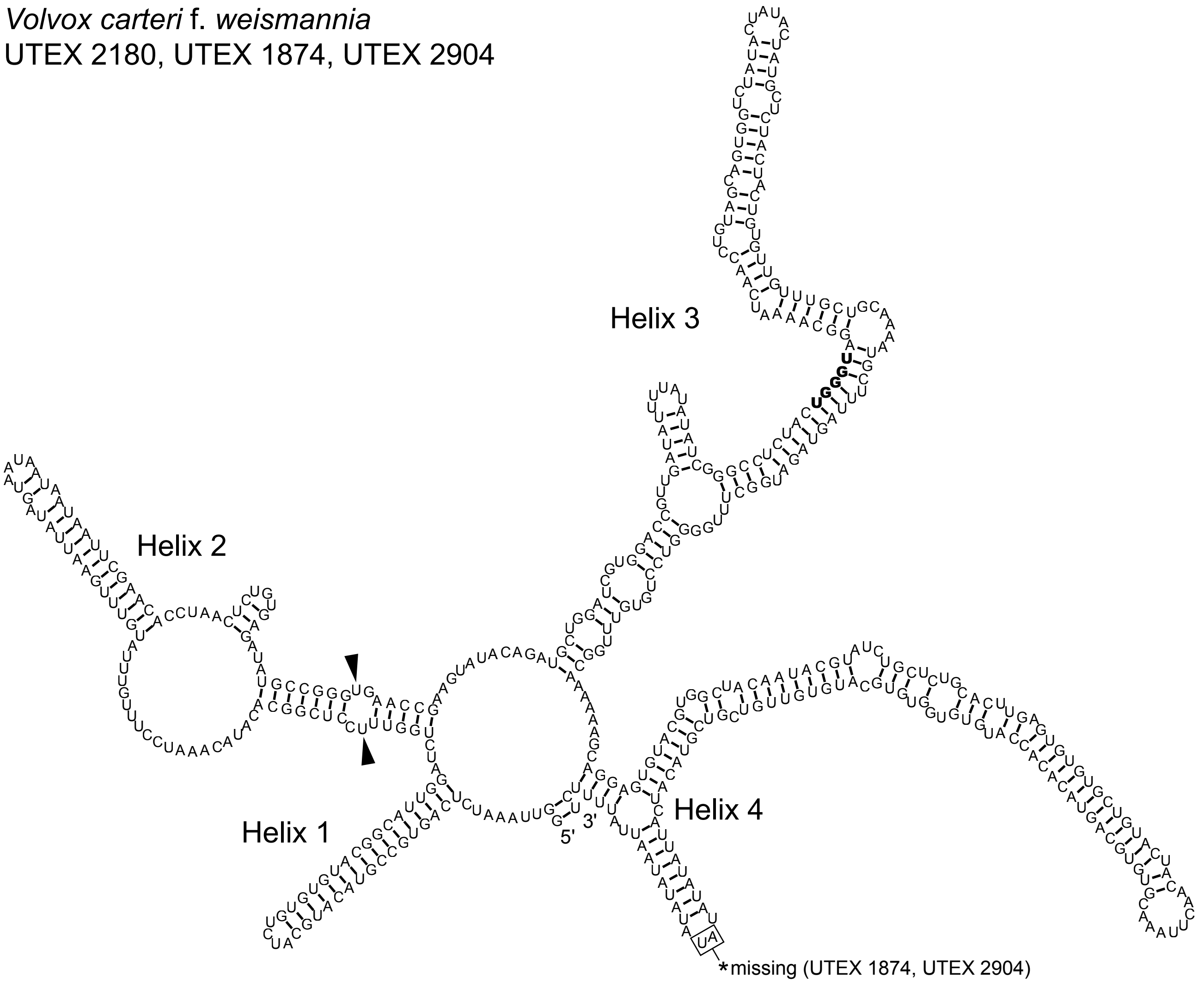

Supplement: S2 File — The structure was predicted and drawn as described in Materials and Methods. The U-U mismatch in helix 2 (arrowheads) and the UGGU motif on the 5′ side near the apex of helix 3 (boldface) are the universally conserved features [43]. Among the ITS-2 sequences of V. carteri f. nagariensis strains, one nucleotide of UTEX 1886 and NIES-398, and three of UTEX 2903 are different from those of strains EVE and NIES-397 (shown around the structures). ITS-2 sequences of V. carteri f. weismannia strains UTEX 1874 and UTEX 2904 differ from that of V. carteri f. weismannia strain UTEX 2180 in missing of one pair of “AU” from AU repeats in helix 4 (asterisk). (PDF) [file pone.0116192.s007.pdf]
