# Supplementary material for: Two Different Rickettsial Bacteria Invading Volvox carteri
Source: PLoS One. 2015 Feb 11;10(2):e0116192. doi: 10.1371/journal.pone.0116192 (PMC4324946; doi:10.1371/journal.pone.0116192)
Supplement: S1 Table — BLASTN searches were performed on Phytozome v9.1, using 81 contigs (>5 kb) from preliminary genome assembly database of C. cerasiformis NIES-425 rickettsial endosymbiont. Hits with the E-value ≤1.0e-50 of BLASTN searches and the information of ftsQ-like sequence found in following BLASTN search in scaffold 6 of EVE genome are shown. (DOC) [file pone.0116192.s008.doc]

**Table S1. The high scoring hits of *Volvox carteri* f. *nagariensis* strain EVE genome sequence by BLASTN searches using *Carteria* *cerasiformis* rickettsial endosymbiont draft genome.** BLASTN searches were performed on Phytozome v9.1, using 81 contigs (>5 kb) from preliminary genome assembly database of *C. cerasiformis* NIES-425 rickettsial endosymbiont. Hits with the E-value ≤1.0e-50 of BLASTN searches and the information of *ftsQ*-like sequence found in following BLASTN search in scaffold 6 of EVE genome are shown.

| Scaffold | Position |  | Score | E-value | Identity (%) | (bp/bp) | Annotation |
| --- | --- | --- | --- | --- | --- | --- | --- |
| 6 | 940432 | 939419 | 1373.7 | 0 | 90.0 | 913/1014 | D-alanyl-alanine ligase [*Rickettsia japonica*] |
| 6 | 938261 | 937504 | 1014.8 | 0 | 89.6 | 682/761 | UDP-N-acetylenolpyruvoylglucosamine reductase [*Rickettsia helvetica*] |
| 6 | 937505 | 937077 | 747.9 | 0 | 98.6 | 423/429 | *Rickettsiaceae* endosymbiont of *Pleodorina japonica* gene for 16S ribosomal RNA |
| 36 | 56105 | 56609 | 446.7 | 5.90E-121 | 78.9 | 426/540 | Hypothetical protein [*Rickettsia rhipicephali*] |
| 30 | 1311260 | 1311562 | 380 | 7.20E-101 | 87.8 | 266/303 | Putative bifunctional glutamate synthase subunit beta/2-polyprenylphenol hydroxylase [*Rickettsia* *canadensis*] |
| 1 | 13232743 | 13232265 | 352.9 | 1.00E-92 | 76.2 | 374/491 | Surface antigen (D15) [*Desulfohalobium* *retbaense*] |
| 60 | 166248 | 166583 | 336.7 | 7.70E-88 | 82.4 | 277/336 | DNA repair protein RadC [*Rickettsia massiliae*] |
| 1 | 8394848 | 8394414 | 333.1 | 9.30E-87 | 76.8 | 350/456 | Heat shock protein GrpE [*Amorphus coralli*] |
| 114 | 9149 | 9491 | 324.1 | 4.80E-84 | 81.0 | 278/343 | Hypothetical protein [*Rickettsiaceae* bacterium Os18] |
| 7 | 3741662 | 3741429 | 309.7 | 1.10E-79 | 89.3 | 209/234 | MFS transporter [*Rickettsia bellii*] |
| 11 | 2289762 | 2289993 | 300.6 | 5.50E-77 | 84.1 | 238/283 | RND efflux transporter [*Pseudomonas aeruginosa* PA99] |
| 24 | 1251646 | 1251958 | 298.8 | 1.90E-76 | 82.5 | 259/314 | Small multidrug resistance (SMR) family efflux pump [*Rhizobium tropici*] |
| 6 | 2460215 | 2459921 | 255.6 | 2.10E-63 | 79.2 | 236/298 | Hypothetical protein GUITHDRAFT_133732 [*Guillardia theta* CCMP2712] |
| 6 | 2082447 | 2082244 | 252 | 2.50E-62 | 87.9 | 181/206 | Dihydrodipicolinate reductase [*Candidatus* Endolissoclinum faulkneri] |
| 13 | 350503 | 350293 | 241.1 | 4.50E-59 | 85.8 | 182/212 | No significant hits |
| 17 | 2041301 | 2041462 | 232.1 | 2.40E-56 | 92.0 | 150/163 | UDP-3-O-(3-hydroxymyristoyl) glucosamine N-acyltransferase (LpxD) [*Rickettsia typhi*] |
| 6 | 2459685 | 2459520 | 219.5 | 1.50E-52 | 89.2 | 148/166 | Recombination protein RecJ [*Rickettsia bellii*] |
| 11 | 57355 | 57566 | 217.7 | 5.20E-52 | 83.0 | 176/212 | Phenylalanyl-tRNA synthetase subunit beta [*Rickettsia bellii*] |
| 48 | 507707 | 507884 | 217.7 | 5.20E-52 | 87.1 | 155/178 | Elongation factor Tu family protein [*Wolbachia* endosymbiont of *Drosophila ananassae*] |
| 6^a^ | 940359 | 940432 | 93.3 | 8.40E-18 | 87.8 | 65/74 | Cell division protein *ftsQ* [*Rickettsia* *canadensis*] |

^a^Hit found in additional search (see Materials and Methods).
