# Supplementary material for: Two Different Rickettsial Bacteria Invading Volvox carteri
Source: PLoS One. 2015 Feb 11;10(2):e0116192. doi: 10.1371/journal.pone.0116192 (PMC4324946; doi:10.1371/journal.pone.0116192)
Supplement: S2 Table — (DOC) [file pone.0116192.s009.doc]

**Table S2.** The list of primers used in genomic PCR, semi-quantitative genomic PCR and sequencing of this study.

| Primer designation | Primer sequence (5' to 3') | Target | Forward/ Reverse |
| --- | --- | --- | --- |
| 9F [1] | GAGTTTGATCCTGGCTCAG | Universal 16S rRNA gene | F |
| 1492R [1] | GCTTACCTTGTTACGACTT | Universal 16S rRNA gene | R |
| enRB [2] | TAGCTCACCAGCTTCGGGTAAA | Rickettsial 16S rRNA gene | R |
| enFE [2] | CGCGTAGGCGGATTAGTAAGTTGG | Rickettsial 16S rRNA gene | F |
| enRG [2] | CGTCTTGCTTCCCTCTGTAAGT | Rickettsial 16S rRNA gene | R |
| 16S-mkFJ | TGACATGGTGATCGTAGGTTACAG | Rickettsial 16S rRNA gene | F |
| 16S-enFK | ACTTACAGAGGGAAGCAAGACG | Rickettsial 16S rRNA gene | F |
| 16S-enRL | CACTAAAATTGGAGCAAGCCCC | Rickettsial 16S rRNA gene | R |
| 16S-enRM | AGCGATAAATCTTTCCTCCTTGG | Rickettsial 16S rRNA gene | R |
| 16S-enFN | GCTTAACCTCGGAACTGCTTTC | Rickettsial 16S rRNA gene | F |
| 18S-FA [3] | AACCTGGTTGATCCTGCCAGT | Chlorophytes 18S rRNA gene | F |
| 18S-RD [3] | GCTGGCACCAGACTTGCCCTC | Chlorophytes 18S rRNA gene | R |
| ONact1 [4] | GTGTGCGACAACGGCTCGGGC | *Volvox carteri* actin gene | F |
| ONact2 [4] | GCCTCCGTGAGAAGGACTGGGT | *V. carteri* actin gene | R |
| ONact1_CR | GTGTGCGACAATGGTTCGGGC | *Chlamydomonas reinhardtii* actin gene *IDA5* | F |
| CR_IDA5_R3 | GTGCTCAATGGGGTAGCGCAGA | *C. reinhardtii* actin gene *IDA5* | R |
| eveFA | ATCGACAATCGTTGAGGTCCTT | Scaffold 6 of *V. carteri* f. *nagariensis* strain EVE genome | F |
| eveFB | GTCCTTGCAGTCAAGAATAAGTCC | Scaffold 6 of *V. carteri* f. *nagariensis* strain EVE genome | F |
| eveFC [5] | GGGCTTGCTCCAATTTTAGTGA | Scaffold 6 of *V. carteri* f. *nagariensis* strain EVE genome/ rickettsial 16S rRNA gene | F |
| eveRD | ATTCACGCGGTATTGCTGGA | Scaffold 6 of *V. carteri* f. *nagariensis* strain EVE genome/ rickettsial 16S rRNA gene | R |
| eveFE | AGTAGGAGGAGGTGTAGCGATG | Scaffold 6 of *V. carteri* f. *nagariensis* strain EVE genome/ rickettsia *murB* gene | F |
| eveRG | TGGTTGCGAAGCACTCCTAATA | Scaffold 6 of *V. carteri* f. *nagariensis* strain EVE genome/ rickettsia *murB* gene | R |
| eveRH | CGCCTAAATTTTCCATATCAGC | Scaffold 6 of *V. carteri* f. *nagariensis* strain EVE genome/ rickettsia *murB* gene | R |
| eveRH2 | ATTTTCCATATCAGCAGCAGTAGC | Scaffold 6 of *V. carteri* f. *nagariensis* strain EVE genome/ rickettsia *murB* gene | R |
| eveRJ | CACCTAACTTGCTGAACGAATC | Scaffold 6 of *V. carteri* f. *nagariensis* strain EVE genome/ rickettsia *ddlB* gene | R |
| eveFZ | CAACGGACAGGGAGACGATTAC | Scaffold 6 of *V. carteri* f. *nagariensis* strain EVE genome | F |
| eveRY | CGCTTCTGCAAGCTATTTATATGG | Scaffold 6 of *V. carteri* f. *nagariensis* strain EVE genome | R |
| eveFX | TTTCGCGGATAGGAGGAATCTG | Scaffold 6 of *V. carteri* f. *nagariensis* strain EVE genome | F |
| eveRL | AGGCGGCTAGTCTTTTGTTG | Scaffold 6 of *V. carteri* f. *nagariensis* strain EVE genome | R |
| eveFK | CAGGCCCAGAAATGATGATAGC | Scaffold 6 of *V. carteri* f. *nagariensis* strain EVE genome | F |
| eveFM | TGGGAGCCGATATTGCTGTACT | Scaffold 6 of *V. carteri* f. *nagariensis* strain EVE genome/ rickettsia *ddlB* gene | F |
| eveRN | TGCTCGGCAAAACCTTCAGTA | Scaffold 6 of *V. carteri* f. *nagariensis* strain EVE genome/ rickettsia *ddlB* gene | R |
| eveRP | GGTAATGCTTGTGGAGCGAAG | Scaffold 6 of *V. carteri* f. *nagariensis* strain EVE genome | R |
| eveFQ | CCAGAATCGCCAAGTCTTTTCA | Scaffold 6 of *V. carteri* f. *nagariensis* strain EVE genome | F |
| eveRS | TGGCAAAGCAGTTACGAAGG | Scaffold 6 of *V. carteri* f. *nagariensis* strain EVE genome | R |
| eveRT | CGAGGTCTTTTGACCGGTTG | Scaffold 6 of *V. carteri* f. *nagariensis* strain EVE genome | R |
| eveFU | GGAGGTGTAGCGATGAATGC | Scaffold 6 of *V. carteri* f. *nagariensis* strain EVE genome/ rickettsia *murB* gene | F |
| eveFV | ATGTCTGCAGAACGCGAAG | Scaffold 6 of *V. carteri* f. *nagariensis* strain EVE genome/ rickettsia *ddlB* gene | F |
| eveRW | GCAATTTCTGGGCAAATAGATAA | Scaffold 6 of *V. carteri* f. *nagariensis* strain EVE genome/ rickettsia *ddlB* gene | R |
| eve-Jd-F1 | GCCCATTGTCACAAGGTTCATGCC | Scaffold 6 of *V. carteri* f. *nagariensis* strain EVE genome | F |
| asmb81_F4 | AAGAGGCGTGTACGGACGAGAATA | Scaffold 6 of *V. carteri* f. *nagariensis* strain EVE genome | F |
| asmb81_F5 | TACGGCGGAAAGTCAAAAACAGAT | Scaffold 6 of *V. carteri* f. *nagariensis* strain EVE genome | F |
| asmb82_R1 | TTCGATTTACCGAGCTCCAATGTT | Scaffold 6 of *V. carteri* f. *nagariensis* strain EVE genome | R |
| asmb82_R2 | TAATTTCACCCCGGACCTTAAACC | Scaffold 6 of *V. carteri* f. *nagariensis* strain EVE genome | R |
| asmb81_R6 | ATAGTGTTGTTGGCTGACGGATTG | Scaffold 6 of *V. carteri* f. *nagariensis* strain EVE genome | R |
| asmb82_F3 | CATCGTACCAACGGTACATCCAAG | Scaffold 6 of *V. carteri* f. *nagariensis* strain EVE genome | F |
| asmb82_F4 | GCAGGGTGAGTTGCACGAAGA | Scaffold 6 of *V. carteri* f. *nagariensis* strain EVE genome | F |
| asmb82_F5 | CAGGAACTACTCTCGCTCGGTCAG | Scaffold 6 of *V. carteri* f. *nagariensis* strain EVE genome | F |
| asmb82_R6 | GTATATTGGGTGCAGTCGGAGGAG | Scaffold 6 of *V. carteri* f. *nagariensis* strain EVE genome | R |
| ITS-Fa [6] | GGGATCCGTTTCCGTAGGTGAACCTGC | Internal transcribed spacer (ITS) region of nuclear rDNA | F |
| ITS-Rb [6] | GGGATCCATATGCTTAAGTTCAGCGGGT | ITS region of nuclear rDNA | R |
| ITS-Fc [6] | GCATCGATGAAGAACGCAGC | ITS region of nuclear rDNA | F |
| ITS-Rd [6] | GCTGCGTTCTTCAGCGAT | ITS region of nuclear rDNA | R |
| RcBl-murB-FA | ATTGGCATTGGCGGCGTTGG | Rickettsial *murB* | F |
| U2180-murB-FC | TCTGAATGGGCTACCAAAGAGG | Rickettsial *murB* | F |
| MkCc-murB-FE | TTTTCAGGGTATCCGAGGGGTA | Rickettsial *murB* | F |
| MkCc-murB-FG | TACCGTTATTGGTGCTGGCTCA | Rickettsial *murB* | F |
| MkCc-murB-FK | TCAGAAATGCACTGTAACTTCA | Rickettsial *murB* | F |
| RcMk-murB-FP | TATTCCGGGRACAGTAGGAGGAGG | Rickettsial *murB* | F |
| RcMk-murB-RK2 | AGTGCATTTCTGACATACATGC | Rickettsial *murB* | R |
| RcMk-murB-FQ | AATCTGAARCACCTAACYTGGCTTAAAG | Rickettsial *murB* | F |
| RcMk-murB-FK3 | CAGAAATGCACTGTAACTTCATGATTAAT | Rickettsial *murB* | F |
| MkPj-murB-RG | TGAACCAGCACCGATAACGGTA | Rickettsial *murB* | R |
| RcBl-ddlB-RB | GGGAAAATTCTTTTTGCTGTTTTA | Rickettsial *ddlB* | R |
| U2180-ddlB-RD | ATACCGCCGCACCTAAAGCA | Rickettsial *ddlB* | R |
| MkCc-ddlB-RH | CATGCCGGTAACACAATCAAGG | Rickettsial *ddlB* | R |
| MkCc-ddlB-RJ | TGGTGTCATACCAGGGTGAGTGTT | Rickettsial *ddlB* | R |
| RcMk-ddlB-R01 | GAAACTTCCCGTTCAGCAGACA | Rickettsial *ddlB* | R |
| RcMk-ddlB-R02 | GCAATATCTGCTCCCATATC | Rickettsial *ddlB* | R |
| MkCc-ddlB-RL | GACTTCCTTTTGTTTAAAGCAA | Rickettsial *ddlB* | R |
| MkCc-ddlB-RM | TCTTTGGTATAACCATCTGATTT | Rickettsial *ddlB* | R |
| RcMk-ddlB-FQ | YGGAGAAGACGGTTGCCT | Rickettsial *ddlB* | F |
| RcMk-ddlB-RH2 | CATRCCRGTAACACAATCAAGG | Rickettsial *ddlB* | R |
| RcMk-ddlB-RN | CCGAAGCYAAAACCCCAGGACC | Rickettsial *ddlB* | R |
| MkPj-ddlB-RP | TTTTTCYTTATATACAGTTTTAAAATTTCCGTTAG | Rickettsial *ddlB* | R |
| RcMk-ddlB-FH3 | CCTTGATTGTGTTACYGGYATGATAAG | Rickettsial *ddlB* | F |
| MkPj-ddlB-FJ2 | AGYTAATACTCATCCCGGTATGACAC | Rickettsial *ddlB* | F |
| RcMk-murC-FA | GGTTATTGCTATTTTCCAGCCKCA | Rickettsial *murC* | F |
| RcMk-murC-FB | TTATGATGGGTGCTGGTARTATTT | Rickettsial *murC* | F |
| RcMk-murC-FC | GATYTGATAGTTATGATGGGWGCWGG | Rickettsial *murC* | F |
| RcMk-murC-FD | CTTAATTCTCTTGCGGCCATTG | Rickettsial *murC* | F |
| RcMk-murC-R05 | TTTATCRGGTAATTGCCARGCCCA | Rickettsial *murC* | R |
| RcMk-murC-R6 | TGGYCTTGCACCTAATTTTAYCATTTC | Rickettsial *murC* | R |
| RcMk-murC-R7 | TACYGCAATAGTAGADGGRATTTTGA | Rickettsial *murC* | R |
| RcMk-murC-F08 | GTTAAGAGRCGTTTTACCAAAGTTTGT | Rickettsial *murC* | F |
| RcMd-murC-F09 | GTTAMAGAACGCCGGATAATTACTTACG | Rickettsial *murC* | F |
| RcMk-murC-F10 | GGYCTTGCYCCTACTGTTATAAACG | Rickettsial *murC* | F |
| RcMk-murC-R10 | CGTTTATAACAGTAGGRGCAAGRCC | Rickettsial *murC* | R |
| MkPj-murC-F11 | TTGGATATTTATGGGGCAGGGGAAA | Rickettsial *murC* | F |
| RcMk-ftsQ-R01 | ATTGGCGTTCGCTCAGTAATCG | Rickettsial *ftsQ* | R |
| RcMk-ftsQ-R02 | GCATCCTGACCCACAACGTGTA | Rickettsial *ftsQ* | R |
| RcMk-ftsQ-F03 | TGAAGAAGGTAATCGAATCAGCTMA | Rickettsial *ftsQ* | F |
| MkCc-ccmF-R01 | CCTCCCCAGCCAAGTTCCCTAT | Rickettsial *ccmF* | F |
| MkCc-ccmF-R02 | CAATAGCCTCCCCAGCCAAGTTCC | Rickettsial *ccmF* | F |
| MkCc-phbB-F01 | CAAGCCGGACAAATAGGACAGACT | Rickettsial *phbB* | R |
| MkCc-phbB-F02 | AAGACTTGGCCGCCCTGAAGAA | Rickettsial *phbB* | R |
| MkCc-phbB-F03 | GCCGCCCTGAAGAAGTAGCAAG | Rickettsial *phbB* | R |
| PjMk-phbB-TF1 | AATGCTGCCTATTGCTTCAGGAAA | Rickettsial *phbB* | F |
| RcMk-phbB-F4 | CCAAAATGACTGATGATTAGCRAGAAC | Rickettsial *plsC* | R |
| RcMk-phbB-R5 | ATGTTCCCAGAAGGYGGYACRG | Rickettsial *plsC* | F |

References

1. Weisburg WG, Barns SM, Pelletier DA, Lane DJ (1991) 16S ribosomal DNA amplification for phylogenetics study. J Bacteriol 173: 697–703.

2. Kawafune K, Hongoh Y, Hamaji T, Nozaki H (2012) Molecular identification of rickettsial endosymbionts in the non-phagotrophic volvocalean green algae. PLoS ONE 7: e31749.

3. Nakazawa A, Nozaki H (2004) Phylogenetic analysis of the tetrasporalean genus *Asterococcus* (Chlorophyceae) based on 18S ribosomal RNA gene sequences. J Jpn Bot 79: 255–261.

4. Kobl I, Kirk DL, Schmitt R. (1998) Quantitative PCR data falsify the chromosomal endoreduplication hypothesis for *Volvox carteri* (Volvocales, Chlorophyta). J Phycol 34: 981-988.

5. Kawafune K, Hongoh Y, Nozaki H (2014) A rickettsial endosymbiont inhabiting the cytoplasm of *Volvox carteri* (Volvocales, Chlorophyceae). Phycologia 53: 95–99.

6. Coleman AW (1994) Molecular delination of species and syngens in Volvocacean green algae. J Phycol 30: 80–90.
