# Supplementary material for: Two Different Rickettsial Bacteria Invading Volvox carteri
Source: PLoS One. 2015 Feb 11;10(2):e0116192. doi: 10.1371/journal.pone.0116192 (PMC4324946; doi:10.1371/journal.pone.0116192)
Supplement: S3 Table — DDBJ/EMBL/GenBank accession numbers of nuclear ribosomal DNA internal transcribed spacer regions and rickettsial gene-like sequences determined in this study are also shown. (DOC) [file pone.0116192.s010.doc]

**Table S3. List of *Volvox carteri* and other algal strains used in this study.** DDBJ/EMBL/GenBank accession numbers of ribosomal DNA internal transcribed spacer region (ITS) sequences and rickettsial gene-like sequences determined in this study are also presented.

| Taxon | Strain designation | Accession No. | Rickettsial gene/gene-like sequences |  |
| --- | --- | --- | --- | --- |
|  |  | ITS sequence | From rickettsiae-lacking strains | From rickettsiae-containing strains |
| *Volvox carteri* f. *nagariensis* | EVE | LC004701^a^ | LC004713^a^ | - |
|  | UTEX^b^ 1886 | LC004702^a^ | LC004714^a^ | - |
|  | NIES^c^-397 | LC004703^a^ | LC004715^a^ | - |
|  | NIES-398 | LC004704^a^ | LC004716^a^ | - |
|  | UTEX 2903 | LC004705^a^ | - | - |
| *Volvox carteri* f. *kawasakiensis* | NIES-732 | LC004706^a^ | LC004717^a^ | - |
|  | NIES-733 | LC004707^a^ | LC004718^a^ | - |
| *Volvox carteri* f. *weismannia* | UTEX 1874 | LC004708^a^ | - | - |
|  | NIES-866  (=UTEX 1875) | AB771953 | LC004719^a^ | - |
|  | UTEX 1876 | AB771954 | LC004720^a^ | - |
|  | UTEX 2170 | LC004709^a^ | LC004721^a^ | - |
|  | UTEX 2904 | LC004710^a^ | - | - |
|  | UTEX 2180 | LC004711^a^ | - | LC004722^a^ |
| *Volvox obversus* | UTEX 1865 | LC004712^a^ | - | - |
| *Pleodorina japonica* | NIES-577 | - | - | LC004723^a^ |
| *Carteria cerasiformis* | NIES-424 | - | - | - |
|  | NIES-425 | - | - | LC004724^a^ (including 16S rRNA);  LC004725^a^ (including *murB*, *ddlB*, etc.) |
| *Chlamydomonas reinhardtii* | CC^d^-503  (*cw92* mt+) | - | - | - |

^a^Sequence determined in this study.

^b^Microbial Culture Collection at the National Institute for Environmental Studies, Japan [1].

^c^Culture Collection of Algae at the University of Texas at Austin (Austin, TX, USA; http://www.utex.org/).

^d^Chlamydomonas Resource Center (St. Paul, MN, USA; http://chlamycollection.org/).

References

1. Kasai F, Kawachi M, Erata M, Mori F, Yumoto K, et al. (2009) NIES-Collection. List of Strains. 8th Edition. Jpn J Phycol (Sôrui) 57: suppl.1–350, pls1–7.
