# Supplementary material for: Two Different Rickettsial Bacteria Invading Volvox carteri
Source: PLoS One. 2015 Feb 11;10(2):e0116192. doi: 10.1371/journal.pone.0116192 (PMC4324946; doi:10.1371/journal.pone.0116192)
Supplement: S4 Table — (DOC) [file pone.0116192.s011.doc]

**Table S4. DDBJ/EMBL/GenBank accession numbers of the rickettsial *murB* and *ddlB* genes/gene-like sequences used in this study.**

| Taxon | Accession No. |  |
| --- | --- | --- |
|  | *murB* | *ddlB* |
| *Rickettsia* *massiliae* str. MTU5 | NC_009900 | NC_009900 |
| RIckettsia *massiliae* str. AZT80 | - | NC_016931 |
| *Rickettsia* *rickettsii* str. lowa | NC_010263.2 | NC_010263.2 |
| *Rickettsia* *conorii* str. Malish 7 | AE006914 | AE006914 |
| *Rickettsia* *japonica* str. YH | NC_016050 | NC_016050 |
| *Rickettsia* *slovaca* str. D-CWPP | CP003375 | CP003375 |
| *Rickettsia* *slovaca* str. 13-B | - | CP002428 |
| *Rickettsia* *parkeri* str. Portsmouth | CP003341 | CP003341 |
| *Rickettsia* *rhipicephali* str. 3-7-female6-CWPP | CP003342 | CP003342 |
| *Rickettsia* *heilongjiangensis* str. 54 | CP002912 | CP002912 |
| *Candidatus* Rickettsia amblyommii str. GAT-30V | CP003334 | CP003334 |
| *Rickettsia* *philipii* str. 364D | CP003308 | CP003308 |
| *Rickettsia* *africae* str. ESF-5 | CP001612 | CP001612 |
| *Rickettsia* *montanensis* str. OSU 85-930 | CP003340 | CP003340 |
| *Rickettsia* *peacockii* str. Rustic | CP001228 | CP001228 |
| *Rickettsia* *typhi* str. Wilmington | NC_006142 | NC_006142 |
| *Rickettsia* *prowazekii* str. MadridE 2/4 | AJ235271 | AJ235271 |
| *Rickettsia* *australis* str. Cutlack | CP003338 | CP003338 |
| *Rickettsia* *akari* str. Hartford chromosome | NC_009881 | NC_009881 |
| *Rickettsia* *felis* str. URRWXCal2 | NC_007109 | NC_007109 |
| *Rickettsia* *canadensis* str. McKiel | NC_009879 | NC_009879 |
| *Rickettsia* *canadensis* str. CA410 | CP003304 | CP003304 |
| *Rickettsia* *bellii* str. OSU 85-389 | NC_009883 | NC_009883 |
| *Rickettsia* *bellii* str. RML369-C | NC_007940 | NC_007940 |
| *Orientia tsutsugamushi* str. Boryong | NC_009488 | NC_009488 |
| *Orientia tsutsugamushi* str. Ikeda | NC_010793 | NC_010793 |
| Endosymbiont of *Carteria cerasformis* NIES-425 | LC004725^a^ | LC004725 |
| Endosymbiont of *Pleodorina japonica* NIES-577 | LC004723^a^ | LC004723 |
| Endosymbiont of *Volvox carteri* f. *weismannia* UTEX 2180 | LC004722^a^ | LC004722 |
| *Volvox carteri* f*. nagariensis* EVE^b^ | LC004713^a^ | LC004713 |
| *Volvox carteri* f. *nagariensis* UTEX 1886^b^ | LC004714^a^ | LC004714 |
| *Volvox carteri* f. *nagariensis* NIES-397^b^ | LC004715^a^ | LC004715 |
| *Volvox carteri* f. *nagariensis* NIES-398^b^ | LC004716^a^ | LC004716 |
| *Volvox carteri* f. *weismannia* NIES-866^b^ (=UTEX 1875) | LC004719^a^ | LC004719 |
| *Volvox carteri* f. *weismannia* UTEX 1876^b^ | LC004720^a^ | LC004720 |
| *Volvox carteri* f. *weismannia* UTEX 2170^b^ | LC004721^a^ | - |

^a^Sequence determined in this study.

^b^Strain lacking rickettsial endosymbionts.
